# Design of a potent interleukin-21 mimic for cancer immunotherapy

**DOI:** 10.1126/sciimmunol.adx1582

**Published:** 2025-09-26

**Authors:** Jung-Ho Chun, Birkley S. Lim, Suyasha Roy, Michael J. Walsh, Gita C. Abhiraman, Kevin Zhangxu, Tavus Atajanova, Or-Yam Revach, Elisa C. Clark, Peng Li, Claire A. Palin, Asheema Khanna, Samantha Tower, Rakeeb Kureshi, Megan T. Hoffman, Tatyana Sharova, Aleigha Lawless, Sonia Cohen, Genevieve M. Boland, Tina Nguyen, Frank Peprah, Julissa G. Tello, Samantha Y. Liu, Chan Johng Kim, Hojeong Shin, Alfredo Quijano-Rubio, Kevin M. Jude, Stacey Gerben, Analisa Murray, Piper Heine, Michelle DeWitt, Umut Y. Ulge, Lauren Carter, Neil P. King, Daniel-Adriano Silva, Hao Yuan Kueh, Vandana Kalia, Surojit Sarkar, Russell W. Jenkins, K. Christopher Garcia, Warren J. Leonard, Michael Dougan, Stephanie K. Dougan, David Baker

**Affiliations:** 1. Institute for Protein Design, University of Washington, Seattle, WA; 2. Department of Biochemistry, University of Washington School of Medicine, Seattle, WA; 3. Graduate Program in Biological Physics, Structure and Design, University of Washington, Seattle, WA; 4. Department of Medicine, Division of Gastroenterology, Massachusetts General Hospital, Boston, MA; 5. Department of Cancer Immunology and Virology, Dana-Farber Cancer Institute, Boston, MA; 6. Laboratory of Molecular Immunology and the Immunology Center, National Heart, Lung, and Blood Institute, National Institutes of Health, Bethesda, MD; 7. Department of Molecular and Cellular Physiology, Stanford University School of Medicine, Stanford, CA; 8. Mass General Cancer Center, Krantz Family Center for Cancer Research, Department of Medicine, Massachusetts General Hospital, Harvard Medical School, Boston, MA; 9. Broad Institute of MIT and Harvard, Cambridge, MA; 10. Department of Bioengineering and Institute for Stem Cell and Regenerative Medicine, University of Washington, Seattle, WA; 11. Ben Towne Center for Childhood Cancer Research, Seattle Children’s Research Institute, Seattle, WA; 12. Department of Immunology, Harvard Medical School, Boston, MA; 13. Massachusetts General Hospital Cancer Center, Harvard Medical School, Boston, MA; 14. Department of Surgery, Massachusetts General Hospital, Boston, MA; 15. Department of Pediatrics, University of Washington, Seattle, WA; 16. Department of Pathology, University of Washington, Seattle, WA; 17. Howard Hughes Medical Institute, Stanford University School of Medicine, Stanford, CA; 18. Department of Medicine, Harvard Medical School, Boston, MA; 19. Howard Hughes Medical Institute, University of Washington, Seattle, WA

## Abstract

Long-standing goals of cancer immunotherapy are to activate cytotoxic antitumor T cells across a range of affinities for tumor antigens while suppressing regulatory T cells. Computational protein design has enabled the precise tailoring of proteins to meet specific needs. Here, we report a *de novo* IL-21 mimic, 21h10, designed to have high stability and signaling potency in humans and mice. In murine and *ex vivo* human organotypic tumor models, 21h10 showed robust antitumor activity, with prolonged signaling and stronger antitumor activity compared to native IL-21. 21h10 induced pancreatitis that could be mitigated by TNF blockade without compromising antitumor efficacy. Although anti-drug antibodies to 21h10 formed, they were not neutralizing. 21h10 induced highly cytotoxic T cells with a range of affinities, robustly expanding intratumoral low-affinity cytotoxic T cells and driving high expression of IFN-*γ* and granzyme B compared to native IL-21, while increasing the frequency of IFN-*γ*^+^ T helper 1 cells and reducing regulatory T cells. As 21h10 has full cross-reactivity, high stability and potency, and potentiates low-affinity antitumor responses, it has translational potential.

## One sentence summary

A *de novo* IL-21 mimic, 21h10, exhibits improved stability and potent antitumor activity *in vivo* compared to native IL-21.

## INTRODUCTION

Interleukin (IL)-21 plays a key role in the differentiation of effector T cells(1–4), T follicular helper (T_FH_) cells(5), B cells(6, 7), natural killer (NK) cells(3), macrophages(8), and dendritic cells(9, 10). IL-21 promotes the proliferation and survival of CD8^+^ T cells and the generation of memory CD8^+^ T cells(10–13). In addition, IL-21 enhances the effector functions of CD8^+^ T cells(10, 14) and cooperates with IL-15 to further promote the expansion of these cells(4). Multiple murine tumor models and early-stage clinical trials(15, 16) of recombinant IL-21 have been completed in several types of cancer, including melanoma(17–22), renal cell carcinoma(21–23), ovarian cancer(24), and non-Hodgkin’s lymphoma(25), both as a single agent and in combination with immune checkpoint inhibitors and cancer vaccines. These trials showed some evidence of efficiency, although less than contemporaneous trials of immune checkpoint inhibitors. IL-21 was also shown to be more effective than IL-2 when PMEL-1 TCR transgenic CD8^+^ T cells were cultured *in vitro* with cytokines prior to adoptive transfer into B16F10 melanoma-bearing mice(26). Although the initial clinical testing of IL-21-based cancer immunotherapies has been encouraging, the precise effect of IL-21 on antitumor responses remains incompletely understood. Additionally, IL-21 has low stability, resulting in suboptimal pharmacokinetic properties, and the limited antitumor activity of human IL-21 (hIL-21) in mice has complicated the use of animal models to predict the toxicity and activity of candidate IL-21 therapeutics in humans(27–31).

Inspired by previous success in designing *de novo* IL-2 mimics for cancer immunotherapy(32, 33), we reasoned that a *de novo* protein mimic of IL-21 with augmented stability that potently induces signaling in both humans and mice could overcome the limitations of native IL-21. We set out to use computational protein design to create IL-21 mimics with improved therapeutic properties. We developed 21h10, which is highly stable *in vivo*, shows efficacy against multiple mouse models of cancer, and activates human T cells, including tumor infiltrating lymphocytes, from patients with refractory melanoma.

## RESULTS

### Computational protein design of an IL-21 mimic

**Fig. 1.**
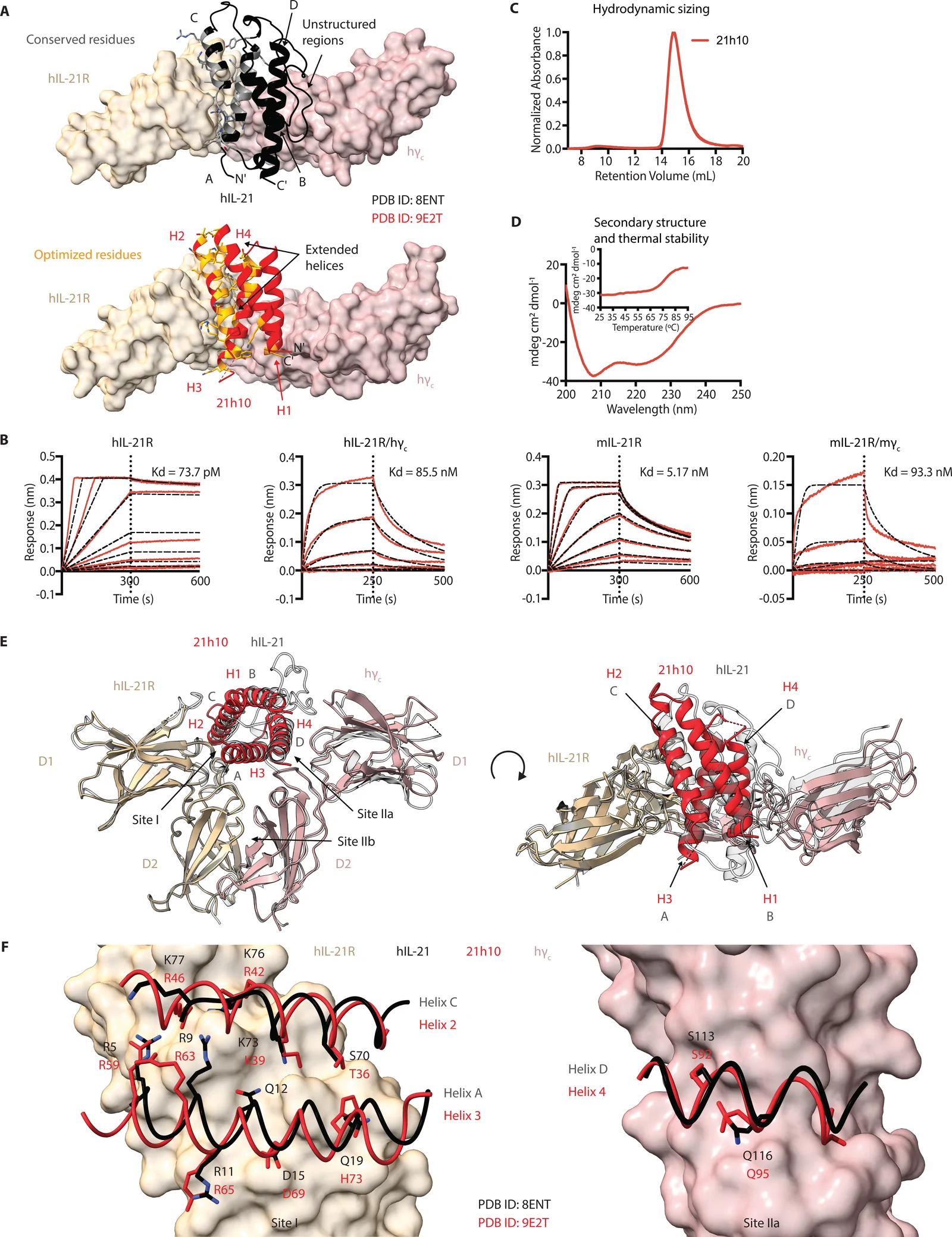
Computationally designed IL-21 mimic recapitulates receptor interactions of native IL-21 with superior stability and human/murine cross-reactivity. (A) The optimized IL-21 mimic, 21h10, is designed by recapitulating the helical bundle structure of the native hIL-21. Unstructured regions in the hIL-21 are removed, and short helices are extended to accommodate improved intramolecular packing. Some of the interface residues are conserved from the native hIL-21 for the design of initial hits. Other residues from the *de novo* scaffolds are further optimized, as shown. The upper panel is top-view of hIL-21 in complex with hIL-21R/h*γ*_c_ (PDB ID: 8ENT) with residues that are conserved during initial design of IL-21 mimic are colored in gray. The lower panel is top-view of 21h10 in complex with hIL-21R/h*γ*_c_ (PDB ID: 9E2T) with the optimized residues that are optimized are colored in yellow. (B) Association and dissociation of 21h10 to human and murine IL-21R and *γ*_c_ show concentration-dependent binding curves of 21h10. Black dotted lines show curve fits of raw data using the Mass Transport model. (C) Size-exclusion chromatography of 21h10 using Superdex 75 10/300 GL column. (D) Wavelength scan from 200 nm to 250 nm for measuring 𝛼-helical secondary structure. Wavelength scan at 222 nm from 25°C to 95°C for measuring thermal stability.. (E) Crystal structure of 21h10 in complex with hIL-21R and h*γ*_c_ (PDB ID: 9E2T). (F) 21h10 conserves key molecular interactions in the site I interface (left) and site IIa interface (right) observed in the structure of the human IL-21 receptor complex. (PDB ID: 8ENT).

IL-21 signals by inducing heterodimerization of the IL-21 receptor (IL-21R) with the common cytokine receptor *γ* chain (*γ*_c_), leading to the activation of JAK1 and JAK3, which are associated with the IL-21R and *γ*_c_ intracellular domains, respectively. This signaling results in the phosphorylation and activation of STAT transcription factors(34). Native IL-21 makes extensive interfaces with both receptor subunits (Fig. 1A) involving largely helical secondary structures, but the “backside” of IL-21 contains two long loops that we reasoned might reduce the stability of the molecule. Traditional protein engineering approaches that use directed evolution to identify small numbers of sequence changes are not able to replace extended structural segments such as these poorly ordered regions. Instead, we sought to construct mimics that retain the receptor-interacting interfaces, but with a more well-ordered and less protease-susceptible overall structure. As the structure of the full complex was not initially available, we began from the human IL-21/IL-21R (hIL-21/hIL-21R) complex structure(35) and docked the human *γ*_c_ (h*γ*_c_) from the human IL-2 complex structure(36) on the hIL-21/hIL-21R complex structure. We then generated helical protein scaffolds with regions that superimpose perfectly on the receptor-interacting segments of native IL-21, but with improved inter-helix packing, short connecting loops, and minimal unstructured regions.

Wild-type hIL-21 consists of four helices, with helix A (the first helix from the N-terminus) and helix C (the third helix) interacting with hIL-21R, and helix D (the fourth helix) interacting with h*γ*_c_(36, 37). Helix B (the second helix) and helix C are not long enough to form ideal intramolecular interactions and intermolecular contacts with the receptor chains, so we replaced the two long unstructured regions between helix A/helix B and helix C/helix D by extending helix B and helix C. We sampled different helical bundle “up-down-up-down” topologies by testing different connectivities between the helices; these differ from the “up-up-down-down” topology of native IL-21, and allow more ideal helical packing and loop geometries. Sequences were designed for these backbones in the context of hIL-21R using *Rosetta*, retaining the residues interacting with hIL-21R and anticipated to interact with h*γ*_c_. We stochastically generated designs with these properties using *Rosetta* helical sampling and loop-building methodology, and selected for experimental characterization to validate their folding to the designed structure (fig. S1A).

### IL-21 mimic, 21h10, binds to human and murine IL-21 receptors

We obtained synthetic genes for the selected designs and assessed binding to hIL-21R using yeast surface display. Four designs bound hIL-21R, but none bound to h*γ*_c_ in the presence of hIL-21R (fig. S1B and table S1); this was not unexpected due to the unknown (at the time) h*γ*_c_ structure, which was not incorporated in the design stage. From a mutagenesis library around the tightest binder, 21d26, we identified a variant, 21JC15, that binds h*γ*_c_ weakly in an hIL-21R-dependent manner, as well as murine *γ*_c_ (m*γ*_c_) in a murine IL-21R (mIL-21R)-dependent manner (table S1). Further interface optimization guided by site-saturation mutagenesis (SSM) and combinatorial library screening (fig. S1C) resulted in 21h10 with high affinity for both the human and murine IL-21 receptor complexes (Fig. 1B, and tables S1 and S2). 21h10 is shorter than hIL-21 (102 vs. 131 amino acids) with sequence identity only in the conserved interface regions(38, 39). 21h10 was expressed at high levels in *Escherichia coli*, monodisperse in size-exclusion chromatography (Fig. 1C), and has a helical circular dichroism (CD) structure consistent with the design model. CD melting experiments revealed that 21h10 has high thermal stability, with a T_m_ around 75°C (Fig. 1D).

Copurification of 21h10 with the hIL-21R extracellular domain, followed by mixing this complex with the extracellular domain of h*γ*_c_ (amino acids 33–232), resulted in the growth of crystals that diffracted anisotropically with resolution between 2.3 Å and 3.4 Å (fig. S2 and table S3). Solution of the structure by molecular replacement revealed the structure of 21h10 in complex with hIL-21R and h*γ*_c_ to be a Y-shaped receptor assembly similar to the wild-type hIL-21 complex(37). 21h10 binds to hIL-21R at site I through helices 2 and 3, burying a surface area of 870 Å^2^, and it binds to h*γ*_c_ at site IIa through helix 4, burying a surface area of 423 Å^2^ (Fig. 1E and table S4). The key molecular contacts mediating receptor interaction are strongly conserved. At the site I interface, 21h10 interacts with hIL-21R through hydrogen bonds at residues K39, R46, R59, R63, and R65, and salt bridges at residues R42, R46, R53, and D69 (table S4). These side chains of 21h10 are nearly identically positioned to the corresponding side chains in hIL-21 despite different molecular topology (Fig. 1F). Similarly, at the site IIa interface, 21h10 binds to h*γ*_c_ through sparse hydrogen bonding at S92 and Q95 on helix 4 (table S4). These two residues are precisely conserved in helix D of hIL-21 (Fig. 1F), reflecting the ability of *de novo* protein design to reproduce important molecular interactions in a protein-protein interface accurately.

### 21h10 potently induces STAT phosphorylation and effector CD8^+^ T cell differentiation

**Fig. 2.**
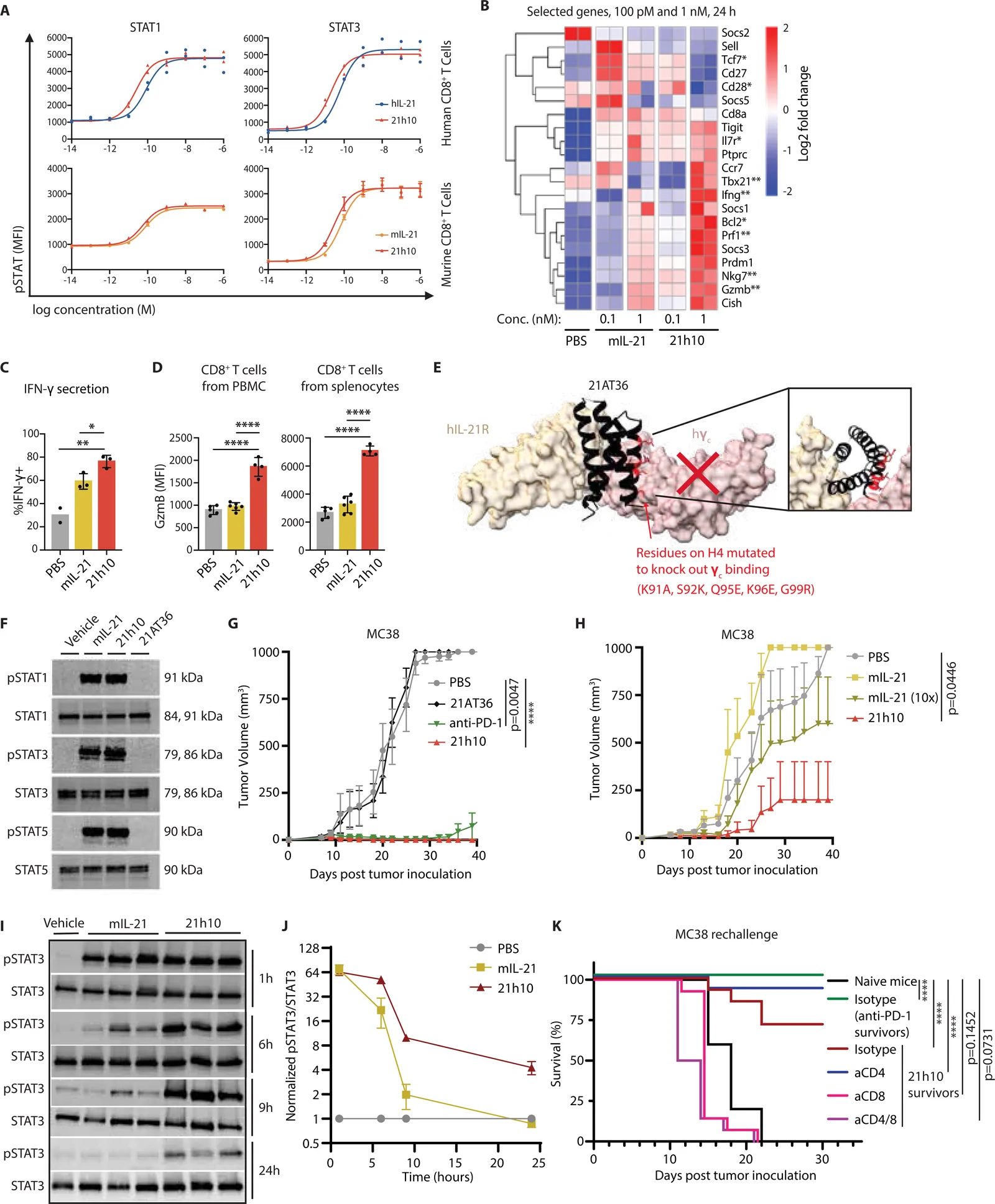
21h10 shows enhanced STAT signaling, gene expression, cellular phenotypes, and *in vivo* antitumor activity. (**A**) Human and murine CD8^+^ T cells were treated with native IL-21 and 21h10 for 20 minutes (human n=2, murine n=3). (**B**) Genes related to cellular signaling and phenotype are compared for expression levels between murine CD8^+^ T cells treated with PBS, mIL-21, or 21h10 at 100 pM or 1 nM (n=2). Scale in log2-fold change. * and ** indicate memory-related and effector-related genes, respectively. After 24-hour treatment with the cytokines, cells were harvested for RNA-sequencing. (**C**) The percentage of murine IFN-*γ*^+^ CD8^+^ T cells upon treating PBS, mIL-21, or 21h10 at 1 nM. Significance was determined using one-way ANOVA with Tukey’s correction. (**D**) Granzyme B expression of LCMV-specific CD8^+^ T cells from PBMC and splenocytes were measured 7 days after infection from LCMV-inoculated mice with daily injections of PBS, mIL-21 (2.5 μg), or 21h10 (2.5 μg) for 7 days. Significance was determined using lognormal one-way ANOVA with Tukey’s multiple comparison test. (**E**) Computational model of 21AT36 with mutated residues highlighted in red. *γ*_c_ is illustrated to show position of the removed *γ*_c_ interface. (**F**) Western blot for STAT and pSTAT with vehicle (PBS), mIL-21, 21h10, and 21AT36 in TRP1^high^ CD8^+^ T cells. Representative of two experiments. (**G**) MC38-bearing mice were treated daily with PBS, 21AT36, 21h10, or anti-PD-1 for 14 days. Representative of three experiments. Significance was determined using Brown-Forsythe and Welch one-way ANOVA with Dunnett’s correction using AUC values for each mouse versus PBS group. (**H**) Mice were inoculated with MC38 and treated with PBS, mIL-21, 10-fold dose mIL-21, and 21h10. Significance was determined using Brown-Forsythe and Welch ANOVA with Dunnett’s correction using AUC values for each mouse. (**I**) Western blot for STAT3 and pSTAT3 in the spleens of mice treated with vehicle (PBS), mIL-21, and 21h10. Molecular weights of the bands are identical to (**F**). Representative of two experiments. (**J**) Normalized pSTAT3/STAT3 in spleens of treated mice over 24 hours from (**I**). (**K**) MC38-rechallenge with MC38-survivor mice from previous 21h10 or anti-PD-1 treatment. Significance was determined in Kaplan-Meier survival curves using Mantel-Cox log-rank tests comparing each group to the naive controls.

Upon receptor binding, native IL-21 mediates downstream signaling by phosphorylating STAT1 and STAT3 in T cells(40). While hIL-21 showed low cross-reactivity in murine cells (fig. S3A and table S5), 21h10 treatment *in vitro* led to phosphorylation of STAT1 and STAT3 with potency equivalent to native hIL-21 and mIL-21 in both human and murine CD8^+^ T cells, CD4^+^ T cells, and B cells (Fig. 2A, fig. S3B, and table S6).

Native IL-21 promotes effector CD8^+^ T cell differentiation in human CD8^+^ T cells *ex vivo*(3, 41) and contributes to effector T cell (CD44^+^CD62L^-^) generation *in vivo*(42). To investigate the effects of 21h10 on gene expression and effector CD8^+^ T cell differentiation, we cultured murine CD8^+^ T cells in media containing serum with 100 pM, 1 nM, 10 nM, and 100 nM of mIL-21 or 21h10 and analyzed gene expression by RNA-sequencing. 21h10 increased expression of effector molecules (*Tbx21*, *Prdm1*, *Prf1*, *Gzmb*) while reducing the expression of the genes associated with a resting state (*Tcf7*, *Sell*) as compared to mIL-21 at 100 pM and 1 nM. 100 pM of 21h10 elicited a similar gene expression profile as 1 nM of mIL-21 (Fig. 2B and fig. S3C); gene expression patterns were similar but not identical at higher doses (fig. S3D). 21h10 elicited similar or higher IL-21R and granzyme B protein expression levels in human and murine CD8^+^ T cells compared to hIL-21 and mIL-21, respectively (fig. S4A), and proliferation of murine CD8^+^ T cells at 1 nM and 100 pM of 21h10 was enhanced compared to mIL-21 (fig. S4, B to C). 21h10 promoted maximal differentiation of murine CD8^+^ T cells into CD44^+^CD62L^−^ effector T cells at 1 nM, while mIL-21 showed a similar effect at 100 nM (fig. S4D) accompanied by a robust increase in the secretion of interferon-*γ* (IFN-*γ*) at 1 nM (Fig. 2C). In mice infected with lymphocytic choriomeningitis virus (LCMV), daily intraperitoneal (i.p.) injection of 2.5 μg of 21h10 for 7 days induced granzyme B expression in virus-specific CD8^+^ T cells in peripheral blood and spleen harvested on day 7, while mIL-21 had minimal effects (Fig. 2D); thus, 21h10 enhances effector CD8^+^ T cell differentiation both *in vitro* and *in vivo*.

### 21h10 has potent antitumor activity in murine and human organotypic tumor models

Using ProteinMPNN(43), we redesigned the *γ*_c_-interface on helix H4 of 21h10 to abolish its receptor affinity, resulting in an antagonist, 21AT36, which binds to IL-21R but not *γ*_c_ (Fig. 2E, fig. S5A, and table S1). While mIL-21 and 21h10 induced phosphorylation of STAT1, STAT3, and STAT5, 21AT36 did not induce STAT phosphorylation in murine CD8^+^ T cells (Fig. 2F and fig. S5B). In mice bearing subcutaneous MC38 adenocarcinoma tumors(44), daily intraperitoneal (i.p.) treatment with 15 μg of 21h10 led to rapid tumor regression similar to PD-1 blockade, whereas equimolar dose of 21AT36, as expected, did not show antitumor activity (Fig. 2G and fig. S6A).

21h10 exhibited enhanced antitumor activity in the MC38 model compared to native IL-21 despite similar activity in cell culture, outperforming both a molar equivalent and a 10-fold molar excess of native IL-21 (Fig. 2H and fig. S7, A to B). We hypothesized that this increased activity was due to the enhanced stability of 21h10, resulting in resistance to serum proteases, longer receptor occupancy time, and prolonged *in vivo* signaling. To test this, we treated mice with a single intravenous dose of native mIL-21 or 21h10 and measured pSTAT3 in splenocytes over time. Despite similar initial levels of cytokine-induced pSTAT3, native mIL-21-induced pSTAT3 decreased rapidly with time, whereas 21h10-induced pSTAT3 declined more slowly and remained detectable at 24 hours after injection (Fig. 2, I to J). 21h10 also retained greater *in vitro* activity after incubation with murine serum compared to native mIL-21 (fig. S8A), consistent with the stronger phenotypic effects and slower binding reduction of 21h10 compared to native mIL-21 during prolonged *in vitro* incubation in serum-containing media (figs. S4 and S8B).

The improved *in vivo* activity of 21h10 was not due to off-target binding, as MC38 challenge in *Il21r*−/− mice showed no efficacy (fig. S6B). 21h10 treatment induced immune memory in mice that cleared MC38 tumors, as animals rechallenged with MC38 tumors were protected. Memory responses were dependent on CD8^+^ T cells, as protection was lost upon CD8^+^ and CD8^+^/CD4^+^ T cell depletion, but not in animals depleted of CD4^+^ T cells alone (Fig. 2K). *In vitro*, murine CAR-T cells targeting human CD19 (hCD19) generated in the presence of 21h10 exhibited enhanced cell expansion and cytotoxicity against B16 melanoma engineered to express hCD19, with increased expression of CD25, granzyme B, and Bcl2 (fig. S9, A to C), a shift in cellular metabolism to a high metabolic state, and rapid elimination of MC38 adenocarcinoma when subjected to multiple rounds of tumor injection (fig. S9, D to F).

**Fig. 3.**
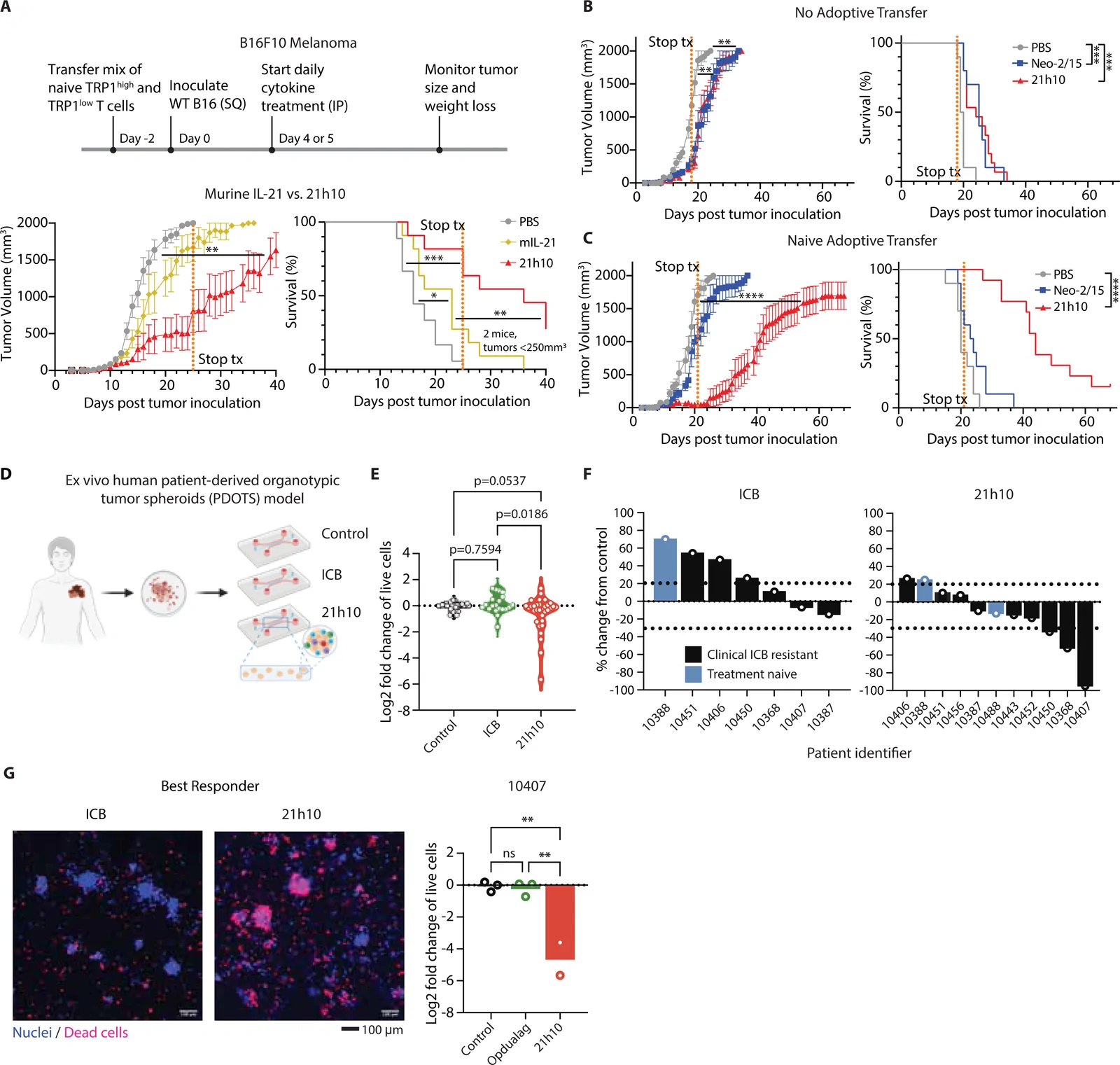
21h10 improves antitumor efficacy in *in vivo* B16F10 murine melanoma and *ex vivo* human PDOTs models. (**A**) Naïve CD8^+^ TRP1^high/low^ melanoma-specific T cells (500,000 cells) were adoptively transferred to mice prior to B16F10 inoculation. Cytokine therapy began on day 5 and continued every day until the stop of treatment (dashed line). (**B**) Tumor volume in mice that received no prior T cell adoptive transfer before cytokine therapy. (**C**) Same as (**B**), but with adoptive transfer of naïve CD8^+^ TRP1^high/low^ T cells. For (**A**)–(**C**), Significance was determined using Brown-Forsythe and Welch one-way ANOVA with Dunnett’s correction using AUC values for each mouse versus PBS group. Significance was determined in Kaplan-Meier survival curves with Bonferroni-corrected Mantel-Cox log-rank tests. (**D**) Scheme of PDOTS preparation. Figure created with BioRender. (**E**) Microscopy-based viability assessment of *ex vivo* human melanoma PDOTS following treatment of ICB (anti-PD-1, pembrolizumab, or anti-PD-1+anti-LAG-3), 21h10, or untreated control, showing aggregated data of 11 PDOTS with three technical repeats for each. Significance was determined using one-way ANOVA with Tukey’s correction. (**F**) Viability assessment in (**E**) presented on a per-patient basis. The lower dashed line indicates tumor response (over 30% reduction from control). The upper dashed line indicates tumor growth (over 20% elevation from control). (**G**) Representative images and viability assessment of the best responder PDOTS to 21h10 (10407 in (**F**)). Significance was determined using one-way ANOVA with Tukey’s correction. PI-dead cells in red and Hoechst-nuclei in blue.

To determine the generalizability of our findings in MC38 adenocarcinoma, we assessed 21h10 in a less immunogenic tumor model. B16F10 is a model of poorly immunogenic melanoma known to respond to various immunotherapies, including cytokine therapies(32, 33). Responses to B16F10 can be augmented through the transfer of tumor-specific CD8^+^ T cells that recognize the TRP1 self-antigen with high or low affinity (TRP1^high^ and TRP1^low^ T cells, respectively), although these cells are insufficient to cause tumor regression on their own(45). Transferring TRP1^high^ and TRP1^low^ T cells models an oligoclonal T cell response to a tumor-associated self-antigen, analogous to what is observed in immunogenic human melanoma. We adoptively transferred TRP1^high/low^ T cells and treated B16F10 melanoma-bearing mice with PBS, mIL-21, or 21h10. mIL-21 treatment delayed tumor growth compared to PBS; however, as we observed for MC38, 21h10 treatment exhibited more robust tumor regression, with several long-term surviving animals (Fig. 3A and fig. S10A). Moreover, 21h10 synergized with adoptive cell transfer of TRP1^high/low^ cells, as compared to no adoptive transfer, and 21h10 had stronger antitumor activity than was previously observed for the IL-2/IL-15 mimic Neo-2/15(32, 33) (Fig. 3, B to C). Strikingly, 21h10 treatment combined with adoptive T cell transfer resulted in complete tumor regression in 2 out of 13 animals (fig. S10, B to C). We found similarly enhanced B16F10 antitumor responses when 21h10 was combined with the anti-melanoma antibody TA99(32, 33) (fig. S11A). *Ex vivo* treatment of TRP1^high/low^ T cells with 21h10 for 2 days prior to transfer into B16F10-bearing mice only modestly decreased tumor growth (fig. S11B), suggesting that sustained signaling in the tumor microenvironment may be important for optimal antitumor(46, 47) actions of 21h10.

To determine whether 21h10 had activity against human tumors, we used patient-derived organotypic tumor spheroids (PDOTS) model(48, 49). PDOTS, unlike organoids, are fresh tumor explants with an intact immune compartment for *ex vivo* profiling of immunotherapy response of human tumors(48). PDOTS were derived from patients with advanced melanoma, with the majority of samples from patients who progressed on standard of care checkpoint blockade (n=11, n=9 clinically resistant, table S7). 21h10 demonstrated activity against these resistant tumors, outperforming immune checkpoint blockade (ICB) antibodies (Fig. 3, D to G). These results indicate that human melanoma refractory to ICB contains T cells capable of being productively activated by 21h10.

### 21h10 induces non-neutralizing anti-drug antibodies and toxicity that can be mitigated by TNF blockade

**Fig. 4.**
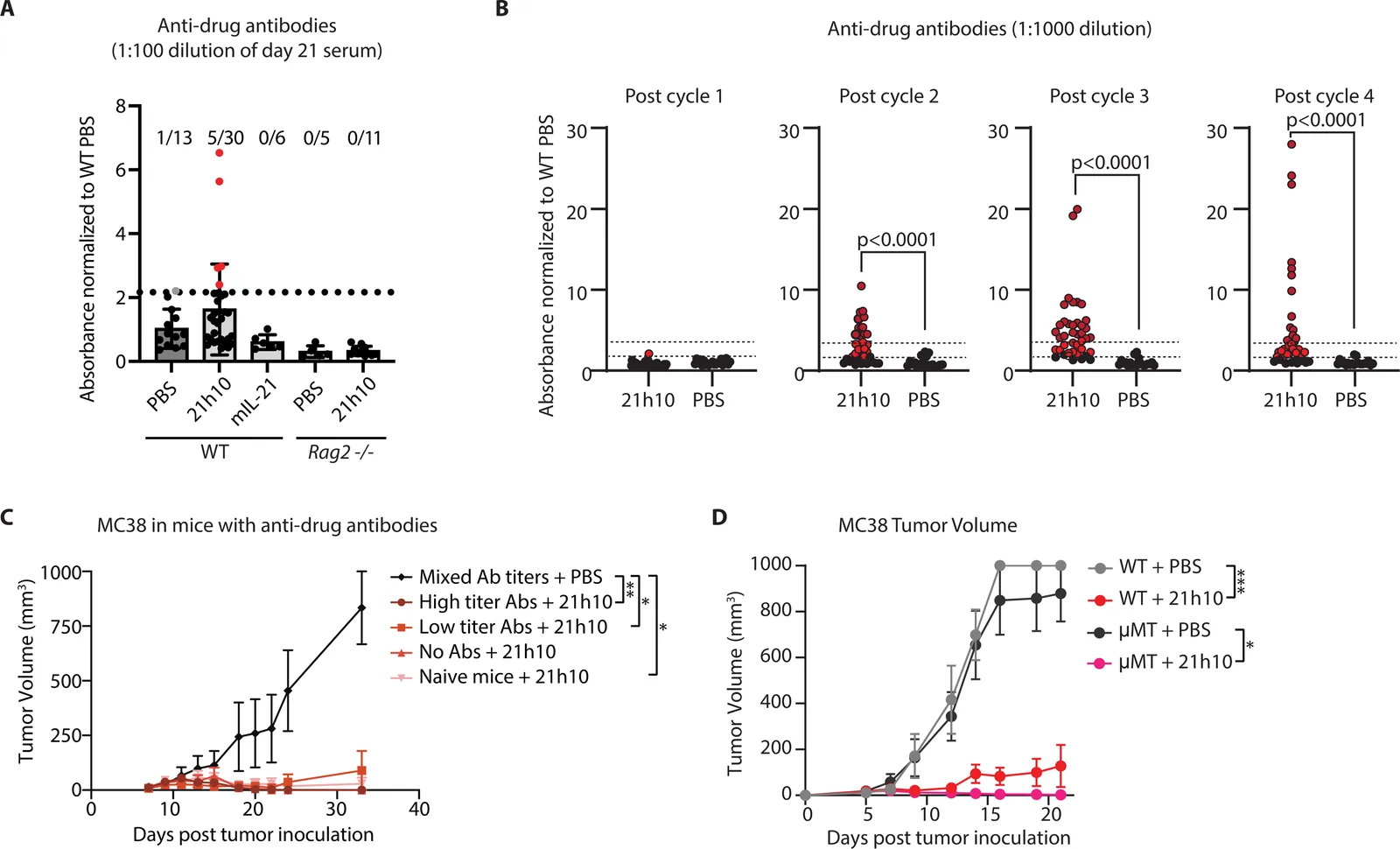
21h10 treatment elicits anti-drug antibodies in mice. (**A**) Anti-drug antibody titers were measured from day 21 sera of WT and *Rag2−/−* mice treated with PBS, 21h10 (15 μg), or mIL-21 (14.9 μg). (**B**) Anti-drug antibody titers are measured from the sera of WT mice treated with multiple dosing cycles of 21h10 or PBS. Mice received daily 21h10 (15 μg) treatment for 9 days followed by at least 9 days of rest per cycle. Dashed lines indicate 1 or 2 standard deviations above the mean of the PBS controls and were used to define low titer (1–2 SD above the mean, light red) and high titer (>2 SD above the mean, dark red) serum samples. Significance was determined using Mann Whitney test and reported when 21h10 was greater than the negative control. (**C**) Mice with various titer levels of anti-21h10 antibodies were inoculated with MC38 tumors. Starting on day 6, mice were treated with 21h10 or PBS daily for 17 days. Significance was determined using one-way ANOVA with Dunn’s multiple comparisons correction using AUC values for each mouse versus PBS group. (**D**) The antitumor activity of 21h10 in MC38 was compared in WT and μMT mice. Significance was determined using Brown-Forsythe and Welch one-way ANOVA with Dunnett’s correction using AUC values for each mouse versus PBS group.

We next characterized the immunogenicity and tolerability of 21h10 in mice. Anti-drug antibodies were detected in 5 of 30 mice treated with 21h10 in antitumor experiments, with low-titer antibodies to 21h10 detectable at a 1:100 dilution at a concentration two standard deviations above the mean of the PBS controls. No anti-21h10 antibodies were detected in mIL-21-treated or *Rag2−/−* controls (Fig. 4A), consistent with the low sequence homology between native mIL-21 and 21h10. Repeated cycles of dosing consisting of nine days of 21h10 followed by at least nine days of rest led to progressively higher incidence of anti-drug antibodies detectable at 1:1000 dilution of serum (Fig. 4B). Mice with high titer antibodies did not develop injection site reactions or anaphylaxis, and all mice regained weight between each cycle of 21h10 dosing. Importantly, anti-21h10 antibodies did not appear to neutralize 21h10 given that 21h10 retained activity in these animals upon subsequent treatment for MC38 tumors. Mice that developed none, low-, or high-titer antibodies were able to subsequently respond to 21h10 treatment in the MC38 model, showing no correlation between antitumor activity and the level of anti-21h10 antibodies generated (Fig. 4C). Furthermore, the antitumor response against MC38 was not dependent on B cells, as μMT mice which lack B cells showed responses to 21h10 that were equivalent to wild-type animals (Fig. 4D).

**Fig. 5.**
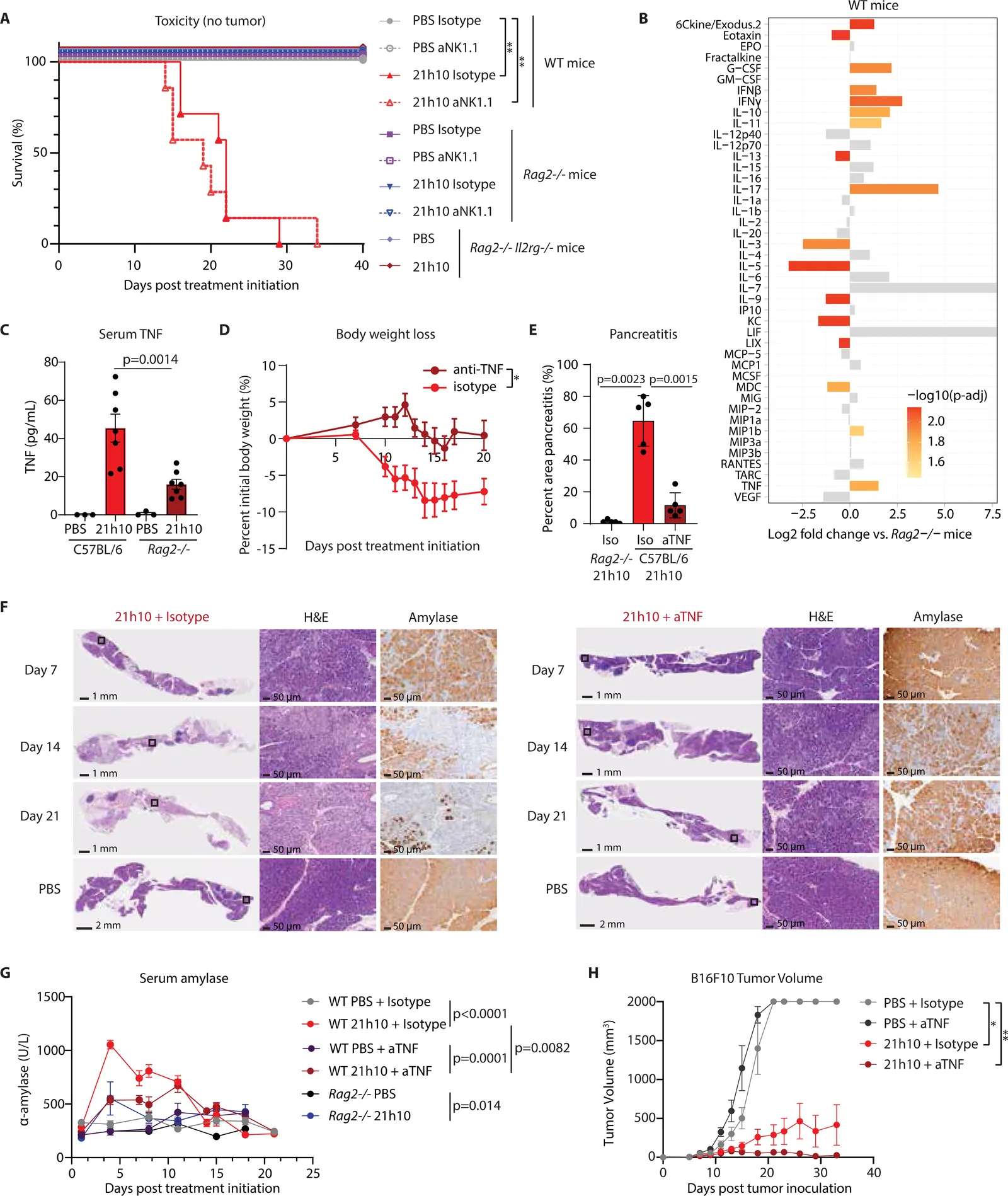
Toxicity of 21h10 is alleviated by TNF blockade. (**A**) Wild-type (WT), *Rag2−/−*, or *Rag2−/− Il2rg−/−* mice were treated with PBS or 21h10 daily and isotype or anti-NK1.1 depleting antibodies every three days. Mice were sacrificed when weight loss exceeded 20% of the initial starting weight. Significance was determined in Kaplan-Meier survival curves with Bonferroni-corrected Mantel-Cox log-rank tests. (**B**) Serum cytokine levels of various cytokines after 21h10 treatment in WT mice in comparison with *Rag2−/−* mice. Bars in gray are non-significant compared to *Rag2*−/− mice. Adjusted p-values were calculated from Wilcoxon tests with Bonferroni correction. (**C**) Serum TNF level was measured in C57BL/6 and *Rag2−/−* mice with PBS or 21h10 treatments. Significance was determined using one-way ANOVA with Šidák’s correction. (**D**) Body weight change plot in isotype- and anti-TNF antibody-treated mice with 21h10 treatment. Significance was determined using Mann-Whitney test of net AUC values for each mouse. (**E**) Percent area of pancreatitis in 21h10-treated *Rag2−/−* and C57BL/6 mice with isotype- or anti-TNF antibody treatments. Significance was determined using Brown-Forsythe and Welch ANOVA with Dunnett’s correction. (**F**) Pancreas histology of mice treated with PBS, 21h10+isotype antibody, or 21h10+anti-TNF antibody, on day 7, 14, and 21 from treatment initiation. Hematoxylin and eosin (H&E) and amylase stainings are shown on indicated days. (**G**) Serum amylase level is measured in WT mice treated with PBS+isotype-antibody, 21h10+isotype-antibody, PBS+anti-TNF antibody, or 21h10+anti-TNF antibody, and *Rag2−/−* mice treated with PBS or 21h10. Significance was determined using Brown-Forsythe and Welch ANOVA with Dunnett’s correction using AUC values for each mouse. (**H**) Antitumor activity comparison on B16F10 melanoma between PBS+isotype-antibody, 21h10+isotype-antibody, PBS+anti-TNF antibody, or 21h10+anti-TNF antibody treated groups. Data are representative of 2–3 experiments. Significance was determined using Brown-Forsythe and Welch ANOVA with Dunnett’s correction using AUC values for each mouse versus PBS+isotype group.

Although mice could tolerate 21h10 for two weeks of treatment, most animals exhibited weight loss with longer dosing. This toxicity did not require NK cells, as it was not attenuated by treatment with NK cell-depleting antibodies (Fig. 5A and fig. S12A). In contrast, *Rag2−/−* mice, which lack T and B cells, and *Rag2−/− Il2rg−/−* mice, which additionally lack NK cells and other innate lymphoid cells, were protected from 21h10 toxicity (Fig. 5A and fig. S12A), implicating adaptive immunity in 21h10 toxicity. To evaluate whether a cytokine produced by adaptive immune cells may be causing toxicity, we measured serum cytokines in 21h10-treated WT mice and *Rag2−/−* mice. Several cytokines were elevated in WT mice compared to *Rag2−/−* controls treated with 21h10 (Fig. 5, B to C), including TNF, which is known to contribute to immune-related toxicities in humans(50). When we co-administered an antibody that blocks TNF with 21h10, weight loss was mitigated, indicating that TNF plays an important role in 21h10 toxicity (Fig. 5D). Weight loss in mice can be caused by multiple factors. Histological examination of mice treated with 21h10 revealed that most organs were normal (fig. S12B). We further evaluated serum creatinine, liver transaminases, and lung histology, which appeared similar to PBS-injected control mice (fig. S13). However, the exocrine pancreas was strikingly inflamed, with more than half of the pancreas area consisting of pancreatitis after 14 days of administration of 21h10 in WT but not in *Rag2−/−* mice (Fig. 5, E to F). Co-administration of TNF blockade greatly mitigated the development of pancreatitis, as shown by prevention of acinar to ductal metaplasia and lymphocytic infiltration (Fig. 5F). By multiple criteria, including histology, immunohistochemistry for tissue amylase, and serum amylase activity over time, TNF blockade reduced pancreatitis development from 21h10, making this a reasonable strategy for toxicity prevention (Fig. 5G). In the B16F10 melanoma model, blockade of TNF did not impair the antitumor activity of 21h10, supporting successful prevention of toxicity and preservation of antitumor efficacy (Fig. 5H).

### 21h10 expands tumor cytotoxic T cells and reduces Treg cell infiltration

**Fig. 6.**
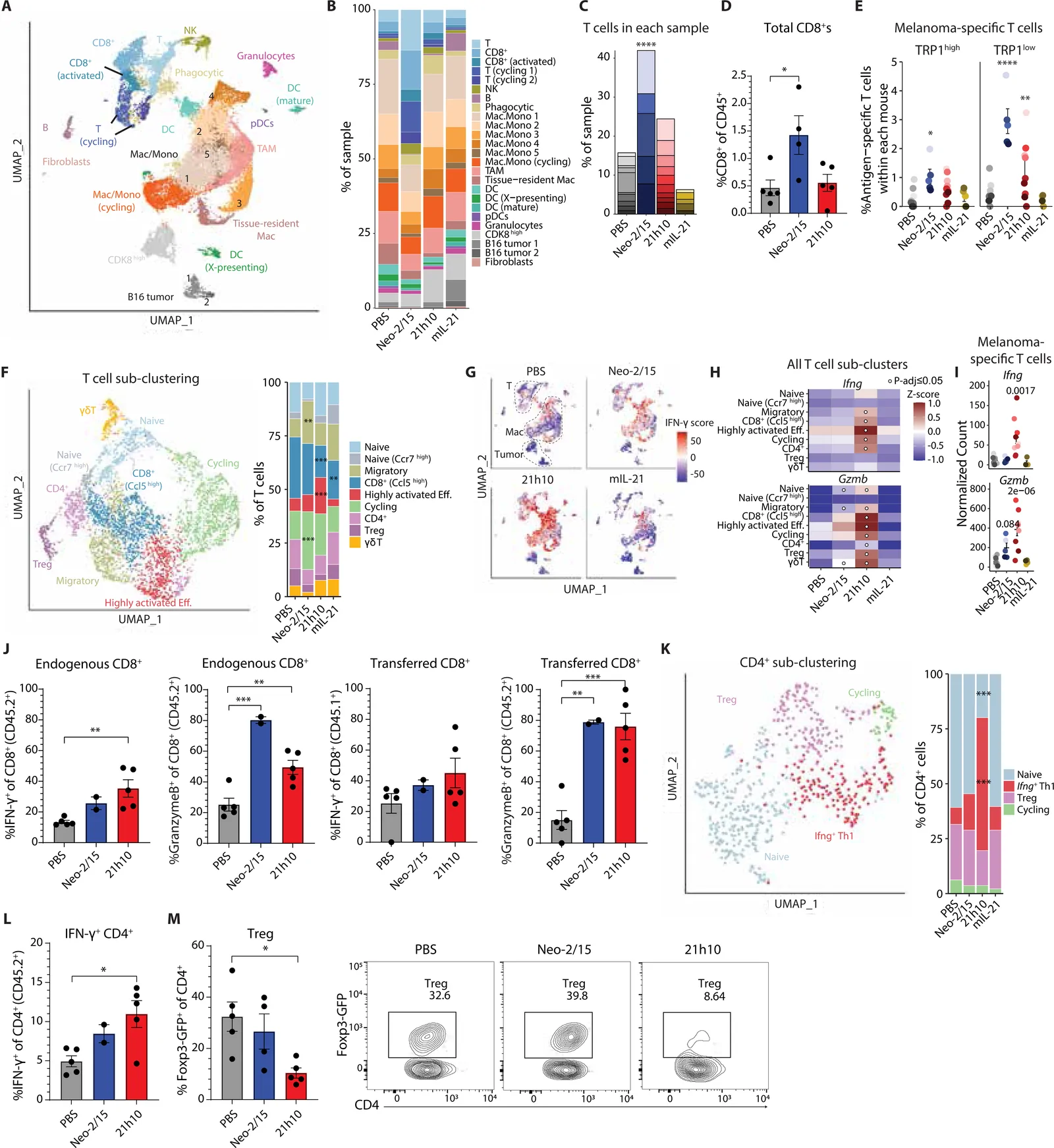
21h10 treatment results in expanded antitumor T cells with enhanced effector phenotype. (**A**) Mice were treated as in Fig. 3, A and C, but on day 15 of tumor growth, tumors were processed for scRNAseq. Ten (PBS, 21h10) or five (Neo2/15, IL-21) mice per group were collected and individual tumors were labeled with hashtag antibodies before pooling tumors from each group and sequencing. UMAP of all samples are combined. (**B**) scRNAseq cluster composition average across treatment groups. (**C**) T cells in each scRNAseq sample from (**B**). Different colors for each bar indicate individual mice from each group. Significance was determined using one-way ANOVA with Dunnett’s multiple comparisons. (**D**) Flow cytometry quantification of CD8^+^ T cell infiltration. Significance was determined using one-way ANOVA with Dunnett’s multiple comparisons. (**E**) TRP1^high^ and TRP1^low^ melanoma antigen-specific cells captured by sequencing. Significance was determined using two-way ANOVA with Dunnett’s multiple comparisons versus PBS group. (**F**) T cells were sub-clustered from all samples from (**A**). The average T cell sub-cluster composition across treatment groups is shown on the right. Significance was determined using two-way ANOVA with Dunnett’s multiple comparisons versus PBS group. (**G**) IFN-*γ*-response score total UMAP from (**A**) based on scaled and summed Hallmark IFN-*γ*-response genes for each cell. (**H**) Heatmap from pseudo-bulk differential gene expression analysis across T cell sub-clusters. Significant differences compared to PBS samples. (**I**) Similar to (**H**), but for TRP1 cells only, values from individual mice are shown. Adjusted p-values displayed vs. PBS samples. (**J**) Flow cytometry quantification of CD8^+^ IFN-*γ*/granzyme B levels in endogenous or transferred CD8^+^ TRP1^high/low^ T cells. (**K**) CD4^+^ T cell sub-clustering UMAP and cluster composition. Significance was determined using two-way ANOVA with Dunnett’s multiple comparisons versus PBS group. (**L**) Flow cytometry of IFN-*γ*^+^ CD4^+^ T cells. (**M**) Quantification and representative flow cytometry plots of Foxp3-GFP Treg cells. Significance was determined using one-way ANOVA with Dunnett’s multiple comparisons for flow cytometry quantification and scRNA-seq total T cell comparison. *DESeq2* was used to identify differentially expressed genes from scRNA-seq using the Wald test. For all dot plots with error bars: dots indicate individual mice, bars are S.E.M.

IL-21 has diverse roles within the immune system depending on the responding cells and the context in which it is produced(10). To understand the mechanism of action of 21h10, we performed single-cell RNA-sequencing (scRNAseq) on CD45^+^ enriched cells from B16F10 melanoma tumor 14 days after tumor inoculation, comparing mice treated with PBS, Neo-2/15, mIL-21, and 21h10 in addition to adoptively transferred TRP1^high/low^ CD8^+^ T cells. Using unbiased clustering, we identified populations of T cells, multiple monocyte/macrophage populations, dendritic cells, and smaller clusters of B cells, NK cells, granulocytes, melanoma cells, and fibroblasts (Fig. 6A and fig. S14A) in each treatment group (Fig. 6B). All T cell clusters were expanded in mice treated with Neo-2/15 (Fig. 6, B to C), consistent with the known effects of IL-2 and IL-2 mimics on T cell proliferation and survival(51). Conversely, 21h10, mIL-21, and PBS had modest effects on the relative proportion of T cells within the tumor microenvironment (Fig. 6, B to C). The T cell expansion observed with Neo-2/15 was primarily among CD8^+^ T cells, consistent with preferential expansion of CD8^+^ T cells compared to CD4^+^ T cells by IL-2 (52) (Fig. 6D and fig. S15A). Like total T cells, TRP1-specific T cells, particularly low-affinity TRP1^low^ cells, expanded with Neo-2/15 (Fig. 6E). Although 21h10 treatment did not expand total CD8^+^ T cells, tumor-specific low-affinity TRP1^low^ cells accumulated in 21h10-treated tumors (Fig. 6E), consistent with studies showing that low-affinity T cell expansion can be greater than that of their high-affinity counterparts(53). Native mIL-21 did not expand TRP1^high^ or TRP1^low^ T cells (Fig. 6E).

The high antitumor activity of 21h10 is striking, given the modest effect on T cell expansion. T cell sub-clustering identified two naïve T cell populations expressing *Tcf7*, a migratory cluster (*Itga4, Themis*), activated CD8^+^ T cells (*Ccl5, Gzmk*), highly activated CD8^+^ T cells expressing multiple immune checkpoint receptors (*Havcr2, Lag3*) and effector molecules (*Gzmb, Ifng*), a proliferative cluster (*Mki67, Birc5*) and CD4^+^ T cells (*Cd4, Cd40lg*), regulatory T (Treg) cells (*Foxp3, Ctla4*), and *γ*𝛿 T cell clusters (Fig. 6F and fig. S14B). T cell expansion by Neo-2/15 mainly occurred in the migratory and cycling clusters, but 21h10 had a greater effect on highly activated CD8^+^ T cells. No significant increases in specific T cell populations were observed with mIL-21. Neo-2/15 primarily affected pathways involved in cell growth and proliferation, while mIL-21 showed a similar, albeit weaker effect compared to PBS. 21h10 treatment was associated with IFN-*γ*/IFN-𝛼 response genes and growth pathways (fig. S14C) in all immune populations identified, including the myeloid and T cell clusters (Fig. 6G). 21h10 expanded the most activated CD8^+^ T cells and increased *Ifng* and *Gzmb* gene expression in most T cell subclusters (Fig. 6H), including the transferred melanoma-specific CD8^+^ T cells (Fig. 6I). Correspondingly, IFN-*γ* and granzyme B levels were increased in both endogenous and transferred T cells from 21h10-treated animals, as compared to PBS controls (Fig. 6J and fig. S15B). Increases in *Gzmb* and *Prf1* were also observed in NK cells from mice treated with 21h10, although total NK cell frequencies were similar between PBS and 21h10 groups (fig. S14, D to E).

There were fewer tumor-infiltrating CD4^+^ T cells than CD8^+^ T cells, as shown by both flow cytometry and scRNAseq (Fig. 6, C to D). We subclustered the CD4^+^ T cells in our scRNAseq data and identified four populations: naïve CD4^+^, cycling CD4^+^ T cells, Treg cells, and Th1 cells expressing *Ifng* mRNA (Fig. 6K and fig. S14F). The *Ifng*^+^ Th1 cells preferentially expanded in response to 21h10 as compared to the other treatments, and had higher levels of IL-21R, analogous to what we observed with CD8^+^ T cells (Fig. 6, K to L, and figs. S15C and S16). Our scRNAseq analysis also suggested that 21h10 decreased Treg cells, which we confirmed by flow cytometry using Foxp3-GFP reporter mice (Fig. 6M). Thus, 21h10 treatment robustly activates STAT1 and STAT3 in T cells in the tumor microenvironment, and this is accompanied by a shift toward highly activated, IFN-*γ*- and granzyme-B-producing, anti-melanoma CD8^+^ T cells as well as a relative increase in Th1 cells and decrease in Treg cells.

## DISCUSSION

Cytokine-based therapies for cancer have historically had limited efficacy and high toxicity(54–56). Here, we show that a computationally-designed mimic of IL-21 with improved stability can have potent antitumor activity, both as a monotherapy and in combination with adoptive T cell therapies. 21h10 expanded highly activated effector CD8^+^ T cells in the tumor microenvironment, with the effect most pronounced for CD8^+^ T cells with known tumor reactivity. In contrast, the IL-2/IL-15 mimic Neo-2/15, expanded multiple T cell populations without a preferential effect on the differentiation of effector cells, providing a possible mechanistic explanation for the difference in antitumor activity between these two cytokine mimics. Antitumor T cell responses can display a range of receptor affinities, with low-affinity responses typically seen when a self-antigen is targeted(57, 58). Using single-cell RNA sequencing, we found that CD8^+^ T cells recognizing the melanoma self-antigen TRP1 with a range of affinities were expanded by 21h10, but strikingly, low-affinity TRP1-specific T cells were preferentially increased, indicating that 21h10 selectively amplified lower affinity antitumor T cells. By contrast, PD-1 blockade immunotherapies appear to act on high-affinity neoantigen-reactive clonotypes(59), and immunotherapies that can augment a broader spectrum of TCR affinities are currently lacking. Low-affinity T cells often fail to receive a signal strong enough to overcome the TCR signaling threshold(60), and thus are frequently unable to contribute to antitumor immunity(45). By expanding the TRP1^low^ population, 21h10 broadens the repertoire of the antitumor immune response, potentially explaining the efficacy observed in *ex vivo* cultures of immunotherapy resistant human melanoma. 21h10 also decreased the Treg cell population while inducing an effector phenotype on CD8^+^ T cells. Although IL-21 has important roles in B cell differentiation, class switching, and NK cell activation, neither B nor NK cells were significantly altered in our *in vivo* tumor models.

Although we chose 21h10 for its ability to mimic IL-21 signaling *in vitro*, the resulting *de novo* protein showed substantially superior performance *in vivo*. We showed that 21h10 has increased serum stability compared to native mIL-21, leading to sustained potency *in vivo*. These properties increase the duration of signaling *in vivo*, leading to enhanced phenotypic changes and, ultimately, improved antitumor responses. These improvements highlight the robustness of *de novo* protein design approaches for developing therapeutic candidates with improved properties. The full human/murine cross-reactivity and engineerability of 21h10 should make it straightforward to generate targeted and conditionally active versions(33) to mitigate toxicity and target activity to the tumor microenvironment.

Limitations to clinical development of 21h10 include toxicity, a concern for any systemically delivered cytokine, and immunogenicity, a concern for any *de novo* protein intended to be administered more than once. We used 21h10 as an example to address both of these broader issues. Toxicity appeared to be associated with a specific organ (the pancreas) and mediated by TNF, which could be prevented by TNF blockade, a therapy commonly used for treatment of immune-related adverse events(61). Use of a two-drug regimen could be clinically challenging; thus, further design of 21h10 might include fusion with targeting domains to avoid accumulation in the pancreas, or fusion with a designed protein that blocks TNF signaling. Immunization with 21h10 led to formation of anti-drug antibodies, but they were not neutralizing and did not attenuate the therapeutic efficacy of 21h10 in our models. Similarly, anti-drug antibodies to the designed cytokine Neo-2/15 were rare and did not attenuate efficacy, as we previously reported(32). We hypothesize that either the immunodominant epitopes for these two examples of cytokine mimics are not located in the receptor-binding interface or that the high affinity of 21h10 for its receptor favors binding even in the presence of a bound antibody. Although the placement of the immunodominant epitopes or affinity of the elicited antibodies appear to have fortuitously worked in our favor, efforts to intentionally redirect anti-drug antibodies away from receptor-binding interfaces may be important in the design of future cytokine mimics.

In summary, we have developed a potent IL-21 mimic with activity on both human and mouse cells that exhibits robust antitumor activity in multiple tumor models. 21h10 has antitumor activity as a monotherapy, synergizes with adoptive cell therapy in B16F10 melanoma model, and is curative in a highly immunogenic MC38 adenocarcinoma model. 21h10 causes pancreatitis in mice, but it is mitigated by TNF blockade. Activity of 21h10 in human refractory melanoma PDOTS and its superiority on clinically approved ICB treatments suggest the potential for clinical translation to treat tumors unresponsive to checkpoint blockade and other existing therapies.

## MATERIALS AND METHODS

### Study design

This study aimed to evaluate the stability, signaling activity, immunogenicity, toxicity, and antitumor efficacy of 21h10, a computationally designed *de novo* mimic of IL-21, in both murine models and *ex vivo* human tumor explants. The study involved *in vitro* biochemical and signaling assays, murine tumor models (MC38 and B16F10), adoptive T cell transfer, CAR-T cells, patient-derived organotypic tumor spheroids (PDOTS), and single-cell RNA sequencing to characterize immune responses. Sample sizes were determined based on prior experiments and effect size expectations in similar models, though formal power calculations were not performed. No animals or data points were excluded unless pre-established technical failure criteria (e.g., injection errors, lack of tumor engraftment) were met.

Randomization was performed for animal studies where mice were randomly assigned to treatment groups once tumors reached comparable sizes. Human tumor explants were allocated based on tissue availability and the researchers were not blinded to the treatment conditions. Blinding was implemented for outcome assessments where feasible, including for tumor measurements, immunohistochemistry, and histological scoring of toxicity. Experiments were conducted in multiple independent biological replicates: tumor treatment studies were repeated at least twice with 5–10 animals per group, and PDOTS experiments were performed using 11 patient samples, with two or three biological replicates for each. Flow cytometry, RNA-seq, and scRNA-seq experiments were conducted with independent replicates from separate tissue preparations or donors. Specific details on replicates and statistical methods are provided in the respective figure legends and Data file S1.

### Computational design of *de novo* IL-21 mimic

The crystal structure of hIL-21 with hIL-21R (PDB: 3TGX) was used to design the mimics of native IL-21. The *PyRosetta* script with PDBInfoLabel metadata implementation generated IL-21-like scaffolds(32) in code S1. The residues from the human IL-21 are designated to be fixed during scaffold generation and interface residue design in code S2. The non-fixed residues are designed using *Rosetta* FastDesign and relaxed using *Rosetta* FastRelax with the ‘beta_nov16’ score function. 185 designs were filtered with *Rosetta* score metrics: packstat>0.6, score_per_residue<−2.3, and sspred>0.8.

### Crystallization, data collection, and refinement

IL-21R and *γ*_c_ were expressed, purified, and deglycosylated as previously described(37). *γ*_c_ was mixed with 21h10/IL-21R complex at an equimolar ratio, concentrated to 10 mg/mL, and treated with carboxypeptidase A and B. Crystals of the 21h10 complex were grown by sitting drop vapor diffusion in 0.2 M potassium thiocyanate, 0.1M Bis-Tris propane pH 6.5, 20% w/v PEG-3350, and crystals were harvested as previously described. Diffraction data were collected at Stanford Synchrotron Radiation Laboratory beamline 12–2. Data were indexed, integrated, and scaled to 2.3Å resolution using XDS(62). Due to anisotropy in the crystal diffraction, a pseudoellipsoidal diffraction limit was applied using Staraniso(63). The protein structure was solved using an approach similar to the solution of the native ternary structure(37), using molecular replacement in Phaser(64)) based on models derived from the IL-21/IL-21R complex (PDB: 3TGX)(35) and a *γ*_c_ complex (PDB: 7S2R)(65). The solution yielded an asymmetric unit containing four copies of 21h10, four copies of IL-21R, and two copies of *γ*_c_. Coot(66) and Phenix(67) were used to rebuild and refine the structure, and refinement was performed with noncrystallographic symmetry restraints. Crystallography data collection and refinement statistics are presented in table S3. The final 21h10/IL-21/IL-21R structure has been deposited in the RCSB protein data bank with accession code PDB ID: 9E2T. SBGrid(68) was used to install and configure all crystallographic software. Analysis of interfaces was conducted using PISA(69) (table S4). Structure-related figures were generated using ChimeraX(70) (UCSF).

### STAT phosphorylation assay in T cells

All experiments involving mice at NHLBI were performed using protocols approved by the NHLBI Animal Care and Use Committee and followed National Institutes of Health (NIH) guidelines for the use of animals in intramural research. Purified murine CD4^+^ and CD8^+^ T cells were pre-activated with plate-bound anti-mouse CD3 (2 μg/mL; clone 145–2C11, BioXCell Cat# BE0001–1) and soluble anti-mouse CD28 (1 μg/mL; clone 37.51, BioXCell Cat# BE0015–1) and cultured in complete RPMI media for 48 hours at 37°C. Human CD4^+^ and CD8^+^ T cells were isolated from the buffy coats of healthy human volunteers obtained from NIH Blood Bank. Purified human CD4^+^ and CD8^+^ T cells were activated by plate-bound anti-human CD3 (2 μg/mL; clone UCHT1 (Leu-4) (T3), BioXCell Cat# BE0231) and soluble anti-human CD28 (1 μg/mL; clone 9.3, BioXCell Cat# BE0248) and cultured in complete RPMI medium for 48 hours at 37°C. For B cells, see Supplementary Materials and Methods for details.

After 48 hours of culture, the pre-activated cells were rested overnight in a complete medium and restimulated with native IL-21 and IL-21 mimics at different concentrations (from 10 fM to 1 μM) for 20 minutes, respectively. Cells were then fixed, permeabilized, and stained with AF488-phosphoSTAT1 (1:100, BD Biosciences Cat# 612596) and AF647-phosphoSTAT3 (1:100, BD Biosciences Cat# 651008) for flow cytometry. Cells were analyzed using a BD LSR Fortessa X-20 Cell Analyzer flow cytometer (BD Biosciences).

### RNA-seq analysis

The libraries were indexed using barcodes and sequenced on an Illumina NovaSeq platform with SP 100-cycle kits, achieving a sequencing depth of over 45 million reads per sample. Sequenced reads were obtained with the Illumina CASAVA pipeline and mapped to the mouse genome (mm10/GRCm38) using TopHat 2.0.11. Raw counts that fell on exons of each gene were calculated and normalized by using RPKM (Reads Per Kilobase per Million mapped reads). Differentially expressed genes were identified with the R Bioconductor package “edgeR”, and expression heat maps were generated with the R package “pheatmap”.

### Syngeneic murine tumor model experiments

All animal protocols were approved by the Dana-Farber Cancer Institute Committee on Animal Care (Protocol 14–019 and 14–037) and are in compliance with the NIH/NCI ethical guidelines for tumor-bearing animals. On day 0, 6–8 week-old female C57BL/6J mice (JAX Cat# 000664) were inoculated with 80–90% confluent tumor cells (cell line is indicated per experiment). Starting on day 3–5, mice were treated daily with the listed test items, and 21h10 was injected daily following each regimen indicated per experiment. Mice were monitored for survival, weight change, and symptoms of toxicity, including pallor, noticeable weight loss, and fatigue. Mice were euthanized if they lost 20% of body weight or their tumors ulcerated or reached 2,000 mm^3^ in volume (measured with a ruler, volume calculated by multiplication of length, height, and width), which is the maximal permitted tumor size for these studies. For all *in vivo* mouse tumor experiments, mice were randomized after transfer of TRP1 cells and after inoculation of tumors, prior to starting drug treatment. Mice that did not have palpable tumors at the time of first drug dosing were excluded from the experiment. Mice whose tumors ulcerated prior to reaching 500mm^3^ were also excluded. Investigators were not blinded. Sample size determination was not performed. Experiments were performed once unless otherwise stated.

### Patient-derived organotypic tumor spheroid (PDOTS) preparation and microfluidic device culture

Organotypic spheroids were generated from samples of patients with melanoma at Massachusetts General Hospital. Samples were collected and analyzed according to Dana-Farber/Harvard Cancer Center Institutional Review Board (IRB)-approved protocols (IRB protocol: 11–181). Written informed consent was obtained from all subjects. PDOTS were generated as previously described(48, 71, 72). Tumors were minced finely with scalples. Minced tumors were resuspended in full DMEM. Cells were filtered through 100-μm cell strainer, followed by 40-μm cell strainer to obtain three size-based fractions: S1 (>100 μm), S2 (40–100 μm), and S3 (<40 μm). The S2 and S3 fractions were washed off the filters with fresh complete medium and rested in ultra-low attachment plates (Corning, 3471) in the incubator until device loading. The S1 fraction was washed off using a complete medium supplemented with 100 U/mL collagenase type IV (Life Technologies, 17104019) and 15 mM HEPES (Gibco, 1560–080), followed by incubation at 37 °C for 15–30 minutes. Collagen digestion was quenched with an equal volume of medium, and the suspension was subsequently re-filtered to S1, S2 and S3 fraction. S1 was washed off this time with complete media Finally, the S2 fraction was pelleted and embedded in type I rat tail collagen (Corning, 354236) at 2.5 mg/mL, prepared with 10× PBS containing phenol red (Sigma-Aldrich, 114537–5g). PDOTS were treated with ICB (250 μg/mL pembrolizumab or 240 μg/mL nivolumab + 80 μg/mL relatlimab (opdualag, obtained from MGH pharmacy waste)) human recombinant IL-21 at 1 μM, or left untreated.

### PDOTS viability assessment

PDOTS staining and viability analysis was done as previously described(48, 71). Image analysis was performed using the NIS-Elements AR software. Live/dead quantification was determined by measuring the total cell area of acridine orange (live cells), propidium iodide (PI; dead cells), and Hoechst (total cells). Live cell counts were defined as the total area of the acridine orange channel or as the Hoechst (total cell counts) minus the PI (dead) area. Percent change and log2 fold change (L2FC) values were calculated from the live cells in each condition relative to the control.

### Single-cell RNA-sequencing preparation

Tumors were collected from mice as described previously(73). For each tumor from a treatment group, a different TotalSeqC hashing antibody (mouse Hashtags 1–5, Biolegend) was incubated with 2 μL Ab/million cells in PBS (with 2% FBS) for 20 minutes at 4°C. Approximately 4,000 cells/tumor were pooled across each treatment group (five hashtags/treatment group) and loaded onto a 10X Chromium Controller with a Single Cell K Chip (PN-2000182) using the Chromium Next GEM Single Cell 5ʹ GEM Kit v2 reagents and beads (PN-1000244 and PN-1000264). Samples were sequenced at a depth of 50,000 read pairs/cell.

### Single-cell RNA-sequencing pre-processing

The *Mus musculus* genome fasta and General Transfer Format (GTF) annotation files were downloaded from the Ensembl database (release 108). Cellranger (v7.0.0) ‘mkgtf’ was used to filter the GTF file using the defaults from 10x Genomics. A custom reference was generated by first appending the sequences for the TRP1^high/low^ rearranged α/β TCR chains(45) to the *M. musculus* fasta file, adding TRP1 TCR annotations to the filtered GTF file, and finally using these two new files as inputs for Cellranger ‘mkref’ to build the reference. Cellranger ‘count’ was used to align reads to this new reference (Gene Expression) and hashtag antibody sequences (Antibody Capture). The unfiltered Cellranger count matrices were imported into R using the *Seurat* (v4.3.0) package. The *DropletUtils* (v1.18.1) package ‘emptyDrops’ function was used to exclude potential empty droplets from the count matrix(74) using a cutoff of FDR < 0.01. Hashtag reads were used to demultiplex samples with the Seurat ‘HTODemux’ function, using a kmeans function with “nstarts = 30” and a 99% positive quantile threshold.

### Single-cell RNA-sequencing clustering

Each filtered, demultiplexed sample was log normalized, and 2,000 variable features were identified and used to find 3,000 integration features to combine samples to minimize batch effects(75). The newly integrated dataset was scaled, and a principal component analysis (PCA) was performed, from which the top 25 PCs were used for uniform manifold approximation and projection (UMAP) generation. From the 25 PCs, a shared nearest neighbor (SNN) graph was constructed (k = 20 neighbors), and clusters were classified by the Louvain algorithm. The ‘FindAllMarkers’ function was used to find the top cluster-defining genes, and these gene lists were used to assign cell types.

### Differential gene expression analysis

Across each T cell sub-cluster, we performed a pseudo-bulk method of differential gene expression analysis(76, 77). Each mouse within a treatment group was treated as a replicate, and count matrices were summed across genes for each sub-cluster per mouse. Using the *DESeq2* (v1.38.3) package(78) and pseudo-bulk gene expression values, differentially expressed genes were identified across treatment groups and sub-clusters. For gene changes in TRP1-specific T cells, cells classified as either TRP1^high^ or TRP1^low^ cells were subset and counts across each gene were summed for each mouse for pseudo-bulk analysis using *DESeq2*. For Gene Set Enrichment Analysis (GSEA), fold changes within the T cell subset object were calculated with the *Seurat* ‘FoldChange’ function, comparing each specified treatment group. Fold changes were used with the *fgsea* (v1.24.0) package along with the *Molecular Signatures* (v7.2) Hallmark gene signatures(79) for GSEA.

### Statistical analysis

Statistical comparisons were made using GraphPad Prism 10 (GraphPad Software) or R (v4.2.2) and details reported in Data file S1. Parametric comparisons of normally distributed values were performed using one-way or two-way analysis of variance (ANOVA) with appropriate corrections for multiple comparisons, including Tukey’s, Dunnett’s, and Šidák corrections, as specified. In cases of unequal variances, Brown-Forsythe and Welch ANOVA tests were used with Dunnett’s multiple comparisons correction. For survival analyses, significance was determined using Bonferroni-adjusted Mantel-Cox log-rank tests. Where applicable, Mann-Whitney U tests were used for nonparametric unpaired comparisons, and Wilcoxon signed-rank tests with Bonferroni correction were applied to paired data. Area under the curve (AUC) values per mouse were used in some group comparisons. For gene expression and enrichment analyses, DESeq2 Wald tests and the fgsea R package were employed using an adjusted p-value cutoff of 0.05. Z-scores were computed by subtracting the row mean from each value and dividing by the row standard deviation. All statistical tests were two-tailed, and adjustments for multiple comparisons were made where appropriate. All error bars are S.E.M. Data were considered significant when p ≤ 0.05; *p < 0.05, **p < 0.01, ***p < 0.001, ****p < 0.0001 (or p-values displayed).

## Supplementary Material

SM1 Data file S1. Raw data

SM2 Data file S2. Uncropped immunoblots

SM3 Data file S3. CAR construct

Supplementary Materials Fig. S1. Design, screening, and optimization of IL-21 mimic.
Fig. S2. Electron density of 21h10.
Fig. S3. STAT signaling of CD4+ and B cells and PCA analyses and gene expression comparison at higher concentrations of cytokines.
Fig. S4. Proliferation, differentiation, and expression of IL-21R and granzyme B of CD8+ T cells in the presence of mIL-21 or 21h10.
Fig. S5. Antagonist, 21AT36, binds to IL-21R but does not signal through STAT.
Fig. S6. 21h10 shows antitumor activity against MC38 adenocarcinoma comparable to anti-PD1 and requires signaling through the IL-21 receptor.
Fig. S7. Survival and individual tumor growth curves from Fig. 2H.
Fig. S8. 21h10 shows enhanced serum stability than mIL-21.
Fig. S9. 21h10 enhances cytotoxicity of CAR-T cells and increases their serial tumor-killing capability against MC38 adenocarcinoma.
Fig. S10. Survival and individual tumor growth curves from Fig. 2H and Fig. 3.
Fig. S11. 21h10 improves antibody therapy and ex vivo-primed T cell therapy.
Fig. S12. Toxicity profiling of 21h10 treatment.
Fig. S13. Pancreas, liver toxicity profiling and lung histology with 21h10 treatment.
Fig. S14. Cluster-defining genes for single-cell RNA-sequencing.
Fig. S15. Representative flow cytometry plots.
Fig. S16. Il21r expression across clusters.
Table S1. Amino acid sequences of IL-21 mimics.
Table S2. KD values of biolayer interferometry (BLI) assays.
Table S3. Crystallography data collection and refinement statistics.
Table S4. Key contacts in the 21h10 receptor complex.
Table S5. Mean EC50 values from cross-reactivity cell signaling assays.
Table S6. Mean EC50 values of cell signaling assays.
Table S7. Clinical, demographic, and molecular characteristics of patient samples analyzed in the ex vivo PDOTS experiment.
Code S1. Parameters for scaffold generation.
Code S2. PDBInfoLabel for scaffold generation and interface design.

MDAR Checklist MDAR Reproducibility Checklist

## Data Availability

All data and information needed to support the paper are present in the manuscript or the Supplementary Materials. The crystal structure has been deposited in the RCSB protein data bank with accession code PDB ID: 9E2T. RNA sequencing has been deposited in the Gene Expression Omnibus (GEO) under the accession number GSE256289. No customized code was generated for the bulk RNA seq. All data were analyzed using existing and built-in R packages. Single-cell RNA sequencing has been deposited in the Gene Expression Omnibus (GEO) under the accession number GSE240834, with its code deposited in both Zenodo, DOI: 10.5281/zenodo.15690716 (https://zenodo.org/records/15690716), and GitHub (https://github.com/mjlwalsh/21h10_scRNAseq). Tabulated data underlying the figures is provided in Data file S1. Uncropped immunoblots are provided in Data file S2. All other data needed to support the conclusions of the paper are present in the main text or the Supplementary Materials.
