# Supplementary material for: Design of a potent interleukin-21 mimic for cancer immunotherapy
Source: Sci Immunol. Author manuscript; Available in PMC 2026 Jan 6. (PMC12768534; doi:10.1126/sciimmunol.adx1582)

Uncropped Immunoblots

Fig. 2F (pSTAT1, 3, 5)

pSTAT1

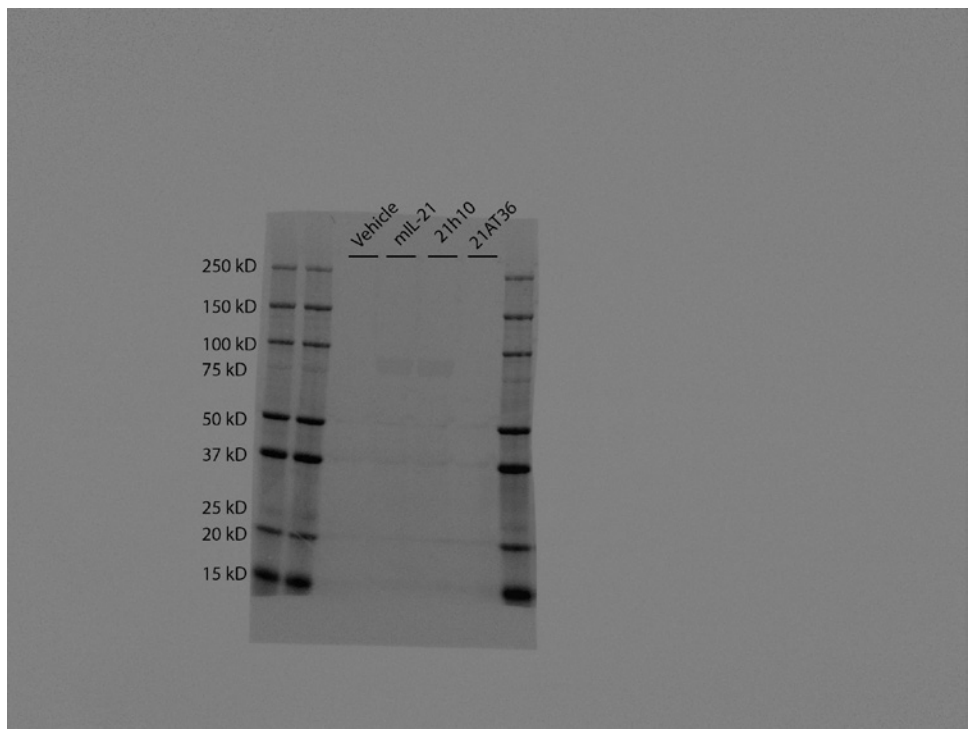

pSTAT3

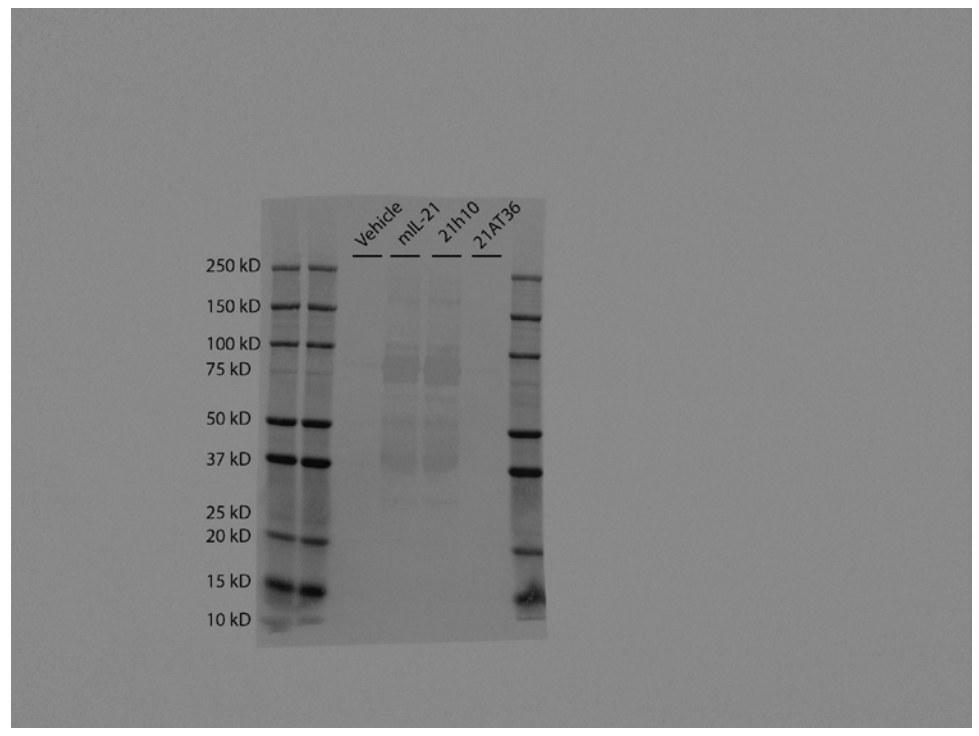

pSTAT5

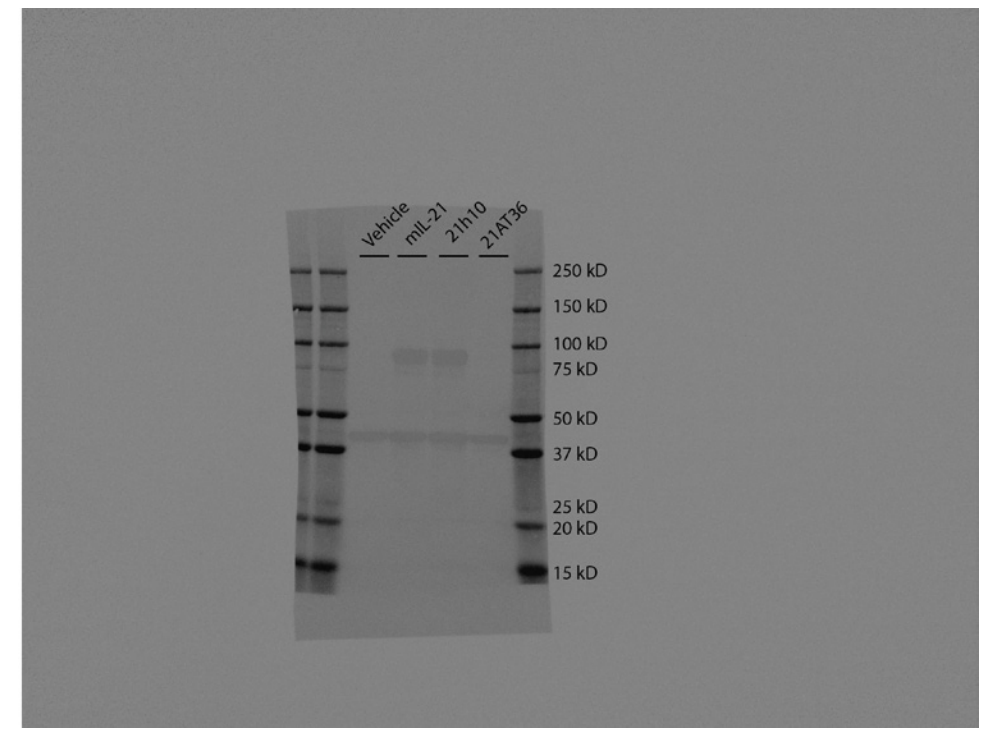

STAT1

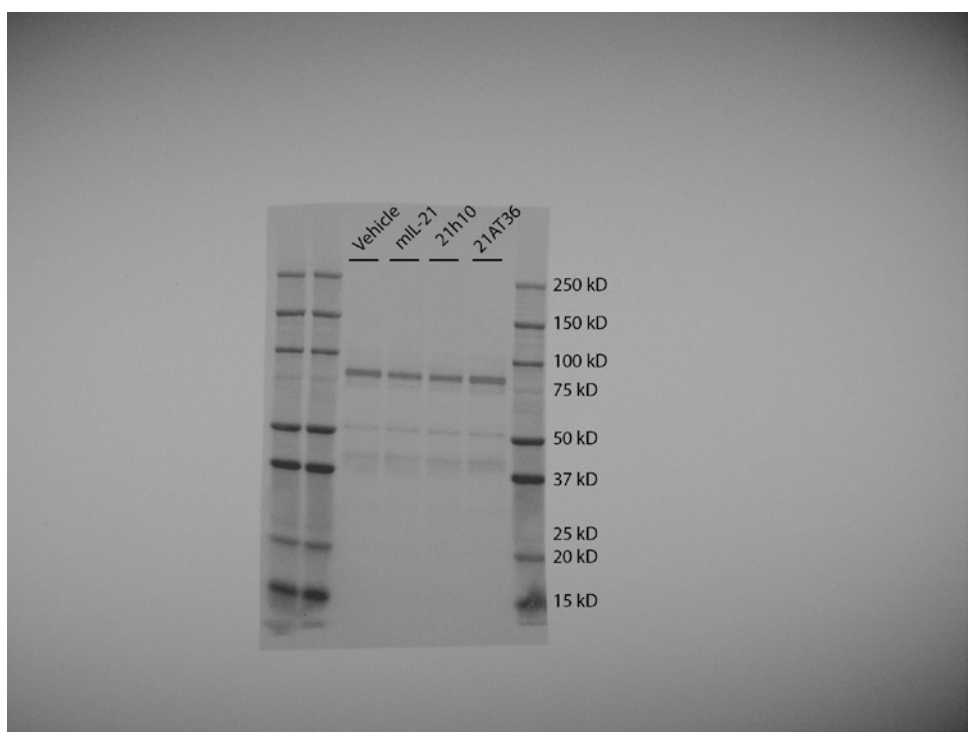

STAT3

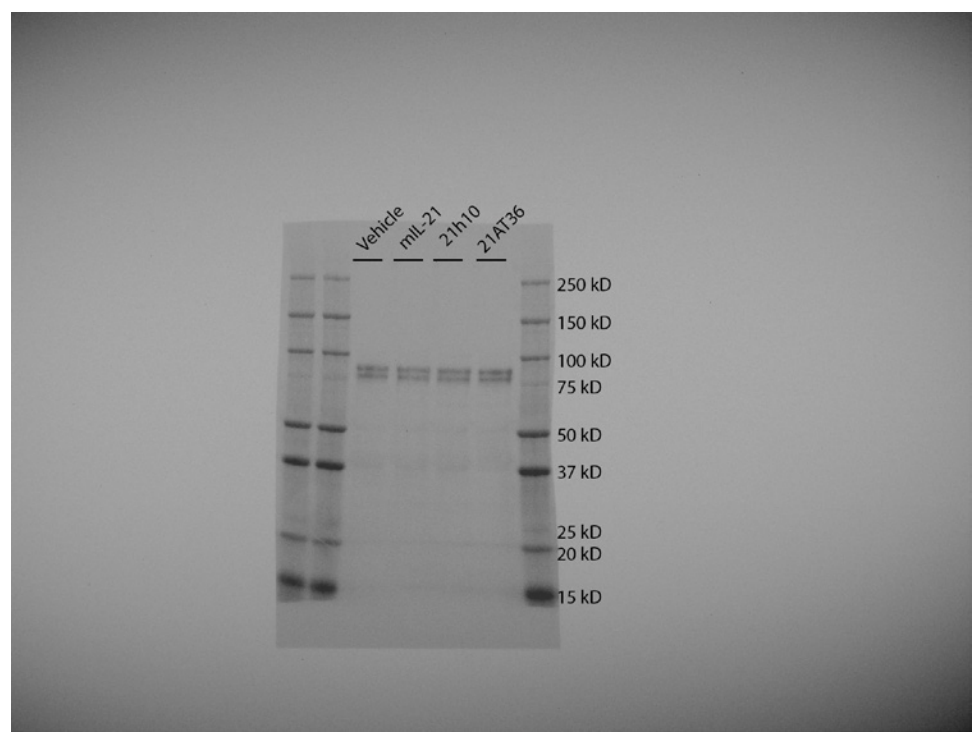

STAT5

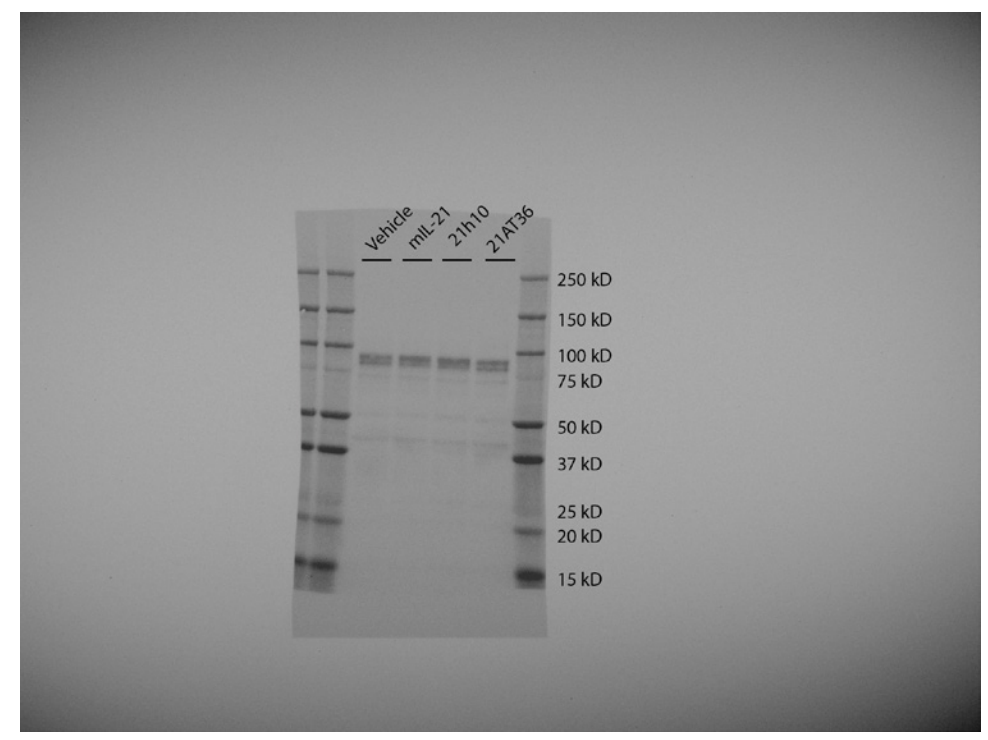

Fig. 2I (21h10 vs. mIL-21 pSTAT3)

pSTAT3 (1h, 6h)

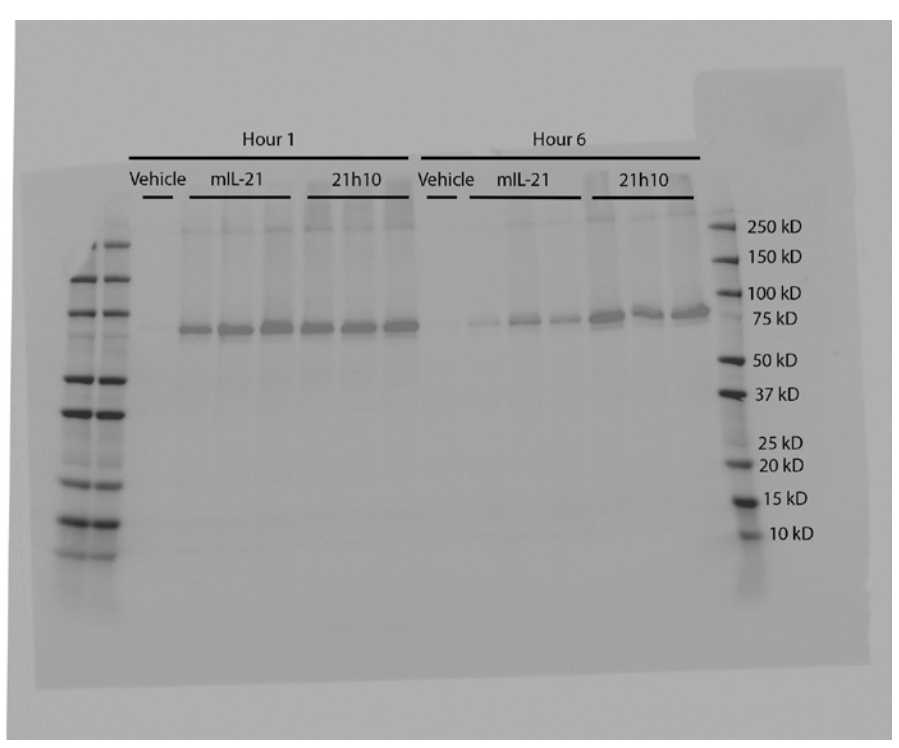

pSTAT3 (9h)

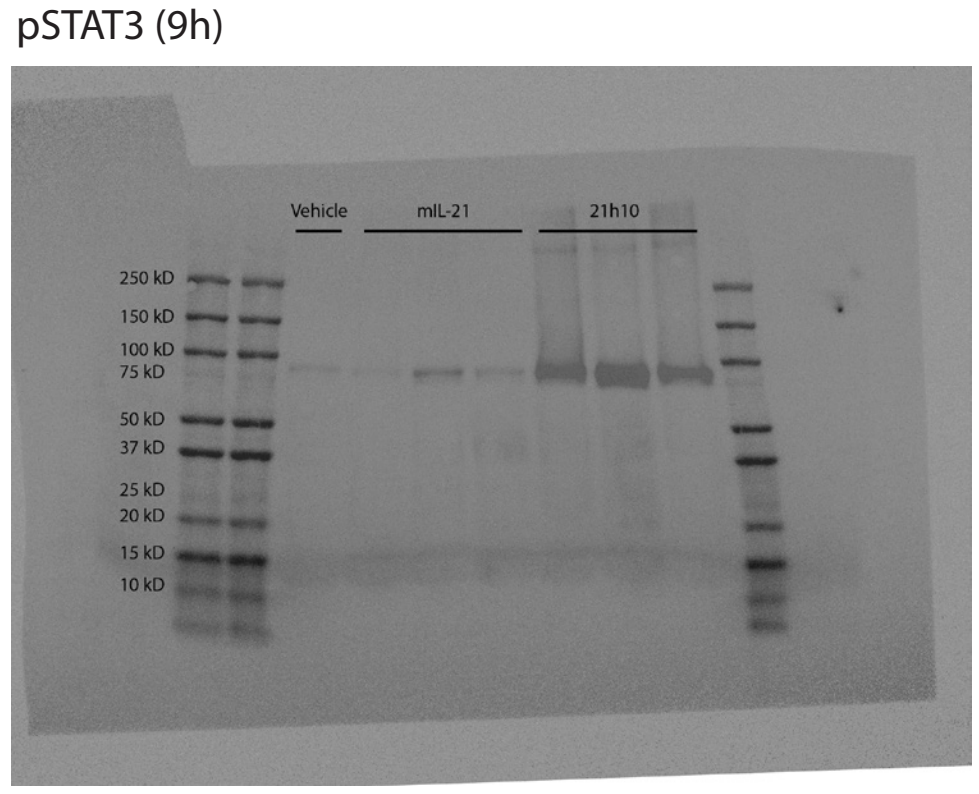

pSTAT3 (24h)

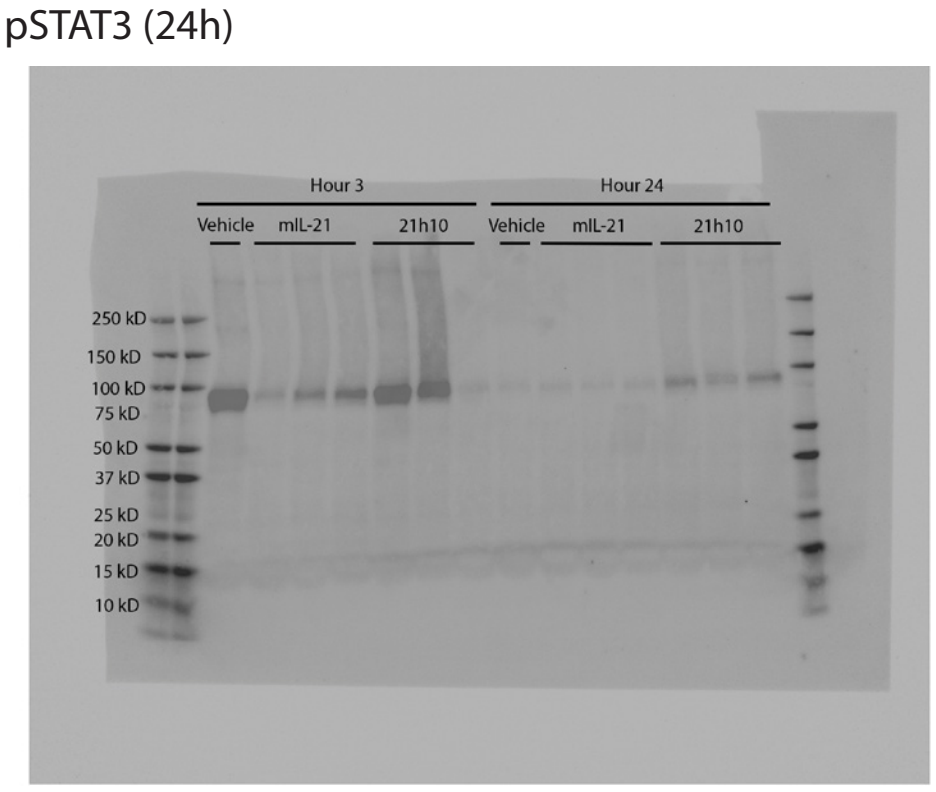

STAT3 (1h, 6h)

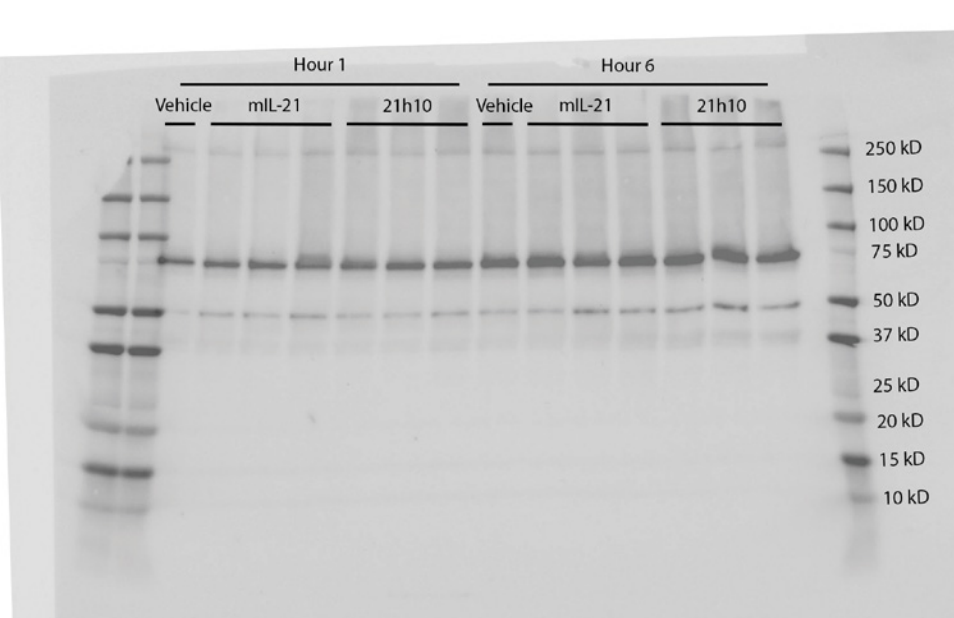

STAT3 (9h)

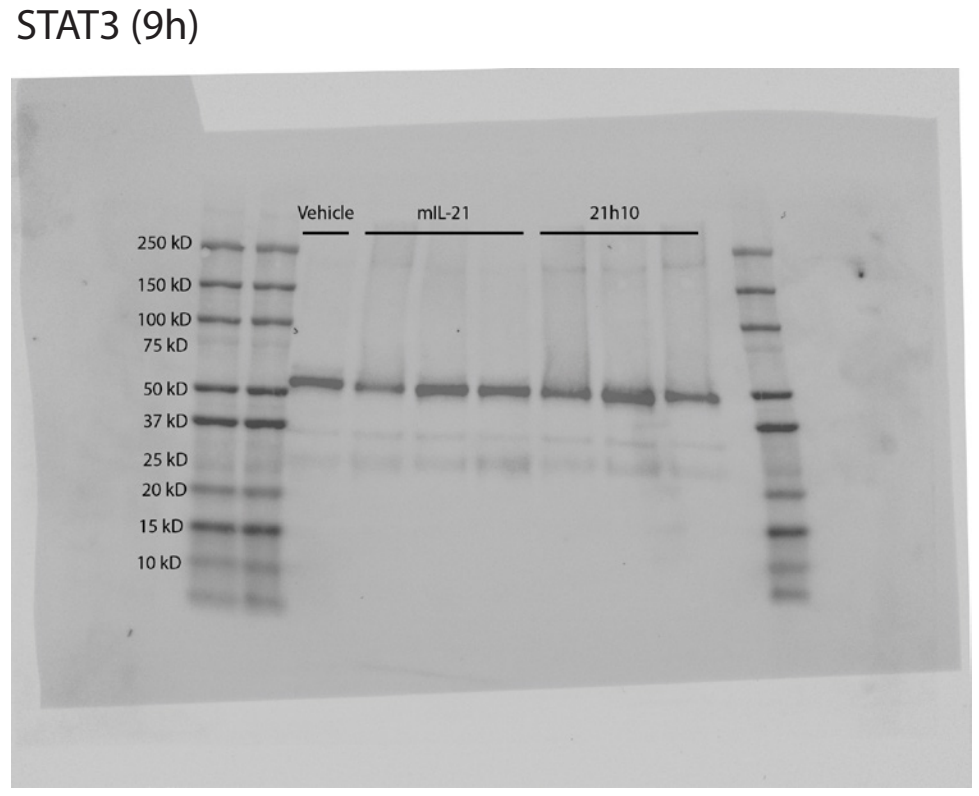

STAT3 (24h)

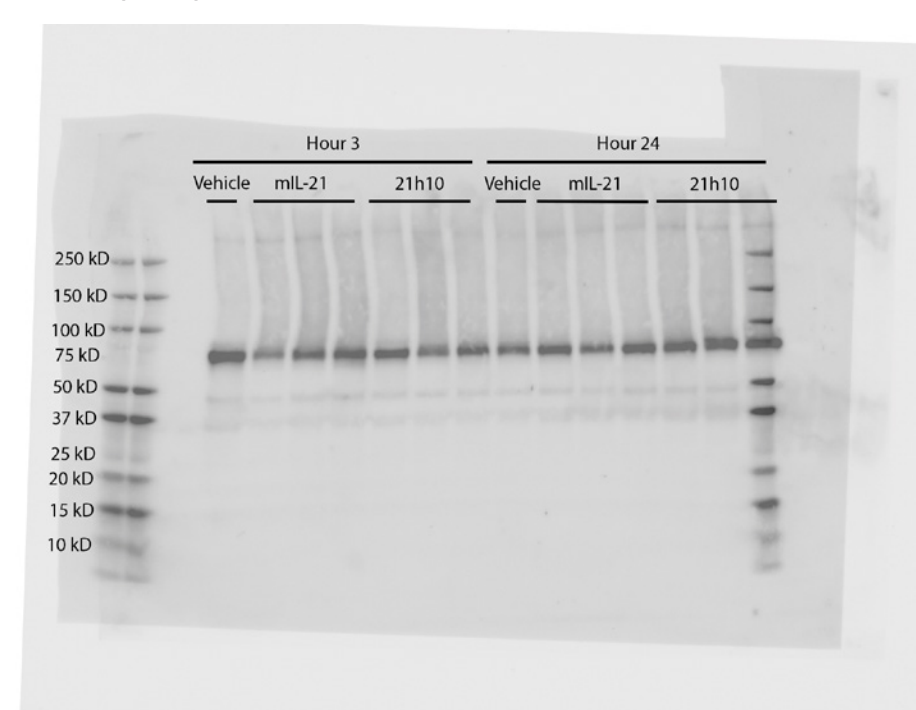

Supplement: Supplementary file 2 [file NIHMS2111844-supplement-SM2.pdf]
