# Supplementary material for: Design of a potent interleukin-21 mimic for cancer immunotherapy
Source: Sci Immunol. Author manuscript; Available in PMC 2026 Jan 6. (PMC12768534; doi:10.1126/sciimmunol.adx1582)
Supplement: Supplementary file 3 [file NIHMS2111844-supplement-SM3.pdf]

CAR construct information

1. CD19 CAR full plasmid sequence

tcaaggttaggaacagagagacagcagaatatgggccaaacaggatatctgtggtaagcagttcctgccccggctcagggccaagaacagttggaacagcagaatatgggccaaacaggatatc
tgtggtaagcagttcctgccccggctcagggccaagaacagatggtccccagatgcggtcccgccctcagcagtttctagagaaccatcagatgtttccagggtgccccaaggacctgaaatgacc
ctgtgccttatttgaactaaccaatcagttcgcttctcgcttctgttcgcgcgcttctgctccccgagctcaataaaagagcccacaacccctcactcggcgcgccagtcctccgatagactgcgtcgcc
cgggtacccgtattcccaataaagcctcttgctgtttgcatccgaatcgtggactcgctgatccttgggagggtctcctcagattgattgactgcccacctcgggggtctttcatttggaggttccaccga
gatttggagacccctgcccagggaccaccgacccccccgccgggaggtaagctggccagcggtcgtttcgtgtctgtctctgtctttgtgcgtgtttgtgccggcatctaatgtttgcgcctgcgtctgt
actagttggctaactagatctgtatctggcggtcccgcggaagaactgacgagttcgtattcccggccgcagcccctgggagacgtcccagcggcctcgggggcccgttttgtggcccattctgtat
cagttaacctacccgagtcggactttttggagctccgccactgtccgaggggtacgtggctttgttgggggacgagagacagagacacttcccgcccccgtctgaatttttgctttcggttttacgccga
aaccgcgccgcgcgtcttgtctgctgcagcatcgttctgtgttgtctctgtctgactgtgtttctgtatttgtctgaaaattagctcgacaaagttaagtaatagtccctctctccaagctcacttacaggcta
gcgccgccaccATGGCTTTGCCAGTGACAGCTCTTCTCCTTCCACTGGCCCTCCTCCTTCACGCCGCTAGGCCAGAGCAGAAA
CTTATTTCAGAGGAAGACCTGGACATTCAAATGACACAAACTACTTCTTCTCTCTCCGCCTCACTTGGTGACCGCGTCAC
TATTAGTTGCCGCGCTAGTCAAGATATTAGTAAGTACCTGAATTGGTATCAACAAAAACCTGACGGGACTGTAAAGCTG
CTTATATATCATACTTCTAGGCTGCATTCTGGAGTACCTTCACGATTTAGCGGTAGCGGATCCGGCACCGACTACTCCCT
CACAATTAGCAATCTGGAGCAAGAGGACATAGCCACCTACTTCTGCCAGCAAGGGAATACCTTGCCATACACTTTCGGT
GGTGGAACTAAGCTCGAAATTACTGGGGGTGGAGGCAGTGGCGGAGGGGGGTCAGGTGGGGGAGGTTCAGAAGTCAA
ACTCCAGGAATCTGGACCTGGACTCGTTGCCCCTTCCCAATCCCTTAGTGTTACATGCACTGTATCAGGTGTATCCCTCC
CTGATTACGGTGTCTCCTGGATTCGGCAGCCTCCTCGGAAGGGTCTCGAGTGGTTGGGAGTGATTTGGGGGTCTGAAAC
TACTTATTATAACAGTGCCCTTAAGAGTAGATTGACTATAATTAAGGATAACAGTAAGTCACAAGTATTCCTCAAAATG
AATTCCTTGCAAACAGACGATACAGCAATATATTACTGCGCAAAACACTACTACTATGGCGGTAGTTACGCTATGGACT
ATTGGGGTCAAGGAACCTCTGTCACAGTTTCTAGCgagtccaaatatggtcccccatgcccaccctgcccatttgggcactggtcgtggttgctggagtcctgtttt
gttatggcttgctagtgacagtggctctttgtgttatctggacaaatagtagaaggaacagaggcggtcaaagtgactacatgaacatgactccccggaggcctgggctcactcgaaagccttaccag
ccctacgcccctgccagagactttgcagcgtaccgccccaaatggatcaggaaaaaattcccccacatattcaagcaaccatttaagaagaccactggagcagctcaagaggaagatgcttgtagc
tgccgatgtccacaggaagaagaaggaggaggaggaggctatgagctgagagcaaaattcagcaggagtgcagagactgctgccaacctgcaggaccccaaccagctctacaatgagctcaat
ctagggcgaagagaggaatatgacgtcttggagaagaagcgggctcgggatccagagatgggaggcaaacagcagaggaggaggaacccccaggaaggcgtatacaatgcactgcagaaa
gacaagatggcagaagcctacagtgagatcggcacaaaaggcgagaggcggagaggcaaggggcacgatggcctttaccagggtctcagcactgccaccaaggacacctatgatgccctgca
tatgcagaccctggcccctcgcctcgagggcggcggagagggcagaggaagtcttctaacatgcggtgacgtggaggagaatcccggccctaggatgaacccagccatcagcgtcgctctcct
gctctcagtcttgcaggtgtcccgagggcagaaggtgaccagcctgacagcctgcctggtgaaccaaaaccttcgcctggactgccgccatgagaataacaccaaggataactccatccagcatg
agttcagcctgacccgagagaagaggaagcacgtgctctcaggcacccttgggatacccgagcacacgtaccgctcccgcgtcaccctctccaaccagccctatatcaaggtccttaccctagcca
acttcaccaccaaggatgagggcgactacttttgtgagcttcgcgtctcgggcgcgaatcccatgagctccaataaaagtatcagtgtgtatagagacaagctggtcaagtgtggcggcataagcct
gctggttcagaacacatcctggatgctgctgctgctgctttccctctccctcctccaagccctggacttcatttctctgtgagcggccgcctctagacccggggagcatcttaccgccatttattcccatat
ttgttctgtttttcttgatttgggtatacatttaaatgttaataaaacaaaatggtggggcaatcatttacattttatgggatatgtaattactagttcaggtgtattgccacaagacaaacatgttaagaaactttc
ccgttatttacgctctgttcctgttaatcaacctctggattacaaaatttgtgaaagattgactgatattcttaactatgttgctccttttacgctgtgtggatatgctgctttaatgcctctgtatcatgctattgctt
cccgtacggctttcgttttctcctccttgtataaatcctggttgctgtctctttatgaggagttgtggcccgttgtccgtcaacgtggcgtggtgtgctctgtgtttgctgacgcaacccccactggctgggg
cattgccaccacctgtcaactcctttctgggactttcgctttccccctcccgatcgccacggcagaactcatcgccgcctgccttgcccgctgctggacaggggctaggttgctgggcactgataattc
cgtggtgttgtcggggaagctgacgtcctttccatggctgctcgcctgtgttgccaactggatcctgcgcgggacgtccttctgctacgtcccttcggctctcaatccagcggaccttccttcccgagg
ccttctgccggttctgcggcctctcccgcgtcttcgctttcggcctccgacgagtcggatctccctttgggccgcctccccgcctgtttcgcctcggcgtccggtccgtgttgcttggtcgtcacctgtgc
agaattgcgaaccatggattccaccgtgaactttgtctcctggcatgcaaatcgtcaacttggcatgccaagaattaattcggatccaagcttaggcctgctcgctttcttgctgtcccatttctattaaagg
ttcctttgttccctaagtccaactactaaactgggggatattatgaagggccttgagcatctggattctgcctagcgctaagcttaacacgagccatagatagaataaaagattttatttagtctccagaaa
aaggggggaatgaaagaccccacctgtaggtttggcaagcaagtttaagtaacgccattttgcaaggcatggaaaatacataactgagaatagagaagttcagatcaaggttaggaacagagagac
agcagaatatgggccaaacaggatatctgtggtaagcagttcctgccccggctcagggccaagaacagttggaacagcagaatatgggccaaacaggatatctgtggtaagcagttcctgccccg
gctcagggccaagaacagatggtccccagatgcggtcccgccctcagcagtttctagagaaccatcagatgtttccagggtgccccaaggacctgaaatgaccctgtgccttatttgaactaaccaa
tcagttcgcttctcgcttctgttcgcgcgcttctgctccccgagctcaataaaagagcccacaacccctcactcggcgcgccagtcctccgatagactgcgtcgcccgggtacccgtgttctcaataaa
ccctcttgcagttgcatccgactcgtggtctcgctgttccttgggagggtctcctctgagtgattgactgcccacctcgggggtctttcattctcgagcagcttggcgtaatcatggtcatagctgtttcctg
tgtgaaattgttatccgctcacaattccacacaacatacgagccggaagcataaagtgtaaagcctggggtgcctaatgagtgagctaactcacattaattgcgttgcgctcactgcccgctttccagtc
gggaaacctgtcgtgccagctgcattaatgaatcggccaacgcgcggggagaggcggtttgcgtattgggcgctcttccgcttcctcgctcactgactcgctgcgctcggtcgttcggctgcggcga
gcggtatcagctcactcaaaggcggtaatacggttatccacagaatcaggggataacgcaggaaagaacatgtgagcaaaaggccagcaaaaggccaggaaccgtaaaaaggccgcgttgctg
gcgtttttccataggctccgcccccctgacgagcatcacaaaaatcgacgctcaagtcagaggtggcgaaacccgacaggactataaagataccaggcgtttccccctggaagctccctcgtgcgc
tctcctgttccgaccctgccgcttaccggatacctgtccgcctttctcccttcgggaagcgtggcgctttctcatagctcacgctgtaggtatctcagttcggtgtaggtcgttcgctccaagctgggctgt
gtgcacgaaccccccgttcagcccgaccgctgcgccttatccggtaactatcgtcttgagtccaacccggtaagacacgacttatcgccactggcagcagccactggtaacaggattagcagagcg

aggtatgtaggcggtgctacagagttcttgaagtggtggcctaactacggctacactagaagaacagtatttggtatctgcgctctgctgaagccagttaccttcggaaaaagagttggtagctcttgat
ccggcaaacaaaccaccgctggtagcggtggtttttttgtttgcaagcagcagattacgcgcagaaaaaaaggatctcaagaagatcctttgatcttttctacggggtctgacgctcagtggaacgaaa
actcacgttaagggattttggtcatgagattatcaaaaaggatcttcacctagatccttttaaattaaaaatgaagttttaaatcaatctaaagtatatatgagtaaacttggtctgacagttaccaatgcttaat
cagtgaggcacctatctcagcgatctgtctatttcgttcatccatagttgcctgactccccgtcgtgtagataactacgatacgggagggcttaccatctggccccagtgctgcaatgataccgcgagac
ccacgctcaccggctccagatttatcagcaataaaccagccagccggaagggccgagcgcagaagtggtcctgcaactttatccgcctccatccagtctattaattgttgccgggaagctagagtaa
gtagttcgccagttaatagtttgcgcaacgttgttgccattgctacaggcatcgtggtgtcacgctcgtcgtttggtatggcttcattcagctccggttcccaacgatcaaggcgagttacatgatcccccа
tgttgtgcaaaaaagcggttagctccttcggtcctccgatcgttgtcagaagtaagttggccgcagtgttatcactcatggttatggcagcactgcataattctcttactgtcatgccatccgtaagatgctt
ttctgtgactggtgagtactcaaccaagtcattctgagaatagtgtatgcggcgaccgagttgctcttgcccggcgtcaatacgggataataccgcgccacatagcagaactttaaaagtgctcatcatt
ggaaaacgttcttcggggcgaaaactctcaaggatcttaccgctgttgagatccagttcgatgtaacccactcgtgcacccaactgatcttcagcatcttttactttcaccagcgtttctgggtgagcaaa
aacaggaaggcaaaatgccgcaaaaaagggaataagggcgacacggaaatgttgaatactcatactcttcctttttcaatattattgaagcatttatcagggttattgtctcatgagcggatacatatttg
aatgtatttagaaaaataaacaaataggggttccgcgcacatttccccgaaaagtgccacctgacgtctaagaaaccattattatcatgacattaacctataaaaataggcgtatcacgaggccctttcgt
ctcgcgcgtttcggtgatgacggtgaaaacctctgacacatgcagctcccggagacggtcacagcttgtctgtaagcggatgccgggagcagacaagcccgtcagggcgcgtcagcgggtgttg
gcgggtgtcggggctggcttaactatgcggcatcagagcagattgtactgagagtgcaccatatgcggtgtgaaataccgcacagatgcgtaaggagaaaataccgcatcaggcgccattcgccat
tcaggctgcgcaactgttgggaagggcgatcggtgcgggcctcttcgctattacgccagctggcgaaagggggatgtgctgcaaggcgattaagttgggtaacgccagggttttcccagtcacgac
gttgtaaaacgacggccagtgaattagtactctagcttaagtaacgccattttgcaaggcatggaaaatacataactgagaatagagaagttcaga

2. CD19 CAR insert sequence

ATGGCTTTGCCAGTGACAGCTCTTCTCCTTCCACTGGCCCTCCTCCTTCACGCCGCTAGGCCAGAGCAGAAACTTATTTC
AGAGGAAGACCTGGACATTCAAATGACACAAACTACTTCTTCTCTCTCCGCCTCACTTGGTGACCGCGTCACTATTAGT
TGCCGCGCTAGTCAAGATATTAGTAAGTACCTGAATTGGTATCAACAAAAACCTGACGGGACTGTAAAGCTGCTTATAT
ATCATACTTCTAGGCTGCATTCTGGAGTACCTTCACGATTTAGCGGTAGCGGATCCGGCACCGACTACTCCCTCACAATT
AGCAATCTGGAGCAAGAGGACATAGCCACCTACTTCTGCCAGCAAGGGAATACCTTGCCATACACTTTCGGTGGTGGA
ACTAAGCTCGAAATTACTGGGGGTGGAGGCAGTGGCGGAGGGGGGTCAGGTGGGGGAGGTTCAGAAGTCAAACTCCA
GGAATCTGGACCTGGACTCGTTGCCCCTTCCCAATCCCTTAGTGTTACATGCACTGTATCAGGTGTATCCCTCCCTGATT
ACGGTGTCTCCTGGATTCGGCAGCCTCCTCGGAAGGGTCTCGAGTGGTTGGGAGTGATTTGGGGGTCTGAAACTACTTA
TTATAACAGTGCCCTTAAGAGTAGATTGACTATAATTAAGGATAACAGTAAGTCACAAGTATTCCTCAAAATGAATTCC
TTGCAAACAGACGATACAGCAATATATTACTGCGCAAAACACTACTACTATGGCGGTAGTTACGCTATGGACTATTGGG
GTCAAGGAACCTCTGTCACAGTTTCTAGCgagtccaaatatggtcccccatgcccaccctgcccattttgggcactggtcgtggttgctggagtcctgttttgttatggcttg
ctagtgacagtggctctttgtgttatctggacaaatagtagaaggaacagaggcggtcaaagtgactacatgaacatgactccccggaggcctgggctcactcgaaagccttaccagccctacgccc
ctgccagagactttgcagcgtaccgccccaaatggatcaggaaaaaattcccccacatattcaagcaaccatttaagaagaccactggagcagctcaagaggaagatgcttgtagctgccgatgtcc
acaggaagaagaaggaggaggaggaggctatgagctgagagcaaaattcagcaggagtgcagagactgctgccaacctgcaggaccccaaccagctctacaatgagctcaatctagggcgaa
gagaggaatatgacgtcttggagaagaagcgggctcgggatccagagatgggaggcaaacagcagaggaggaggaacccccaggaaggcgtatacaatgcactgcagaaagacaagatgg
cagaagcctacagtgagatcggcacaaaaggcgagaggcggagaggcaaggggcacgatggcctttaccagggtctcagcactgccaccaaggacacctatgatgccctgcatatgcagacc
ctggcccctcgcctcgagggcggcggagagggcagaggaagtcttctaacatgcggtgacgtggaggagaatcccggccctaggatgaacccagccatcagcgtcgctctcctgctctcagtctt
gcaggtgtcccgagggcagaaggtgaccagcctgacagcctgcctggtgaaccaaaaccttcgcctggactgccgccatgagaataacaccaaggataactccatccagcatgagttcagcctg
acccgagagaagaggaagcacgtgctctcaggcacccttgggatacccgagcacacgtaccgctcccgcgtcaccctctccaaccagccctatatcaaggtccttaccctagccaacttcaccacc
aaggatgagggcgactacttttgtgagcttcgcgtctcgggcgcgaatcccatgagctccaataaaagtatcagtgtgtatagagacaagctggtcaagtgtggcggcataagcctgctggttcaga
acacatcctggatgctgctgctgctgctttccctctccctcctccaagccctggacttcatttctctgtga
