# Supplementary material for: Design of a potent interleukin-21 mimic for cancer immunotherapy
Source: Sci Immunol. Author manuscript; Available in PMC 2026 Jan 6. (PMC12768534; doi:10.1126/sciimmunol.adx1582)
Supplement: Supplementary file 4 [file NIHMS2111844-supplement-Supplementary_Materials.pdf]

SUPPLEMENTARY MATERIALS AND METHODS

Yeast display and fluorescent-activated cell sorting

The amino acid sequences of the designed proteins were reverse-translated to DNA sequences based on the codon frequency table of *Saccharomyces cerevisiae,* along with pETcon3 vector-homologous sequences appended to both 5' and 3' termini. Using competent yeast cells, *EBY100*, the DNA gene blocks of the designs were cloned into linearized pETcon3 vectors by yeast homologous recombination. The vector was linearized by 100-fold overdigestion by NdeI and XhoI (New England Biolabs) and then purified by gel extraction (Qiagen). Yeast transformations are validated by colony sequencing; validated colonies are grown in C-Trp-Ura medium and induced in SGCAA medium as previously described(*80*). SGCAA induced the designed proteins to be expressed and presented on the yeast surface through Aga2p membrane proteins. The induced cells with the designed proteins on their surfaces were labeled with two fluorophores - FITC and PE. Anti-c-myc mAb conjugated to FITC was used to label the myc tag to represent the degree of protein expression. For IL-21R labeling, either human or murine IL-21R with human Fc-tag (R&D Systems Cat# 991-R2, R&D Systems Cat# 596-MR) were used, and for receptor complex labeling, either human IL-21R with His-tag (R&D Systems Cat# 9249-R2) and human γ_c with Fc-tag (Acro Biosystems Cat# ILG-H5256), or mIL-21R with His-tag (Sino Biological Cat# 51184-M08H) and murine γ_c with Fc-tag (R&D Systems Cat# 784-MR) were used. The target receptor fused with the Fc domain is labeled by the biotinylated ZZ domain of protein A and streptavidin-PE (SA-PE), which can bind to the biotin of protein A. PE represents the level of receptor binding. For binding hits, their signal is proportional to the protein expression level. Yeast was analyzed by flow cytometers (BD Accuri C6, Thermo Fisher Attune NxT) or sorted by a fluorescence-activated cell sorting (FACS) cell sorter (Sony SH800).

Mutagenesis and affinity maturation

Using a random mutagenesis library kit (Agilent Cat# 200550), the 21d26 DNA was amplified, and the library was partially sequenced using BL21(DE3) (New England Biolabs). The sequencing showed that approximately 2-4 mutations were applied to the design while the DNA was amplified with the error-prone PCR. Next, the library was transformed to yeast using electroporation with an approximated diversity of 3.4E7. The yeast library was sorted for direct evolution using FACS with different combinations and concentrations of labeled receptor subunits with the abovementioned labeling. With three consecutive sorts with either human receptor complex labeling (1 µM of unlabeled hIL-21R and 1 µM of labeled hγ_c) saturated sorted library with mutants (including 21JC15) with hγ_c-binding capability.

To analyze and enhance the interface affinities of the interfaces of the 21JC15, we used SSM to analyze the two interfaces of the protein in detail. The protein sequence of interest was split into halves with corresponding single mutations and synthesized as two fragments for assembly (Integrated DNA Technologies). Assembly was done with PCR with pETcon3-specific 5' and 3' primers. Sortings with four different receptor conditions – (1) labeled hIL-21R, (2) unlabeled hIL-21R with labeled hγ_c, (3) labeled mIL-21R, and (4) unlabeled mIL-21R with labeled mγ_c – saturated mutants with mutants with favorable mutations, and depleted mutants with unfavorable mutations. DNA of the sorted SSM library was prepared prior to MiSeq (Illumina Cat# MS-102-3001) using Zymoprep (Zymo Research Cat# D2001), qPCR, and gel extraction (Qiagen). The sorted SSM library revealed several mutations that can improve binding affinities to each receptor - hIL-21R, hγ_c, mIL-21R, and mγ_c.

By merging the positive mutations, we built a combinatorial library that incorporated the mutations by using a PCR-based assembly of pools of 8 sets of primers (Integrated DNA Technologies) incorporating mutations with degenerate codons, which was then sorted against human or murine receptors. Sorting it yielded multiple candidates with significantly improved affinities to the receptors. The K_D of the candidates toward each of the receptors was measured by biolayer interferometry (BLI) (ForteBio).

Protein expression and endotoxin removal

The DNA fragments (Integrated DNA Technologies) that encode the designed proteins were cloned into pET-29b(+) plasmid with an N-terminal polyhistidine tag. The cloned plasmids were transformed into competent BL21 cells (New England Biolabs), whose cultures were grown in Terrific Broth II and induced using 1 mM isopropyl b-D-thiogalactopyranoside (IPTG). The cultures were grown in baffled flasks at 37°C within 225 rpm shakers until they reached approximately

OD_{A600}=0.8 for induction. The cultures were induced for 14 hours at 18C. The harvested cultures were lysed by sonication (Qsonica Q500). The lysed cultures were ultracentrifuged at 18,000g for 30 minutes and purified using immobilized metal affinity chromatography (IMAC). The elution proteins were separated by HPLC size-exclusion chromatography using Superdex 75 10/300 GL column (GE Healthcare) on Akta (GE Healthcare AKTA Pure). Endotoxins in protein samples from *Escherichia coli* cultures were removed by loading the SEC-purified protein onto the Ni-NTA column and washing with endo-removal IMAC wash buffer (TBS, 1% CHAPS), which were then eluted, and the imidazole was removed by dialysis. The endotoxin levels were measured using Endosafe LAL cartridge with 0.1 EU/mL sensitivity (Charles River PTS201F) on Endosafe machine (Charles River NexGen-MCS).

Protein expression of 21h10 receptor complex for crystallography

21h10 was expressed in *Escherichia coli* with a polyhistidine tag on its N-terminus and TEV protease cleavage site in between. After expression, the polyhistidine tag was removed using polyhistidine-tagged TEV protease and re-purified by running the lysate through Ni-NTA resin to remove TEV protease and size exclusion chromatography to purify the 21h10. The protein was then concentrated using a 5 kDa MWCO Amicon concentrator. The hIL-21R extracellular domain with glycan-reduced mutations (N78Q/N85Q/N106D/N116Q) was cloned into the pAcGP67a baculovirus vector with a C-terminal 6xHis-tag, and baculovirus was prepared by co-transfection of BestBac™ DNA (Expression Systems) and pAcGP67a DNA into *Spodoptera frugiperda* (Sf9) cells. The virus harvested from Sf9 cells was used to infect *Trichoplusia ni* (Hi5) cells. The supernatant was harvested from Hi5 cells 72 hours after infection and incubated together with 21h10 on Ni-NTA resin (Qiagen) for 3 hours prior to elution with 0.3 M imidazole followed by purification using size-exclusion chromatography (SEC) on a Superdex 200 column (GE Healthcare). Finally, the hγ_c extracellular domain with an N-terminal truncation of 32 residues was cloned into pAcGP67a for baculovirus generation, and Hi5 cells were infected in culture media containing 5 µM kifunensine. The supernatant was harvested from Hi5 cells 72 hours after infection, deglycosylated by overnight incubation with Endo Hf (New England Biolabs), and purified by SEC on a Superdex 200 column.

Biolayer interferometry

Binding data were collected using OctetRED96 and OctetR8 (Sartorius). Octet binding buffer (HEPES+1% w/v Bovine Serum Albumin) was used for all reactions. To measure the binding affinity to hIL-21R or mIL-21R, biotinylated hIL-21R (R&D Systems Cat# AVI9249) or biotinylated mIL-21R (Acro Biosystems Cat# ILR-M82E3), respectively, were immobilized on streptavidin-coated biosensors (SAForteBio) at 5 µg/mL in the octet binding buffer, with 2-fold serial dilutions of the ligand starting at 100 nM, with 300 seconds for association and another 300 seconds for dissociation. Similarly, to measure binding affinity of 21AT36 to hIL-21R or mIL-21R, biotinylated hIL-21R (R&D Systems Cat# AVI9249) or biotinylated mIL-21R (Acro Biosystems Cat# ILR-M82E3), respectively, were immobilized on streptavidin-coated biosensors (SAForteBio) at 5 µg/mL in the octet binding buffer, with 3-fold serial dilutions of the ligand starting at 100 nM, with 300 seconds for association and another 300 seconds for dissociation.

To measure the binding affinity to hIL-21R/hγ_c or mIL-21R/mγ_c, biotinylated hγ_c (Acro Biosystems Cat# ILG-H85E8) or biotinylated mγ_c (Acro Biosystems Cat# ILA-M82E3), respectively, were immobilized on streptavidin-coated biosensors (SAForteBio) at 5 µg/mL in the octet binding buffer and tested with 3-fold serial dilutions of the ligand starting at 500 nM, for 300-second association and 300-second dissociation in the presence of 1.5-fold molar excess of corresponding hIL-21R (R&D Systems Cat# 9249-R2-100) or mIL-21R (Sino Biological Cat# 51184-M08H). For ligands, hIL-21 (R&D Systems Cat# 8879-IL), mIL-21 (R&D Systems Cat# 594-ML), and 21h10 were used. Data was processed using ForteBio Data Analysis Software version 9.0.0.10. and the parameters were reported with standard error. All data are summarized in table S2.

Circular dichroism and thermal stability assay

Far-ultraviolet circular dichroism measurements were performed with a spectropolarimeter (JASCO J-1500). The proteins were dissolved in PBS (pH 7.4) within 1 mm path length cuvettes at 0.5 mg/mL. The wavelength scan was performed within the 195 nm to 260 nm wavelength range. The thermal melt experiment was performed by heating samples (step 2°C/minute) from 25°C to 95°C and cooled back down to 25°C while observing absorption at 222 nm.

STAT phosphorylation assay in B cells

All experiments involving mice at NHLBI were performed using protocols approved by the NHLBI Animal Care and Use Committee and followed National Institutes of Health (NIH) guidelines for the use of animals in intramural research. Murine B cells were isolated from the spleens of 6- to 8-week-old C57BL/6 mice through negative selection and magnetic separation (Stem Cell Technologies Cat# 19854). Purified murine B cells were pre-activated with anti-mouse CD40 (clone FGK45, BioLegend Cat# 157503) for 48 hours at 37°C in complete RPMI media. Human B cells were isolated from the buffy coats from healthy human volunteers obtained from NIH Blood Bank after written informed consent in accordance with the approval of the institutional human ethics committee. Peripheral blood mononuclear cells (PBMCs) were isolated using a lymphocyte separation medium based on density gradient centrifugation. Human B cells were then purified by negative selection and magnetic separation (Stem Cell Technologies Cat# 17954). Purified human B cells were pre-activated with anti-human CD40 (clone HB14, BioLegend Cat# 313020) for 48 hours at 37°C in complete RPMI media.

In vitro CD8$^+$ T cell culture for flow cytometry

Purified murine and human CD8$^+$ T cells were cultured in the presence of anti-mouse CD3 and CD28 or anti-human CD3 and CD28, respectively (as described above), together with mIL-21 or hIL-21 and 21h10 at 1, 10, 100 and 1000 ng/mL concentrations in complete RPMI medium for 72 hours at 37°C. Cells were harvested and stained with either APC anti-mouse IL-21R (1:100, Biolegend, Cat#131910) or APC anti-human IL-21R (1:100, Biolegend, Cat#347808) and BV421 anti-mouse/human granzyme B (1:100, Biolegend, Cat#396414) for flow cytometry. Cells were analyzed using a BD LSR Fortessa X-20 Cell Analyzer flow cytometer (BD Biosciences).

Murine CD8$^+$ T cell proliferation assay

For *in vitro* proliferation experiment, CD8$^+$ T cells isolated from TRP1^{high} mice using EasySep Mouse CD8$^+$ T Cell Isolation Kit (StemCell Cat# 19853) were stained with Cell Trace Violet (Invitrogen Cat# C34557) at a 1:1000 dilution in PBS and plated at 50,000 cells/well in a 96-well plate. Cells were plated with 3 µL/million cells of Dynabeads Mouse T-Activator CD3/CD28 (Gibco Cat# 11453D) along with various concentrations (0.001-10 nM) of mIL-21 (R&D Systems Cat# 594-ML-100/CF) or 21h10 in RPMI complete. Cytokines were incubated prior to addition to the cells for 24 hours at 37°C in healthy human serum. After 72 hours of treatment, cells were collected and analyzed by flow cytometry for CTV-labeled populations along with the following: APC anti-CD8a (clone 53-6.7, BioLegend Cat# 100712), Brilliant Violet 711 anti-CD44 (clone IM7, BioLegend Cat# 103057), PE anti-CD69 (clone H1.2F3, BioLegend Cat# 104508), and Zombie NIR (BioLegend Cat# 423105). Zombie NIR was used at a 1:500 dilution and antibodies were used at a 1:100 dilution.

RNA-seq preparation

Murine CD8$^+$ T cells were isolated from the spleens of 6- to 8-week-old C57BL/6 mice through negative selection and magnetic separation (Stem Cell Technologies Cat# 19853). Purified murine CD8$^+$ T cells were pre-activated with plate-bound anti-mouse CD3 (2 µg/mL; clone 145-2C11, BioXCell Cat# BE0001-1) and soluble anti-mouse CD28 (1 µg/mL; clone 37.51, BioXCell Cat# BE0015-1) and cultured in complete RPMI media for 48 hours at 37°C. Cells were rested overnight and restimulated with mIL-21 or 21h10 at 100 pM, 1 nM, 10 nM, and 100 nM concentrations for 24 hours in complete RPMI media at 37°C. Cells were then lysed in TRIzol reagent (Invitrogen Cat# 15596026), RNA was isolated using Direct-zol RNA MiniPrep Kit (Zymo Research Cat# R2052), and the libraries were prepared for RNA-Sequencing using KAPA mRNA HyperPrep Kit (Roche Cat# KK8440) that was performed in the NHLBI DNA sequencing core.

Murine T cell differentiation assay

All mice underwent experiments following guidance from the University of Washington Institutional Animal Care and Use Committee, protocol number 4397-01. Mice used for experiments harbored a non-perturbing Yellow Fluorescent Protein (YFP) reporter at the endogenous *Tcf7* locus as previously described(*81*) and were donated by Avinash Bhandoola. Spleens were harvested from mice, and a single-cell suspension was generated by massaging spleens between rough glass slides and filtering over 50 µm mesh. Red blood cells (RBC) were lysed in RBC lysis buffer (150 mM NH_4CL, 10 mM $NaHCO_3$, 1 mM EDTA) for 3-5 minutes at room temperature. Splenocytes were incubated in Fc blocking buffer (10% 2.4G2 cell supernatant in FACS buffer, 0.5% BSA in HBSS) prior to CD8$^+$ T cell isolation. CD8$^+$ T cells were isolated using the CD8a$^+$ T Cell isolation kit (Miltenyi Cat# 130-095-236) following the manufacturer's instructions. CD8$^+$ T cells were counted, stained with 5 µM CellTrace Violet (ThermoFisher) for 20 minutes at room temperature, and quenched with RPMI medium with 10%

Fetal Bovine Serum (VWR) prior to activation.

One day prior to activation, tissue culture plates were coated with 1 μg/mL anti-CD3ε (BioLegend, Ultra-LEAF, Clone 145-2C11) and 0.5 μg/mL anti-CD28 (CyTek *in vivo* ready, Clone 37.51) in PBS and incubated overnight at 4°C. On the day of activation, plates were allowed to reach room temperature, washed 2x with room temperature PBS, and CTV-labeled T cells were resuspended in T cell medium (TCM, 85% RPMI 1640 with L-glutamine, 10% heat-inactivated Fetal Bovine Serum, 1x Pen-Strep-Glutamine, 20 mM HEPES, 1 mM Sodium Pyruvate, 0.1 mM Non Essential Amino Acids, and 50 μM β-mercaptoethanol (BME)) supplemented with 100 U/mL recombinant human IL-2 (Peprotech Cat# 200-02). T cells were seeded at $3x10^5$ cells per well of a pre-coated 24-well culture plate in 1.2 mL of TCM. Cells were incubated at 37°C 5% CO_2.

After 48 hours, T cells were pooled, counted, centrifuged, and resuspended at $2.5x10^5$ cells/mL in a 24- or 96-well cell culture plate in TCM at 200 μL/well in uncoated 96-well plates and 1.2 mL/well in 24 well plates. TCM was supplemented as indicated in each figure either with PBS, mIL-21 (R&D Systems Cat# 594-ML), or 21h10 diluted in PBS carrier to a final concentration of 0.01 nM, 1 nM, and 100 nM. Cells were incubated at 37°C 5% CO_2 for an additional 48 hours and collected directly for extracellular flow cytometry analysis or counted and re-stimulated for intracellular cytokine staining (ICS). For the percentage of IFN-γ-secreting cells, samples were quantified after 5 hour stimulation with PMA/ionomycin in the presence of a protein transport inhibitor.

For ICS, cells were counted and resuspended in round bottom 96 well plates at $2x10^5$ cells per 100 μL of 1x Cell Stimulation Cocktail (eBioscience 500x stock) and incubated at 37°C 5% CO_2 for 1 hour. Cells were then supplemented with Protein Transport Inhibitor (eBioscience 500x stock) to a final concentration of 1x and incubated at 37°C 5% CO_2 for an additional 4 hours. Cells were then stained with 1:100 dilution Zombie Near IR (BioLegend Cat# 423105) for 15 minutes at room temperature, centrifuged and resuspended in the Fc blocking buffer, and incubated for 15 minutes on ice. Cells were then fixed and permeabilized for 15 minutes on ice using 100 μL/well of Cytofix/Cytoperm (BD), washed 2x with 1x Perm/Wash Buffer (BD), and incubated with anti-IFN-γ (BioLegend, clone XMG1.2, PE) at 1:100 in 1x Perm/Wash Buffer for 30 minutes on ice. Cells were washed by an additional 2x with 1x Perm/Wash buffer and resuspended in FACS buffer for analysis on the Attune NxT (ThermoFisher).

For extracellular flow staining, cells were transferred to 96-well round bottom plates, centrifuged, resuspended in 12.5 μL of Fc blocking buffer, and incubated on ice for 15 minutes. Cells were stained for 20 minutes on ice in 25 μL total volume of antibodies in the Fc blocking buffer. The following antibodies were used at the following dilutions: anti-CD62L, 1:1200 (eBioscience, clone MEL-14, APC-eFluor780), anti-CD44, 1:1200 (eBioscience, clone IM7, PerCP-Cyanine5.5), anti-CD25, 1:1200 (BioLegend, clone PC61, BV510), anti-CD366/Tim-3, 1:100 (BioLegend, clone RMT3-23, APC), anti-CD127, 1:100 (eBioscience, clone A7R34, PE). After staining, cells were centrifuged, resuspended in FACS buffer, and analyzed on an Attune NxT system (ThermoFisher). Data were analyzed using FlowJo software (BD). Statistical analysis was performed using R. For all experiments, n=2 or 3 biological replicates. Replicates are indicated as individual dots in plots.

In vivo $CD8^+$ T cell effector differentiation study in the context of viral infection

5- to 7-week-old female C57BL/6 mice were purchased from Jackson Laboratory (Bar Harbor, ME, USA) for this study. $H2\text{-}D^b$GP33-specific $Thy1.1^+$ TCR-transgenic P14 mice were maintained in our colony. Mice were used in accordance with Institutional Animal Care and Use Committee (IACUC) guidelines. Naïve ~2000 GP33-specific P14 $CD8^+$ T cells were adoptively transferred into B6 mice, followed by intravenous infection with Lymphocytic choriomeningitis virus (LCMV) ($2x10^6$ PFU $LCMV_{Cl13}$). Three PBS, mIL-21, and 21h10, were administered daily intraperitoneally at 2.5 μg per mouse from day 0 to day 6 after infection. Mice were euthanized on day 7 post-infection. Blood and spleen were collected from each mouse, and antigen-specific $CD8^+$ T cells were analyzed for effector molecule granzyme B expression using flow cytometry by gating GP33-specific P14 $CD8^+$ T cells using the Thy1.1 congenic marker.

CAR-T cell generation and maintenance

The retroviral vector expressing the CD19 chimeric antigen receptor (CAR) was constructed using sequences from the clone FMC63 human CD19 single chain variable fragment, and the murine CD28 and CD3ζ sequences. A Myc tag was added to the N-terminus of the CAR construct. After the CAR sequence, a T2A protein cleavage site was inserted, followed by Thy1.1 marker sequence. This CAR construct (Data file S4) was then cloned into murine retroviral vector MP71. The Plat E retroviral packaging cell line was used to generate retroviruses following transfection with the CAR construct using

lipofectamine. Retroviral supernatant was collected 48 hours after transfection, and used for transduction of $CD8^+$ T cells in the presence of polybrene using spinfection (2000x g for 60 minutes at 37°C). Prior to transduction, $CD8^+$ T cells were purified with MojoSort murine $CD8^+$ T cell isolation kit (BioLegend Cat# 480007) and activated by plate-bound anti-CD3/CD28 in the presence of 10 ng/mL IL-7 and 10 ng/mL IL-15 for 24 hours. 16 hours after the transduction, the retroviral media was removed and the cells were transferred to uncoated 6-well plates and expanded in media containing 10 ng/mL IL-7 and IL-15 with PBS, mIL-21 (100 ng/ml) or 21h10 (100 ng/ml) for 3 days.

Serial tumor killing assay by CAR T cells using IncuCyte

MC38 adenocarcinoma tumor line was obtained from ATCC, and transduced using lentivirus to express human CD19 truncated (hCD19t) antigen and eGFP. The line was clonally selected and expanded for use in IncuCyte assay. For the Incucyte assay, MC38-CD19t eGFP cells were resuspended and plated at 25,000 cells per well in a clear flat bottom 96-well plate. 2 hours later, T-cells were added at 45,000 CAR-positive cells per well in triplicates. 25,000 MC38-CD19t eGFP cells tumor cells were added to the wells again at 36- and 68-hour time-points with half-volume media changes containing 21h10, mIL-21, and IL-7+IL-15. Tumor cell lysis was imaged in IncuCyte S3 every 2 hours and quantified by the loss of GFP signal (Mean Green Image). Acquisition time was set to 300 milliseconds, and Mean Green Image was used to evaluate cytotoxicity of all treatments.

Western blot analysis for the *in vivo* stability of 21h10

C57BL/6 mice were treated with 21h10 (n=12 mice), mIL-21 (n=12 mice), or PBS (n=4 mice) via intravenous tail vein injections. 1, 6, 9, and 24 hours following treatment, mice were bled and euthanized; spleens and lymph nodes were harvested and lysed in a buffer composed of 50 uM NaCl, 50 uM HEPES, 0.5% NP-40 with protease and phosphatase inhibitors in deionized water. A bicinchoninic acid assay was performed to determine the protein concentration of lysates. Equal amounts of spleen lysates were loaded in 4 to 20% SDS-polyacrylamide gels followed by a transfer to a polyvinylidene difluoride (PVDF) membrane using Bio-Rad Trans-blot Turbo Transfer System. Detection antibodies were incubated in 3% BSA in Tris-buffered saline with Tween 20 (TBS-T) composed of tris buffer, NaCl, and Tween 20. The antibodies used were: Phospho-Stat3 (9145S) and Stat3 (12640S). Once images of western blots were obtained, the relative density and size of bands were quantified on ImageJ.

Cancer cell lines and cell culture

Wild-type B16F10 melanoma cells (ATCC) were cultured in DMEM supplemented with 10% fetal bovine serum (Life Technologies Cat# 26140079), 1% GlutaMAX (Gibco Cat# 35050061), and 1% penicillin-streptomycin (Gibco Cat# 15140122). MC38 murine colon adenocarcinoma cells from James W. Hodge and Jeffrey Schlom's labs were cultured in DMEM supplemented with 10% fetal bovine serum (Life Technologies Cat# 26140079), 1% penicillin-streptomycin, 1% MEM NEAA (Gibco Cat# 11140050), 1% Sodium Pyruvate (Gibco Cat# 11360070), and 1% HEPES (Gibco Cat# 15630080). All cells were trypsinized and split every 3 days to avoid over-confluency and were maintained at 37°C in a humidified incubator with 5% CO_2. Cells used for *in vivo* experiments had been passaged for less than 2 months, negative for known murine pathogens, and were implanted at >95% viability.

In vivo MC38 adenocarcinoma cancer experiments

For the antitumor efficacy experiment in MC38 adenocarcinoma cancer, female C57BL/6 mice were inoculated subcutaneously with 500,000 MC38 adenocarcinoma cells. 5 days post-tumor inoculation, mice were dosed intraperitoneally with 21h10 (15 µg per mouse, n=5 mice), 21AT36 (13.9 µg per mouse, n=5 mice), anti-PD-1 (Bioxcell Cat# BE0146; 150 µg per mouse, n=5 mice), diluted in sterile endotoxin-free PBS, daily for two weeks. All mice were monitored for overall tumor growth and survival.

For the comparison experiment with mIL-21, C57BL/6 mice were inoculated subcutaneously with 500,000 MC38 adenocarcinoma cells. When tumors were palpable 6 days post-tumor inoculation, mice were dosed intraperitoneally with PBS (n=5 mice), 21h10 (15 µg per mouse, n=5 mice), 1x mIL-21 (14.9 µg per mouse, n=5 mice; R&D Systems Cat# 594-ML-100/CF), 10x mIL-21 (149 µg per mouse, n=5 mice), diluted in sterile endotoxin-free PBS, daily for 5 days. Tumors were measured every other day to monitor tumor growth.

For the rechallenge experiment, mice that had previously cleared their MC38 tumors after treatment with 21h10 (15 µg per mouse, n=56 mice) or anti-PD-1 (150 µg per mouse, n=8 mice) and were tumor-free for 30-90 days were used. Two days before and on the day of tumor inoculations, mice were treated with anti-CD4 (150 µg per mouse; BioXCell Cat# BE0003-1) and/or anti-CD8 depleting antibodies (150 µg per mouse; BioXCell Cat# BE0061) or rat IgG2b isotype (150 µg per mouse; clone LTF-2, BioXCell Cat# BE0090) diluted in sterile endotoxin-free PBS. Then, the tumor-free mice and naive mice were inoculated subcutaneously with new MC38 tumors, 1 million cells per mouse. Tumors were measured every other day to monitor tumor growth. Tumor measurements above 200 mm^3 were considered the endpoint.

To examine the role of anti-21h10 antibodies in antitumor activity, wild-type mice were treated daily with 21h10 (15 µg per mouse, n=38 mice) for 9 days and then taken off treatment for 9 days for 4 cycles, allowing mice to produce high levels of anti-21h10 antibodies. At the end of each treatment cycle, blood serum was collected from mice and used to measure anti-21h10 antibody levels via an ELISA (see methods outlined below). On the ELISA readings, samples were categorized as having low titer antibodies if their absorbance values were higher than two standard deviations above the mean values of the PBS samples; samples were categorized as having high titer antibodies if their absorbance values were higher than two times this value. Mice with varying anti-21h10 antibodies were inoculated subcutaneously with 500,000 MC38 adenocarcinoma cells. When tumors were palpable 6 days post-tumor inoculation, mice were dosed intraperitoneally with PBS (n=6 mice with anti-21h10 antibodies, n=5 naive mice) or 21h10 (15 µg per mouse, n=32 mice with anti-21h10 antibodies, n=15 naive mice) daily for 17 days. Tumors were measured every other day to monitor tumor growth.

To demonstrate the dependence of 21h10 on IL-21 signaling, wild-type and *Il21r*-/- mice (Jackson labs stock #019115) were inoculated subcutaneously with 1,000,000 MC38 adenocarcinoma cells. 6 days post-tumor inoculation, mice were dosed intraperitoneally with 21h10 (15 µg per mouse, n=5 wild type mice and n=4 *Il21r*-/- mice), 21AT36 (13.9 µg per mouse, n=5 wild type mice and n=4 *Il21r*-/- mice) , anti-PD-1 (150 µg per mouse, n=4 wild type mice and n=3 *Il21r*-/- mice), diluted in sterile endotoxin-free PBS, daily for two weeks. All mice were monitored for overall tumor growth and survival.

To examine the role of B cells in the antitumor activity of 21h10, wild type and µMT mice were inoculated subcutaneously with 1,000,000 MC38 adenocarcinoma cells. 5 days post tumor-inoculation, mice were dosed intraperitoneally with 21h10 (15 µg per mouse, n=5 wild type mice and n=5 µMT mice) or PBS daily for two weeks. All mice were monitored for overall tumor growth and survival.

In vivo adoptive cell transfer experiments

For comparison experiments with mIL-21, mice received $CD8^+$ T cells from $TRP1^{high}$ (Jackson labs stock #030958) and $TRP1^{low}$ (Jackson labs stock #030957) sex-matched donor mice via intravenous injection on day -1. The mice were inoculated subcutaneously with 500,000 wild-type B16F10 cells on day 0. Starting on day 5, mice were injected daily intraperitoneally with PBS (n=16 mice) or therapeutic doses of mIL-21 (14.9 µg per mouse; n=11 mice), 21h10 (15 µg per mouse, n=11 mice) diluted in sterile endotoxin-free PBS. Final treatments were given on day 25, and mice were monitored for overall tumor growth and survival. Mice with tumors that ulcerated before reaching a size of 500mm^3 were excluded from the dataset.

For the comparison experiment with Neo-2/15, half of the mice received $CD8^+$ T cells from $TRP1^{high}$ and $TRP1^{low}$ sex-matched donor mice via intravenous injection on day -1. All mice were inoculated subcutaneously with 500,000 wild-type B16F10 cells on day 0. Starting on day 5, all mice were injected daily with PBS (n=10 for each group with and without adoptive transfer) or therapeutic doses of Neo-2/15 (30 µg per mouse, n=10 for each group with and without adoptive T cell transfer) or 21h10 (15 µg per mouse, n=15 for group without adoptive transfer, n=13 for group with adoptive T cell transfer) diluted in sterile endotoxin-free PBS. Final treatments were given on day 21 and day 18 for mice that did and did not receive adoptive transfer of TRP1 cells, respectively. All mice were monitored for overall tumor growth and survival. Mice with tumors that ulcerated before reaching a size of 500mm^3 were excluded from the dataset.

For the comparison experiment with TNFα blockade, mice received $CD8^+$ T cells from $TRP1^{high}$ and $TRP1^{low}$ sex-matched donor mice via intravenous injection on day -1. The mice were inoculated subcutaneously with 500,000 wild-type B16F10 cells on day 0. Starting on day 5, mice were treated intraperitoneally with PBS (n=10 mice) or 21h10 (15 µg per mouse, n= 20 mice) daily and rat IgG2b isotype (150 µg per mouse,) or anti-TNFα (150 µg per mouse) every 3 days for 21 days. Tumors were measured every other day to monitor tumor growth.

In vivo tumor-infiltrating T cell analysis of Foxp3-GFP mice

On day 0, Foxp3-GFP mice (JAX Cat# 006772) received T cells from $TRP1^{high}$ and $TRP1^{low}$ CD45.1 donor mice and were inoculated subcutaneously with 500,000 wild-type B16F10 cells. Starting on day 5 after tumor cell inoculation, mice were injected daily (intraperitoneal) with therapeutic doses of Neo-2/15 (30 µg per mouse), 21h10 (15 µg per mouse), and mIL-21 (R&D Systems Cat# 594-ML) (14.9 µg per mouse). On day 14 post-inoculation, mice were treated with Brefeldin A (Sigma Aldrich Cat#B7651) intraperitoneally. 6 hours later, tumors were collected and homogenized into single-cell suspensions (and spleen briefly suspended in ACK lysis buffer) and cell populations were analyzed by flow cytometry using the following antibodies: Brilliant Violet 510 anti-CD4 (clone RM4-5, BioLegend Cat# 100559), Brilliant Violet 785 anti-CD8a (clone 53-6.7, BioLegend Cat# 100750), PE anti-CD45.1 (clone A20, BioLegend Cat# 110708), Brilliant Violet 711 anti-CD45.2 (clone 104, BioLegend Cat# 109847), Pacific Blue anti-CD11b (clone M1/70, BioLegend Cat# 101224), PE/Cyanine7 anti-PD-1 (clone RMP1-30, BioLegend Cat# 109109), and APC anti-TIM3 (clone RMT3-23, BioLegend Cat# 119706). All antibodies were used at a 1:100 dilution. Populations of adoptively transferred cells were distinguished by the congenic CD45.1 marker from the host's endogenous $CD45.2^{+}$ cells.

Intracellular cytokine staining of tumor-bearing mice

Additionally, tumor samples from above were analyzed using the following extracellular antibodies: Brilliant Violet 510 anti-CD4 (clone RM4-5, BioLegend Cat# 100559), Brilliant Violet 785 anti-CD8a (clone 53-6.7, BioLegend Cat# 100750), PE anti-CD45.1 (clone A20, BioLegend Cat# 110708), Brilliant Violet 711 anti-CD45.2 (clone 104, BioLegend Cat# 109847), Pacific Blue anti-CD11b (clone M1/70, BioLegend Cat# 101224), and also were processed with Intracellular Staining Permeabilization Wash Buffer (BioLegend Cat# 421002) and Fixation Buffer (BioLegend Cat# 420801) according to the manufacturer's instructions and stained with intracellular antibodies: Brilliant Violet 421 anti-IFN-γ (clone XMG1.2, BioLegend Cat# 505830), and Alexa Fluor anti-granzyme B (clone GB11, BioLegend Cat# 515405). Populations of adoptively transferred cells were distinguished by the congenic CD45.1 marker from the host's endogenous $CD45.2^{+}$ cells.

ELISA serum analysis for 21h10 specific anti-drug antibodies

Serum from mice treated daily with PBS, 21h10 (15 µg), and mIL-21 (14.9 µg) were tested for formation of 21h10 specific anti-drug antibodies. Mice were bled at the endpoint for each experiment. Individual whole blood samples were incubated for 5-10 minutes at 25°C, spun at 5,000 rpm for 5 minutes at 25°C, and the serum layer was collected. For the subsequent ELISAs, 96-well high-binding plates were coated overnight at 4°C with 21h10 protein (500 ng per well) diluted in 1X coating buffer (from ELISA set, Biolegend Cat#430804), and blocked with PBS + 10% IFS for 24 hours at 4°C. Serum samples diluted 1:100 in 1X Assay Diluent A (from ELISA set, Biolegend Cat# 430804) were plated and incubated for 2 hours at 25°C. Binding was detected with anti-mouse IgG HRP-linked antibody (Cell Signaling Cat# 7076) and incubated at 25°C for 1 hour and developed with 3,3′,5,5′-Tetramethylbenzidine (TMB) (Sigma Aldrich Cat# T0440). The reaction was stopped with 1N HCl (Fisher Scientific Cat# SA48500) after 5-10 minutes, and intensity measurement was read at 450 nm. Serum samples positive for anti-drug antibodies (above the dotted line) were determined to have an intensity measurement 2 SD above the mean absorbance value of samples from mice treated with PBS. Fractions indicate the number of mice classified as positive for anti-drug antibodies out of the total mice in each treatment group.

In vivo anti-TRP1 TA99 antibody experiment

For melanoma experiment with 21h10 and anti-TRP1 TA99 antibody, C57BL/6 mice were inoculated subcutaneously in the flank with 80-90% confluent wild-type B16F10 cells (500,000 cells per mouse) on day 0. Starting on day 3, mice were administered bi-weekly treatment with TA99 antibody intraperitoneally (150 µg per mouse). Starting on day 5, mice were injected daily with therapeutic doses of 21h10 (15 µg per mouse).

Ex vivo adoptive cell transfer

For *ex vivo* priming experiment with 21h10 and Neo-2/15, C57BL/6 mice were inoculated subcutaneously in the flank with 80-90% confluent wild-type B16F10 cells (500,000 cells per mouse) on day 0. On day 0, $CD8^{+}$ T cells were harvested from a $TRP1^{high}$ donor mouse and plated together with 21h10 (500 nM) or Neo-2/15 (500 nM) in RPMI complete medium in a 6-well plate that was pre-coated overnight at 4°C with anti-CD3 antibody (BioLegend Cat# 100340) at 100 pg/mL. After 2

days, cells were harvested and transferred by intravenous injection into the tumor-bearing mice. 10 mice were included in the PBS control group, 5 in the Neo-2/15-treated group, and 10 in the 21h10-treated group.

In vivo toxicity experiments

For testing treatment toxicity *in vivo*, C57BL/6, *Rag2-/-*, or *Rag2-/- Il2rg-/-* mice were treated with PBS or 21h10 (15 µg per mouse) daily and with rat IgG2b isotype (150 µg per mouse, clone LTF-2, BioXCell Cat# BE0090) or IgG2a anti-mouse NK1.1 depleting antibodies (150 µg per mouse, clone PK136, BioXCell Cat# BE0036) every three days. Treatment was continued for each mouse until weight loss reached 20% of the initial starting weight and mice were sacrificed. Serum from these mice was collected and analyzed by cytokine bead array (Evetechnologies). Cytokine values from WT mice were compared to *Rag2*-/- mice with a Wilcoxon rank test followed by P-value adjustment with the Benjamini-Hochberg (BH) procedure. Serum amylase activity was measured using Pointe Scientific Amylase Reagents according the to manufacturer's protocol (FisherScientific Cat#23-666-111). Serum creatinine was measured using an activity based Creatinine Assay Kit (Abcam Cat# ab65340). Serum transaminases were measured by ELISA using kits from Abcam (AST = ab263882-1x96Tests) (ALT = ab282882-1x96test). Pancreata were formalin-fixed, paraffin-embedded, and stained for hematoxylin and eosin by the Harvard Medical School Rodent Histopathology Core Facility.

Pancreatitis experiments

To examine the 21h10-induced pancreatitis, wild-type mice (n=45 mice) were treated daily with 21h10 (15 µg per mouse) or PBS and isotype or anti-TNFα every 3 days for 7, 14, or 21 days via intraperitoneal injections. On their last day of treatment, blood serum was collected from mice, mice were euthanized, and their pancreases were harvested; both pancreas and body weights were measured on the day of euthanization. Scale bars for zoomed out images are 1 mm 21h10-treated mice and 2 mm for PBS mice. The scale bar for 10x zoomed in images is 50 µm.

Amylase assay

Blood serum from the mice were collected twice a week (days 1, 4, 8, 11, 15, 18, 22, 25 of treatment). An amylase assay was conducted on these blood serum samples using the Pointe Liquid Amylase Reagent Set (A7564120). In short, in a 96-well plate, 100 uL of amylase was incubated at 37°C for five minutes, then, 2.5 µL of blood serum was added. After 1 minute, the enzymatic reaction was measured spectrophotometrically at 405 nm every minute for 3 minutes. To obtain the results in units per liter (U/L), the measured mean absorbance difference per minute was multiplied by the total assay volume and 1000 (conversion of U/mL to U/L) and then divided by the product of millimolar absorptivity of 2-chloro-p-nitrophenol (12.9), the sample volume, and the light path (1 cm).

Histology analysis for healthy organs

To examine the toxicity of 21h10 *in vivo*, wild-type mice were injected daily with 21h10 (15 µg per mouse, n=2 mice) for 25 days. On day 25, mice were euthanized, fixed in Z-Fix, a formalin-based buffer (NC9050753), and submitted to Harvard Medical School Rodent Histopathology Core to perform whole-body necropsy. Blocks of organs were sectioned to 5 microns and stained for H&E. Slides were scanned on the Olympus VS200 Slide Scanner using a 20x objective. The scale bar for the images is 50 µm.

TRP1 identification and T cell sub-clustering

From the custom reference built above, TRP1^{high} α/β and TRP1^{low} α/β TCR chains were counted by Cellranger. The sum of α and β chain expression for TRP1^{high} or TRP1^{low} TCRs was calculated for each cell. Cells were classified as TRP1^{high} or TRP1^{low} if they had an exclusive expression of one clonotype but not the other (e.g., high = TRP1^{high} > 0 & TRP1^{low} = 0). The few cells that had counts associated with both TCRs were not considered to be TRP1^{high} or TRP1^{low}. T cell sub-clustering was performed by subsetting cells expressing at least two CD3 chains (*Cd3d/e/g*). This initial T cell subset was split by sample and then new variable features were identified, with the exclusion of TCR variable genes (*Tr[abgd][vj]*), to reintegrate the samples back together. The top 30 PCs were used for UMAP and SNN graph generation. Six clusters separated from the main population, exemplified by above-average expressions of *Csf1r*, *Sirpa*, *H2-DMb2*, and *Lyz2*. These contaminating non-T cell clusters were removed from the initial T cell sub-clustering, and the process of splitting and reintegrating was repeated to

yield the final T cell sub-clusters, with the added step of regressing out ribosomal (*Rp[sl]*) and mitochondrial reads when scaling the data. Sub-clusters were identified as above with Louvain community detection and 'FindAllMarkers.' $CD4^+$ T cell sub-clustering was performed by selecting cells in the "CD4" and "Treg" sub-clusters or cells with the exclusive expression of *Cd4* (*Cd4* ≥ 1 and *Cd8a/b1* = 0). The same workflow of splitting, reintegrating (excluding TCR variable gene expression), and scaling while regressing out ribosomal/mitochondrial reads was performed. $CD4^+$ T cell sub-clusters were identified as above.

SUPPLEMENTARY FIGURES

Fig. S1

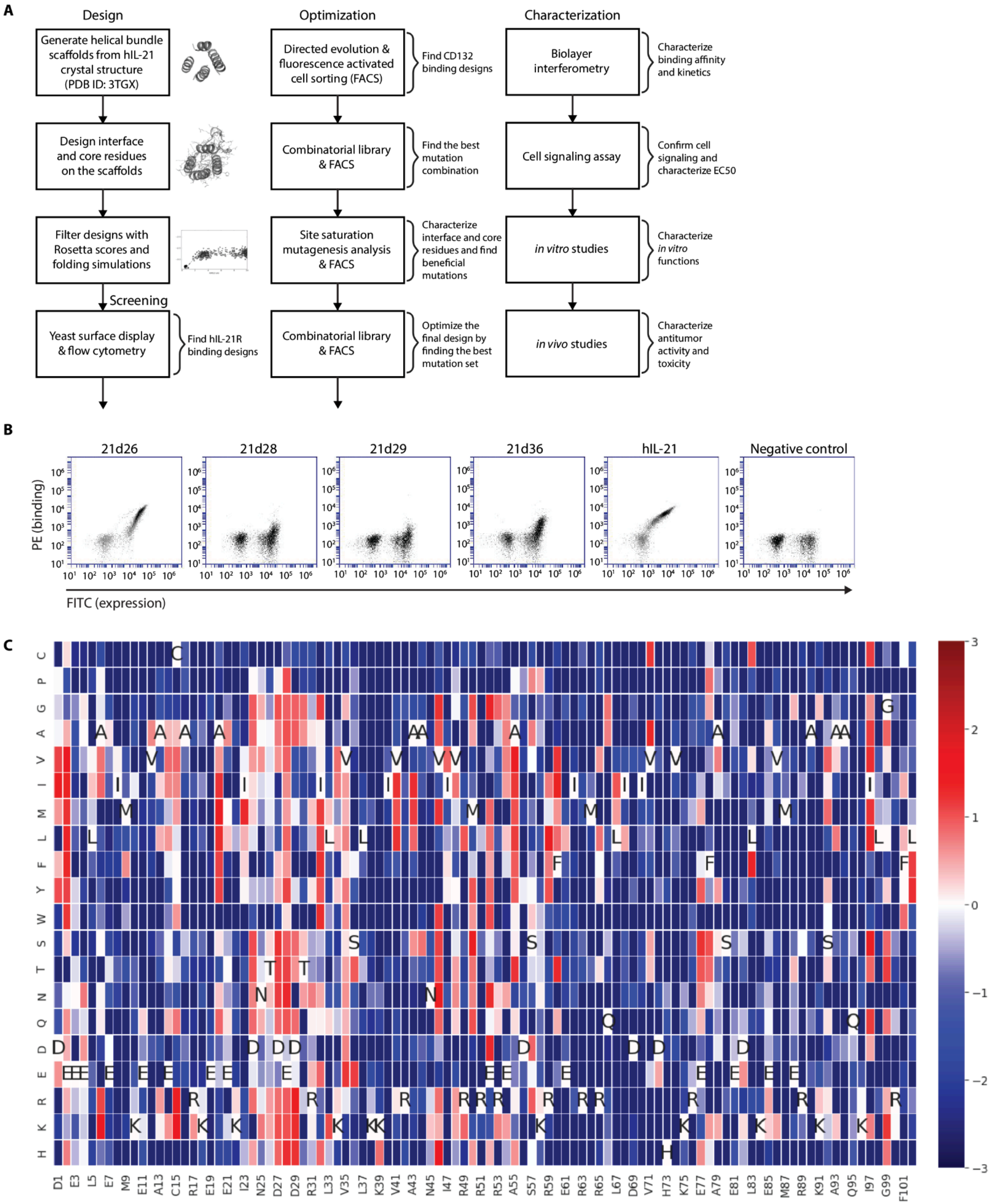

Fig. S1. Design, screening, and optimization of IL-21 mimic. (**A**) Stepwise process for designing, screening, optimizing, and characterizing IL-21 mimics. The helical bundle scaffolds are generated from the hIL-21 structure, and their residues are designed in the context of hIL-21R (PDB ID: 3TGX). The designs are computationally screened using *Rosetta* scores and folding simulations. The designs are experimentally screened using yeast surface display with flow cytometry. Optimization processes include directed evolution, SSM analysis, and combinatorial library screening. Further characterizations use BLI, cell signaling assay, and other *in vitro* and *in vivo* assays. (**B**) Four hits (21d26, 21d28, 21d29, and 21d36) from the initial designs were assessed for binding to hIL-21R at 100 nM. hIL-21 as positive control. (**C**) SSM result of 21JC15. Red indicates positive mutations, and blue indicates negative mutations for receptor complex binding. Scale in log10-fold change.

Fig. S2

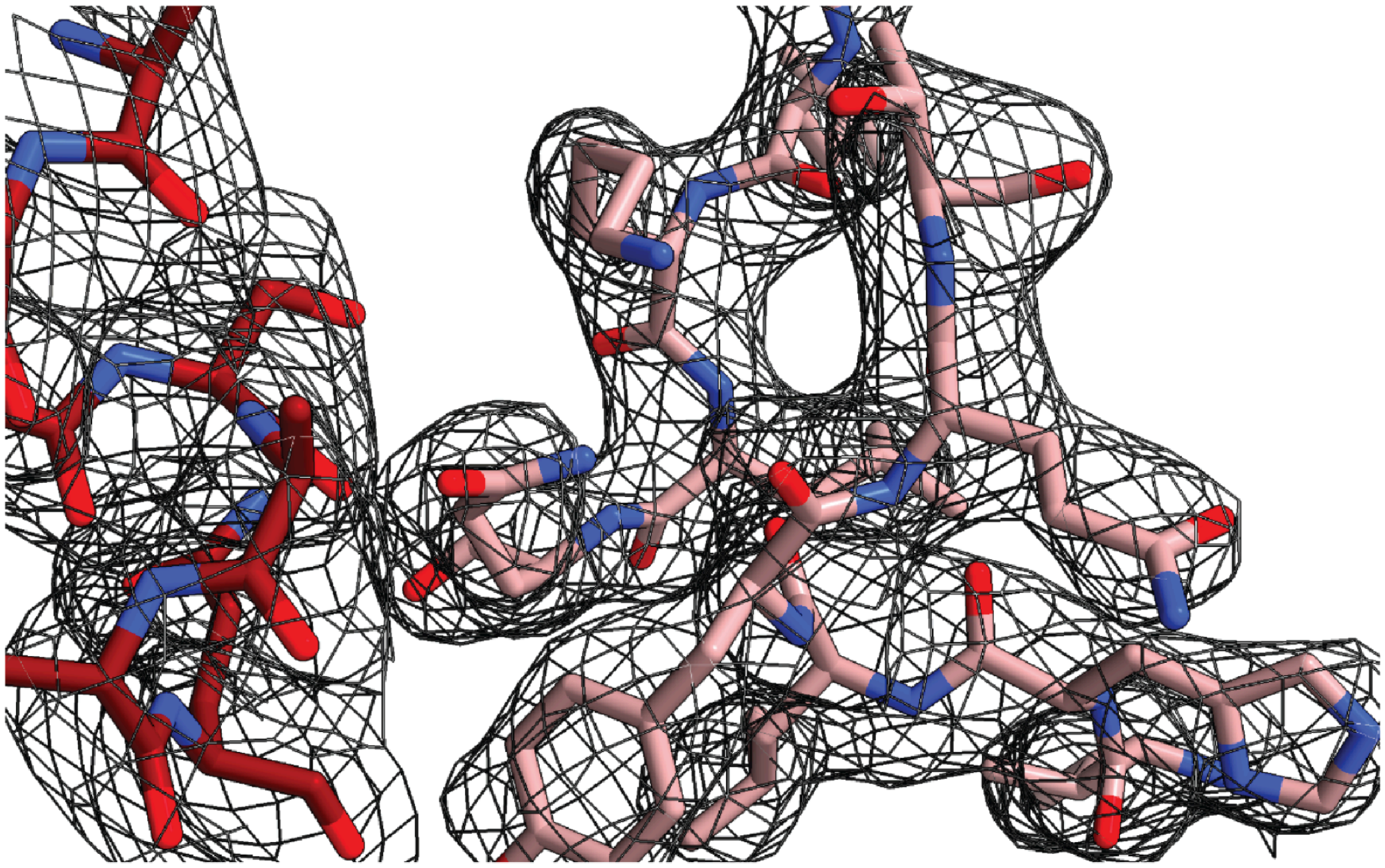

Fig. S2. Electron density of 21h10. Representative electron density. 2mFo-Dfc map (shown in gray) contoured at 1.0 σ. 21h10 is shown in red, hγ_c shown in light pink.

Fig. S3

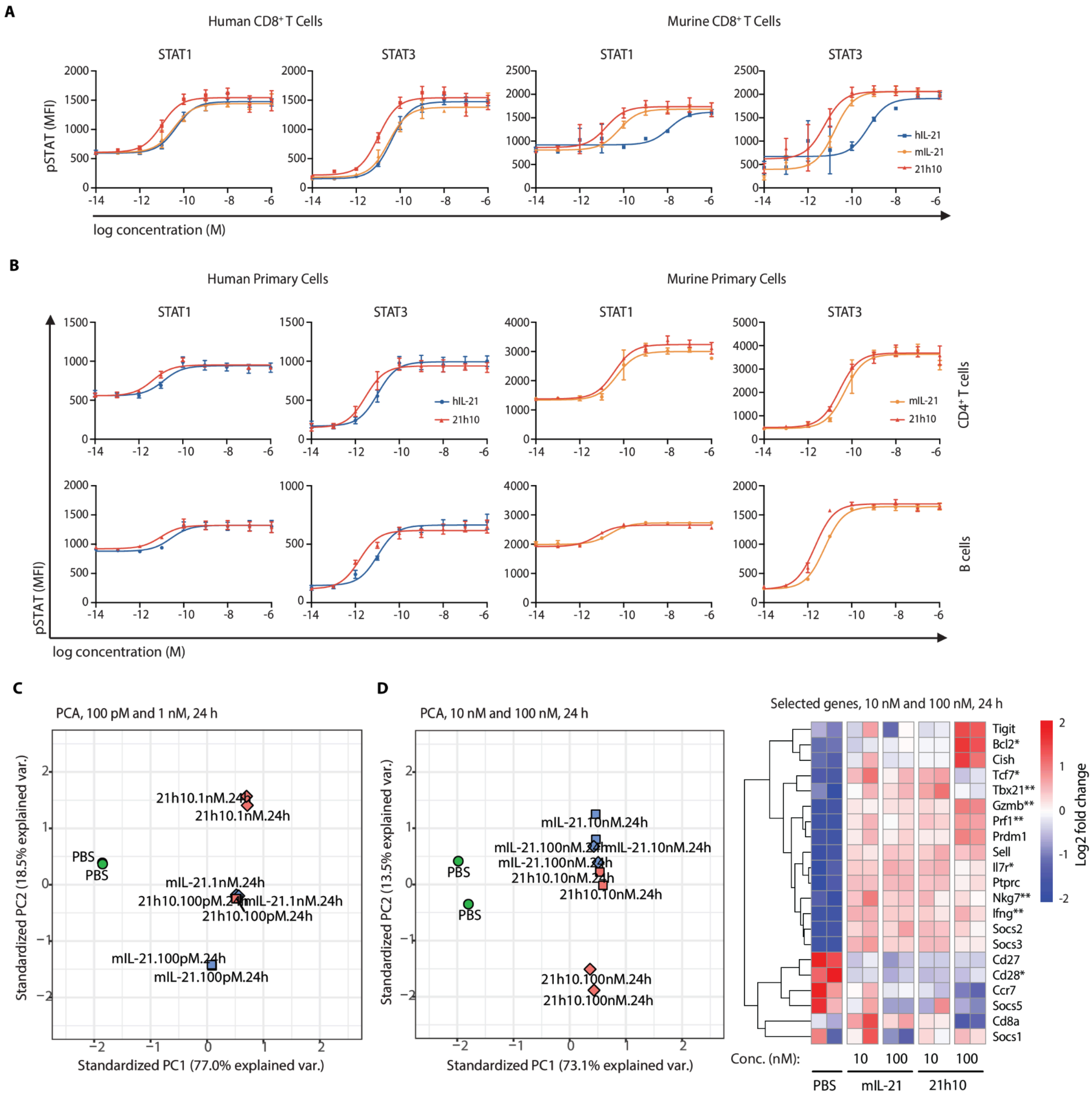

Fig. S3. STAT signaling of CD4⁺ and B cells and PCA analyses and gene expression comparison at higher concentrations of cytokines. (**A**) Cross-reactivity of hIL-21, mIL-21, and 21h10 in human and murine CD8⁺ T cells (n=3). (**B**) Phospho-STAT signaling of human and murine CD4⁺ T cells and B cells (n=3). (**C**) PCA analysis of RNA-seq data from murine CD8⁺ T cells treated with PBS, mIL-21, or 21h10 at 100 pM or 1 nM from Fig. 2B (n=2; biological duplicates are shown). (**D**) PCA analysis of RNA-seq data of murine CD8⁺ T cells treated with PBS, mIL-21, or 21h10 at 10 nM or 100 nM (n=2). A set of genes are compared for expression levels between the treatment groups. Scale in log2 fold change. * indicates memory-related genes, and ** indicates effector-related genes.

Fig. S4

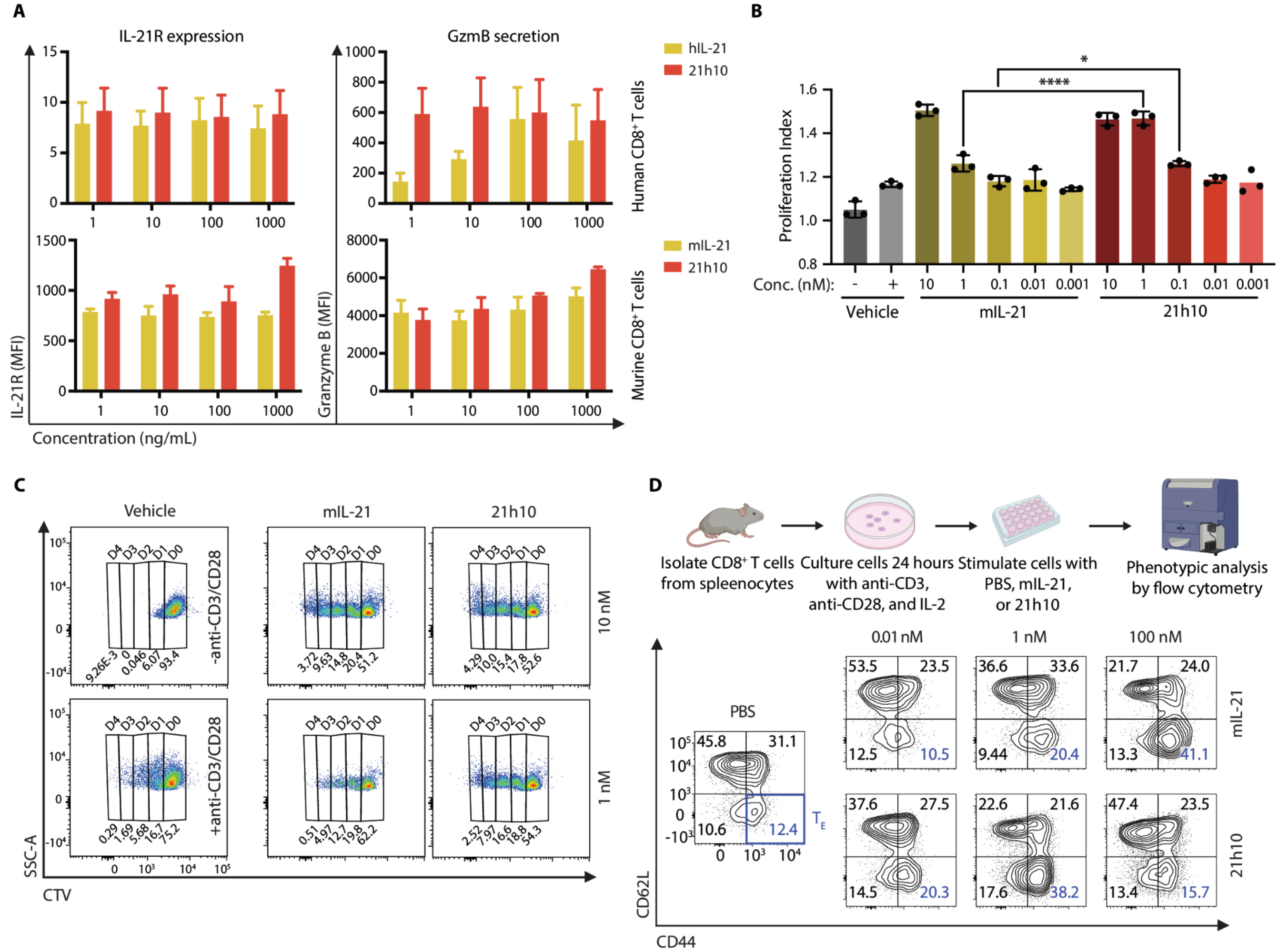

Fig. S4. Proliferation, differentiation, and expression of IL-21R and granzyme B of CD8$^+$ T cells in the presence of mIL-21 or 21h10. (**A**) IL-21R and granzyme B expressions of human and murine CD8$^+$ T cells were measured by flow cytometry after culturing with anti-CD3, anti-CD28, and corresponding cytokines for 72 hours. Significance was determined using two-way ANOVA with Tukey's correction. (**B**) Murine TRP1^{high} CD8$^+$ T cell proliferation was quantified using flow cytometry with Cell Trace Violet (CTV). All groups were treated for 72 hours with anti-CD3/CD28 beads along with various amounts of the cytokines that were incubated prior to addition onto the cells at 37°C for 24 hours in human serum. Significance was determined using one-way ANOVA with Šidák's correction. Data are representative of three experimental replicates. (**C**) Flow cytometry plots of CTV-labeled cells from (**B**). (**D**) Murine CD8$^+$ T cells were isolated and then activated with anti-CD3/CD28 antibodies and IL-2 for 24 hours. Cells were then incubated with PBS, mIL-21, or 21h10 at the indicated concentrations for 48 hours. The cells were analyzed for their differentiation into the effector T cell (CD44$^+$CD62L$^-$) population using flow cytometry. Figure created with BioRender.

Fig. S5

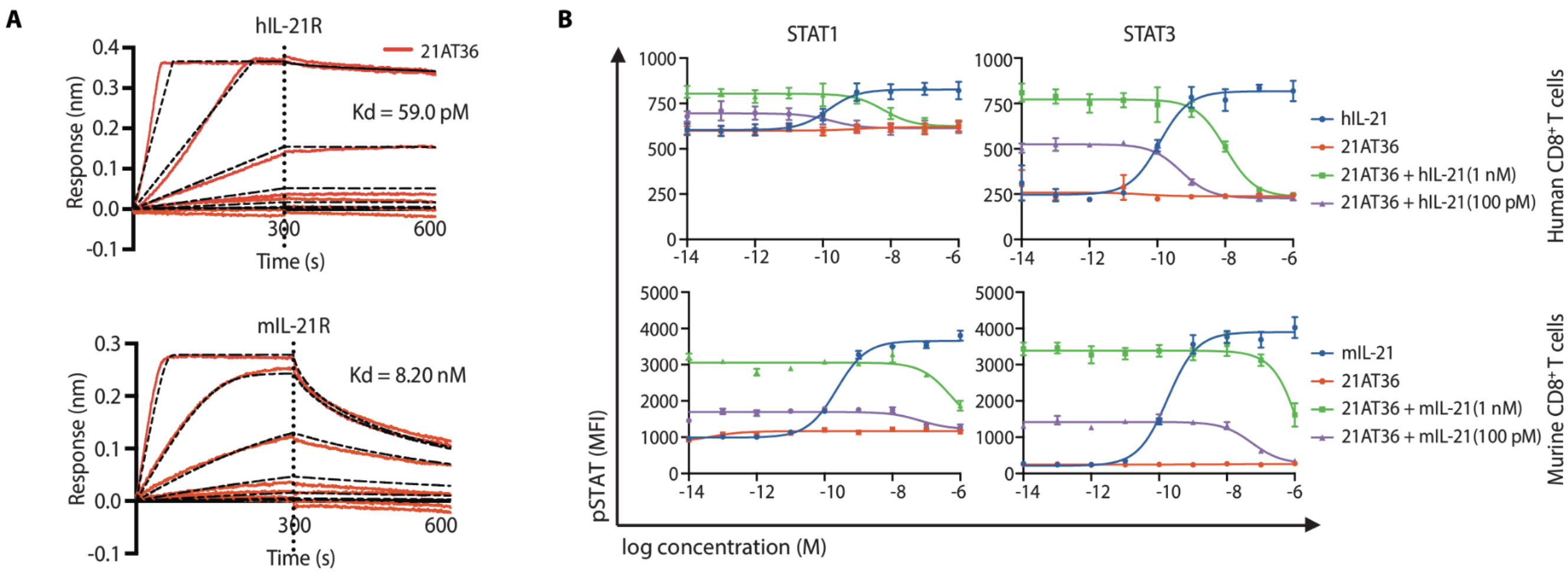

Fig. S5. Antagonist, 21AT36, binds to IL-21R but does not signal through STAT. (**A**) Biolayer interferometry against hIL-21R and mIL-21R. Black dotted lines show curve fits of raw data using the Mass Transport model. (**B**) Competition and inhibition of STAT phosphorylation by 21AT36 was measured with or without the presence of native IL-21

Fig. S6

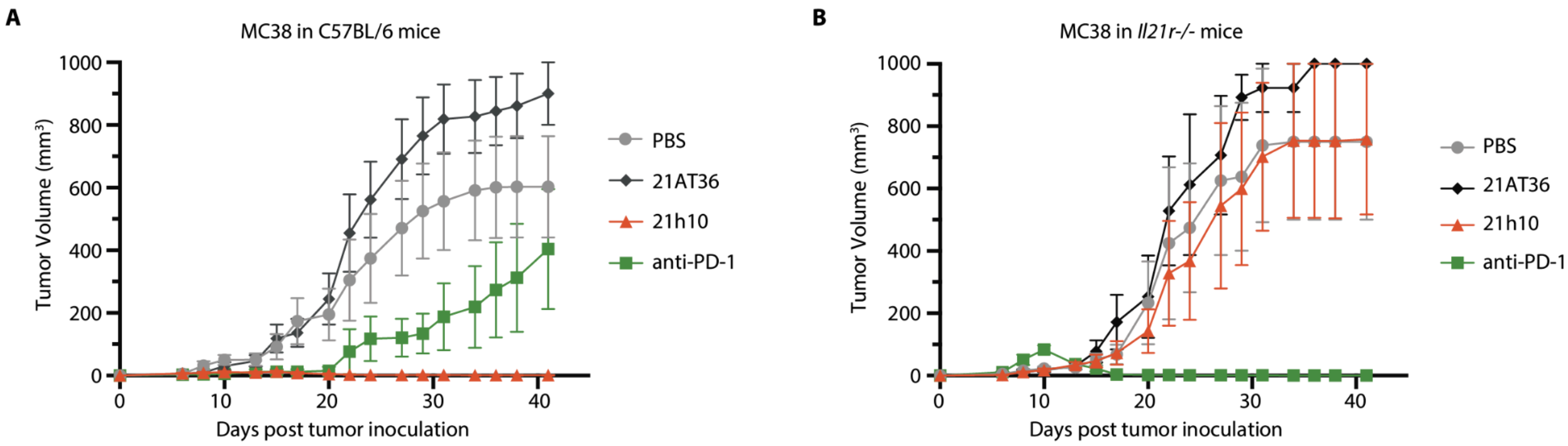

Fig. S6. 21h10 shows antitumor activity against MC38 adenocarcinoma comparable to anti-PD1 and requires signaling through the IL-21 receptor. (**A**) Antitumor activity of 21h10, 21AT36, and anti-PD-1 in murine MC38 in C57BL/6 mice, and (**B**) in *Il21r-/-* mice. Data are representative of two experimental replicates.

Fig. S7

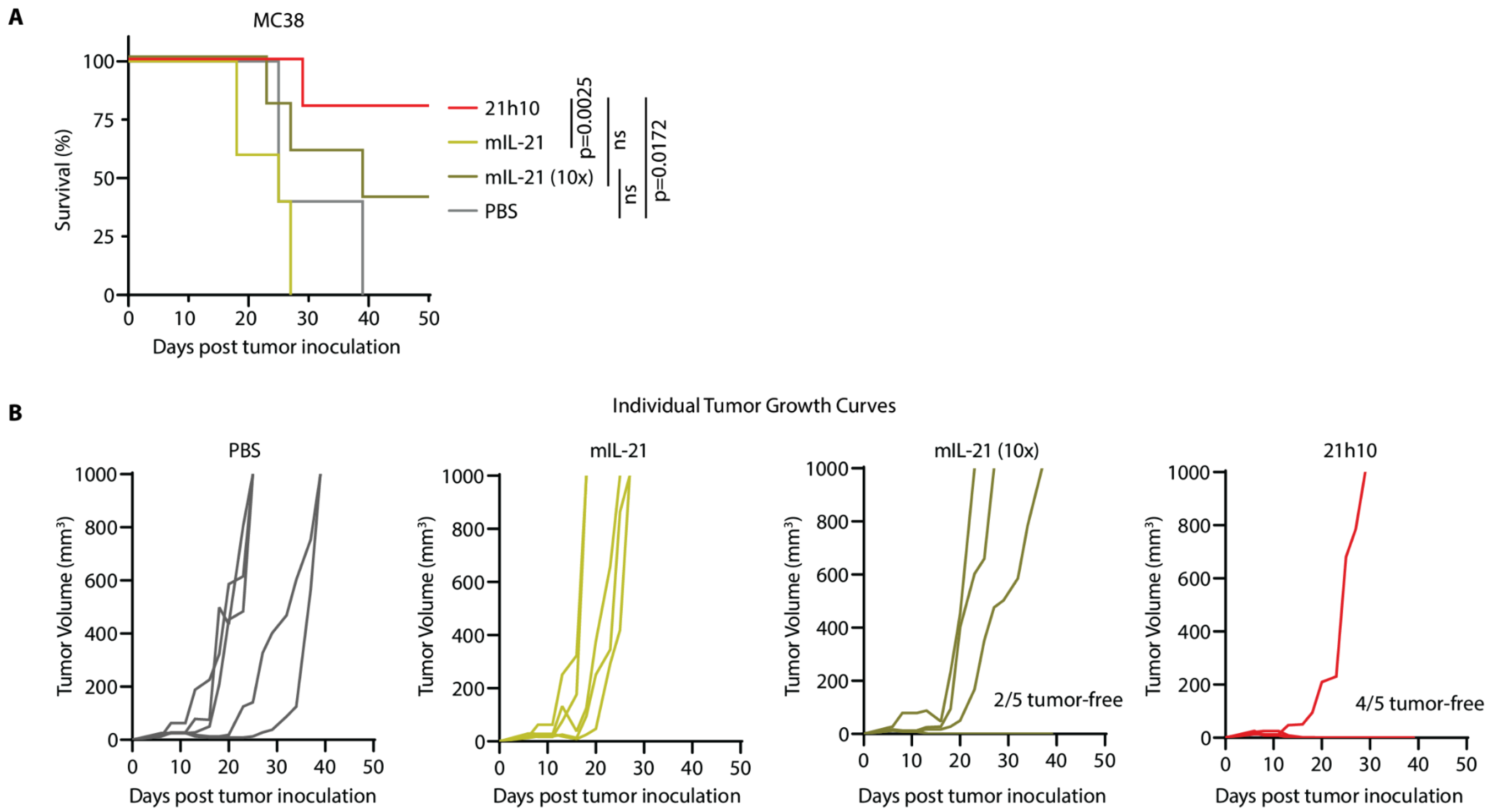

Fig. S7. Survival and individual tumor growth curves from Fig. 2H. (**A**) Survival curve and (**B**) individual tumor growth curves from Fig. 2H. Significance was determined in Kaplan-Meier survival curves using Mantel-Cox log-rank tests.

Fig. S8

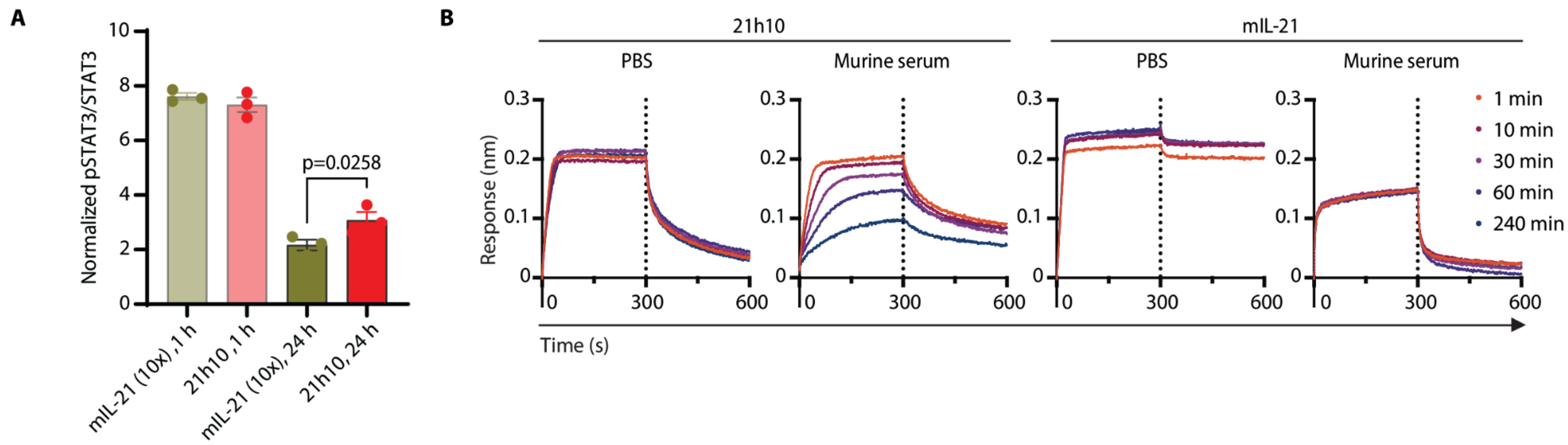

Fig. S8. 21h10 shows enhanced serum stability than mIL-21. (**A**) C57BL/6 mice were injected intravenously with a single dose of 21h10 or 10-fold molar excess of native mIL-21. Lymph nodes were harvested 1 or 24 hours later and analysed by immunoblot for phosphorylated STAT3. Significance was determined using one-way ANOVA with Šidák's correction. This experiment was performed once. (**B**) BLI of mIL-21 and 21h10 to quantify the amount of protein binding to mIL-21R in PBS or murine serum for serum stability comparison. mIL-21 and 21h10 were incubated in PBS or murine serum for 1-240 minutes at 37°C. PBS incubation shows maximal binding possible with the given proteins.

Fig. S9

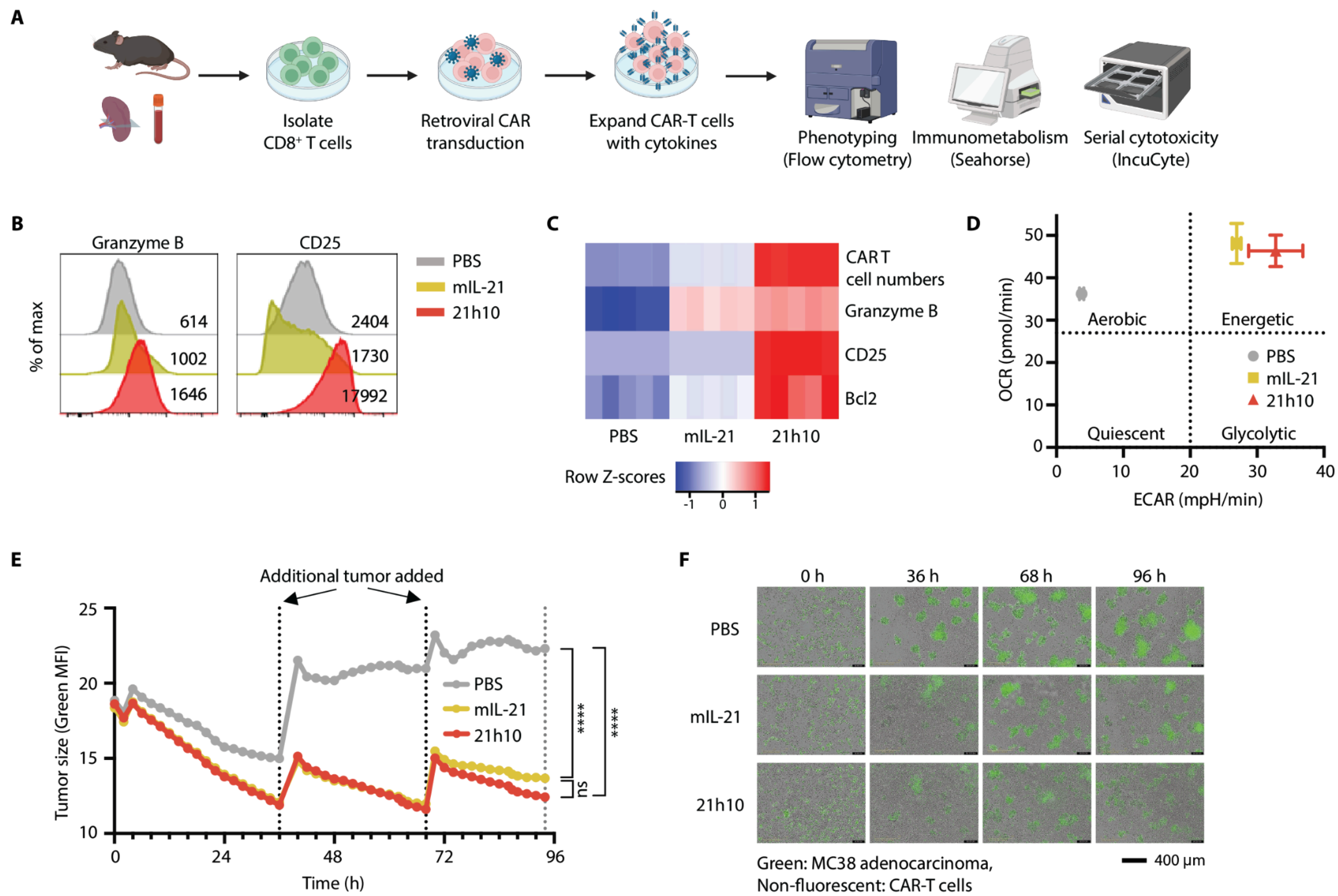

Fig. S9. 21h10 enhances cytotoxicity of CAR-T cells and increases their serial tumor-killing capability against MC38 adenocarcinoma. (**A**) $CD8^+$ T cells were isolated and activated using anti-CD3/CD28, followed by transduction with a retroviral vector expressing CD19-targeting CAR to generate anti-CD19 CAR-T cells. Figure created with BioRender. (**B**) Granzyme B and CD25 expression in CAR-T cells treated with PBS, mIL-21, or 21h10 are measured using flow cytometry. Numbers in the histogram plots indicate the mean fluorescence intensity (MFI) of marker expression. (**C**) MFI of CAR expression, cell numbers, and MFI of granzyme B, CD25, and Bcl2 levels between PBS-, mIL-21-, and 21h10-treated CAR-T cells are presented as heat maps. Z-scores were calculated on a row-by-row basis by subtracting the mean from the datapoint and dividing by the standard deviation. (**D**) Metabolic profiles of PBS-, mIL-21-, and 21h10-treated CAR-T cells show potent induction of oxygen consumption rate (OCR) and extracellular acidification rate (ECAR) in Seahorse assay by 21h10. (**E**) Serial tumor killing of $CD19^+$ GFP-MC38 adenocarcinoma by anti-CD19 CAR-T cells treated with PBS, mIL-21, or 21h10. Significance was determined using one-way ANOVA with Tukey's correction. (**F**) IncuCyte images of serial tumor killing assay in (**E**).

Fig. S10

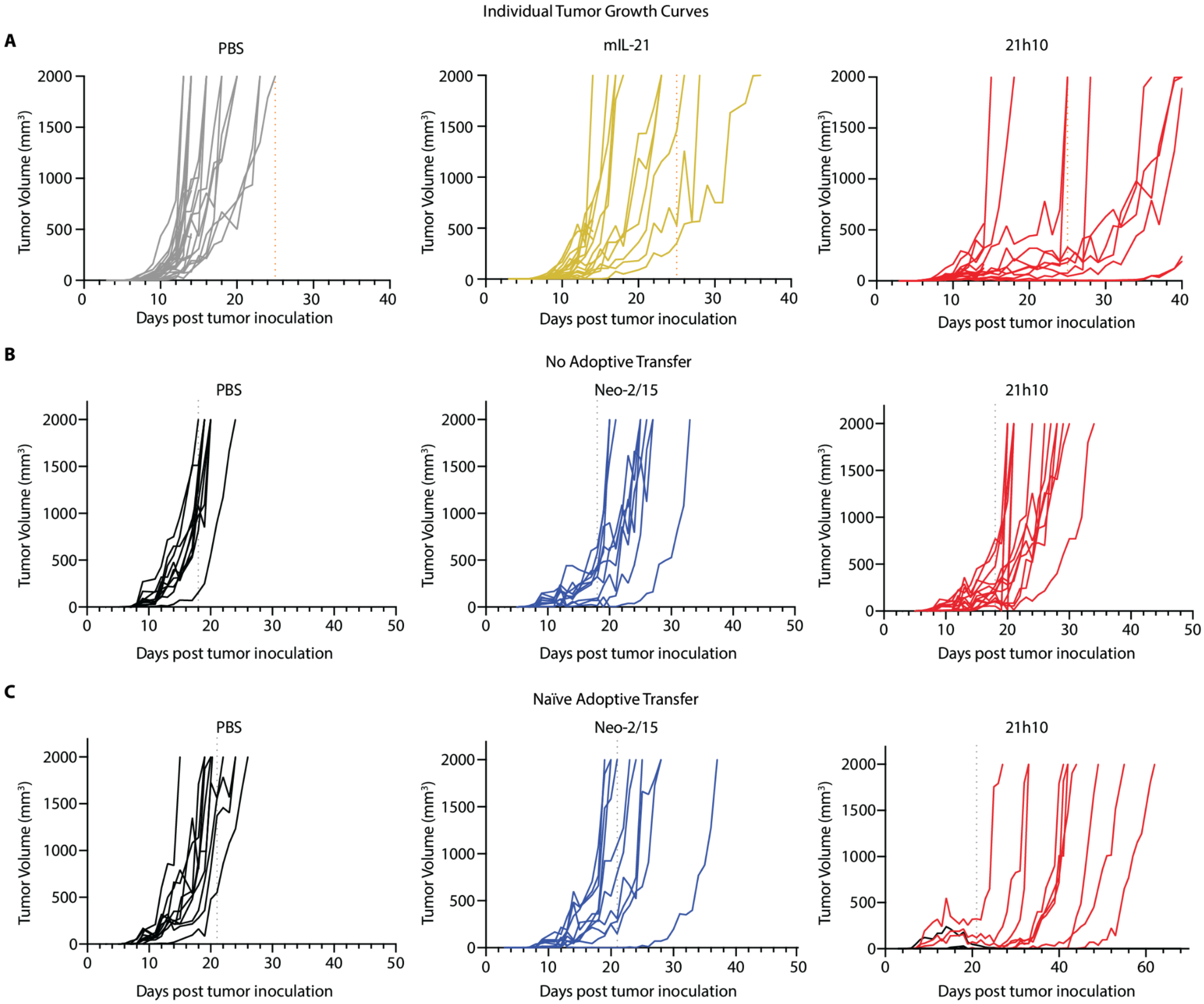

Fig. S10. Survival and individual tumor growth curves from Fig. 2H and Fig. 3. (**A**) Individual tumor growth curves from Fig. 3A. (**B**) Individual tumor growth curves from Fig. 3B. (**C**) Individual tumor growth curves from Fig. 3C.

Fig. S11

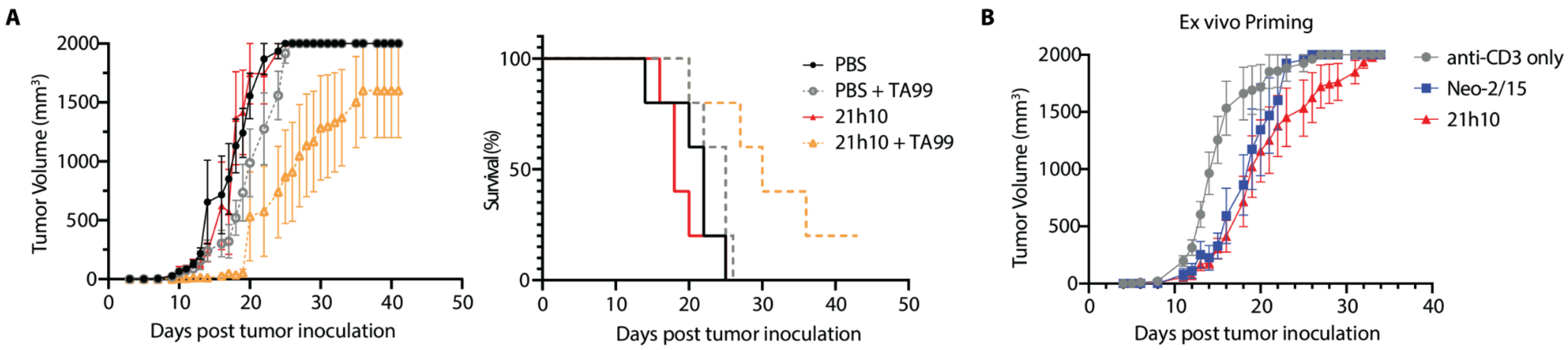

Fig. S11. 21h10 improves antibody therapy and *ex vivo*-primed T cell therapy. (**A**) Mice received B16F10 tumors and treatment with PBS or 21h10. Treatments were done alone or combined with TA99 (anti-TRP1 antibody). (**B**) TRP1 melanoma-specific T cells were primed *ex vivo* using anti-CD3 alone or in combination with Neo-2/15 or 21h10 for 2 days. Primed cells were transferred to mice 2 days after receiving B16F10 tumors.

Fig. S12

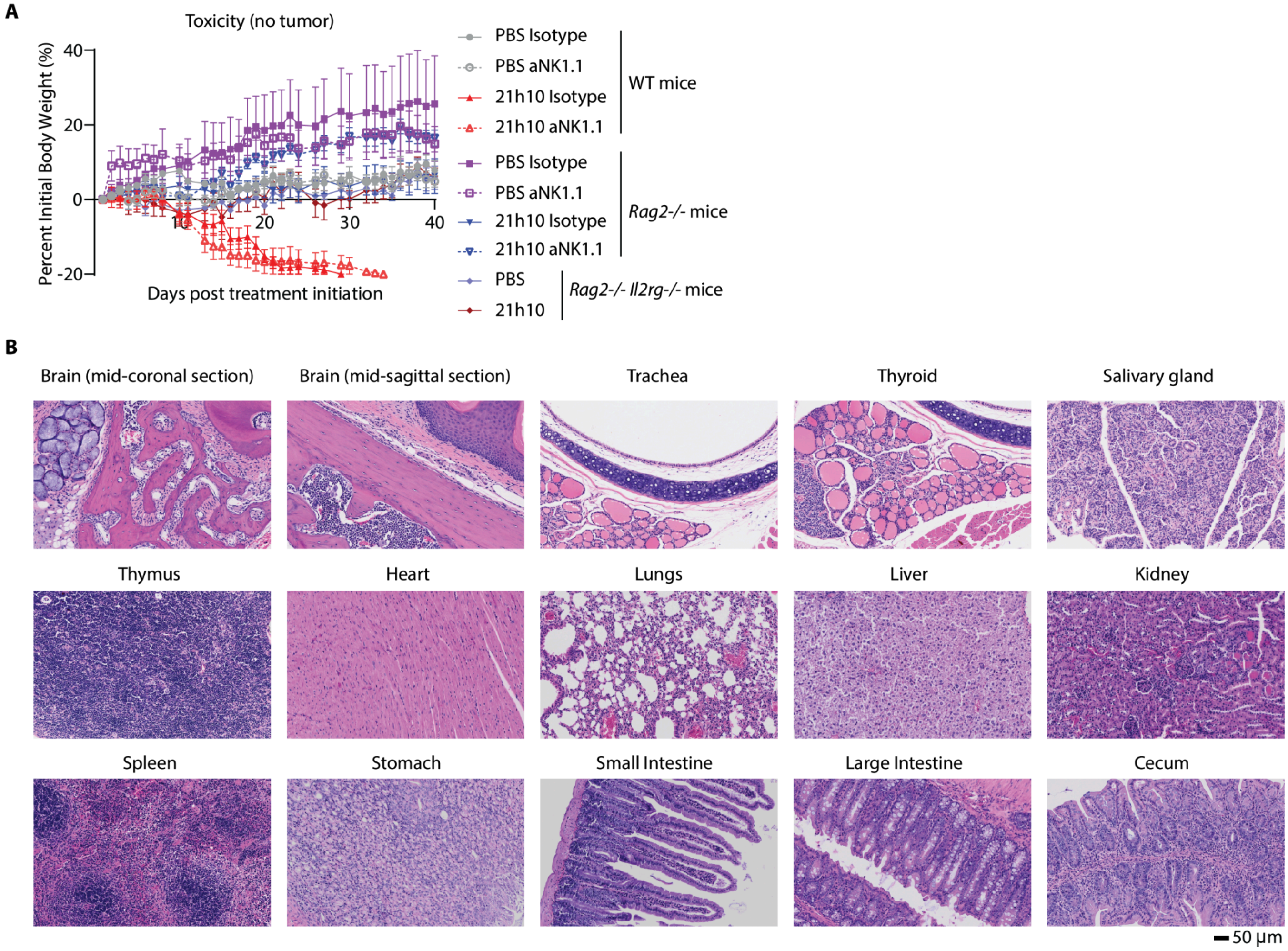

Fig. S12. Toxicity profiling of 21h10 treatment. (**A**) Wild-type (WT), *Rag2-/-*, or *Rag2-/- Il2rg-/-* mice were treated with PBS or 21h10 daily and isotype or anti-NK1.1 depleting antibodies every three days. Mice were sacrificed when weight loss exceeded 20% of the initial starting weight. (**B**) Immunohistochemistry (IHC) staining of tissues other than pancreas from 21h10-treated WT mice. Data are representative of two experiments.

Fig. S13

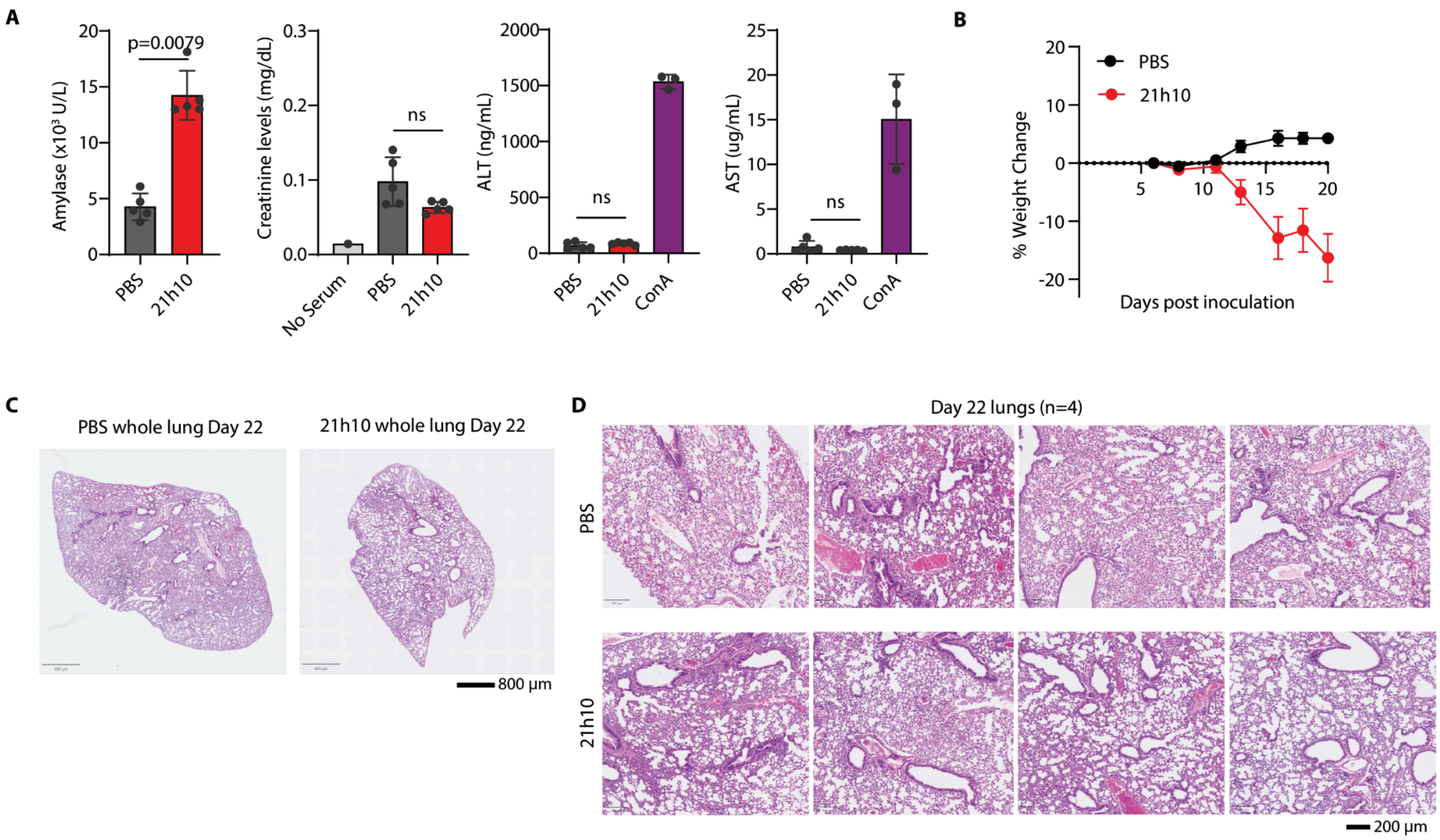

Fig. S13. Pancreas, liver toxicity profiling and lung histology with 21h10 treatment. (**A**) Amylase, creatinine, ALT, and AST levels are measured from serum. Significance was determined using Mann-Whitney U test. (**B**) Body weight change was tracked in PBS- and 21h10-treated groups. Data are representative of more than five experimental replicates. (**C**) H&E staining of lung from PBS- and 21h10-treated WT mice at day 22. Scale = 800 µm. (**D**) Zoomed-in image of lung histology. Scale = 200 µm.

Fig. S14

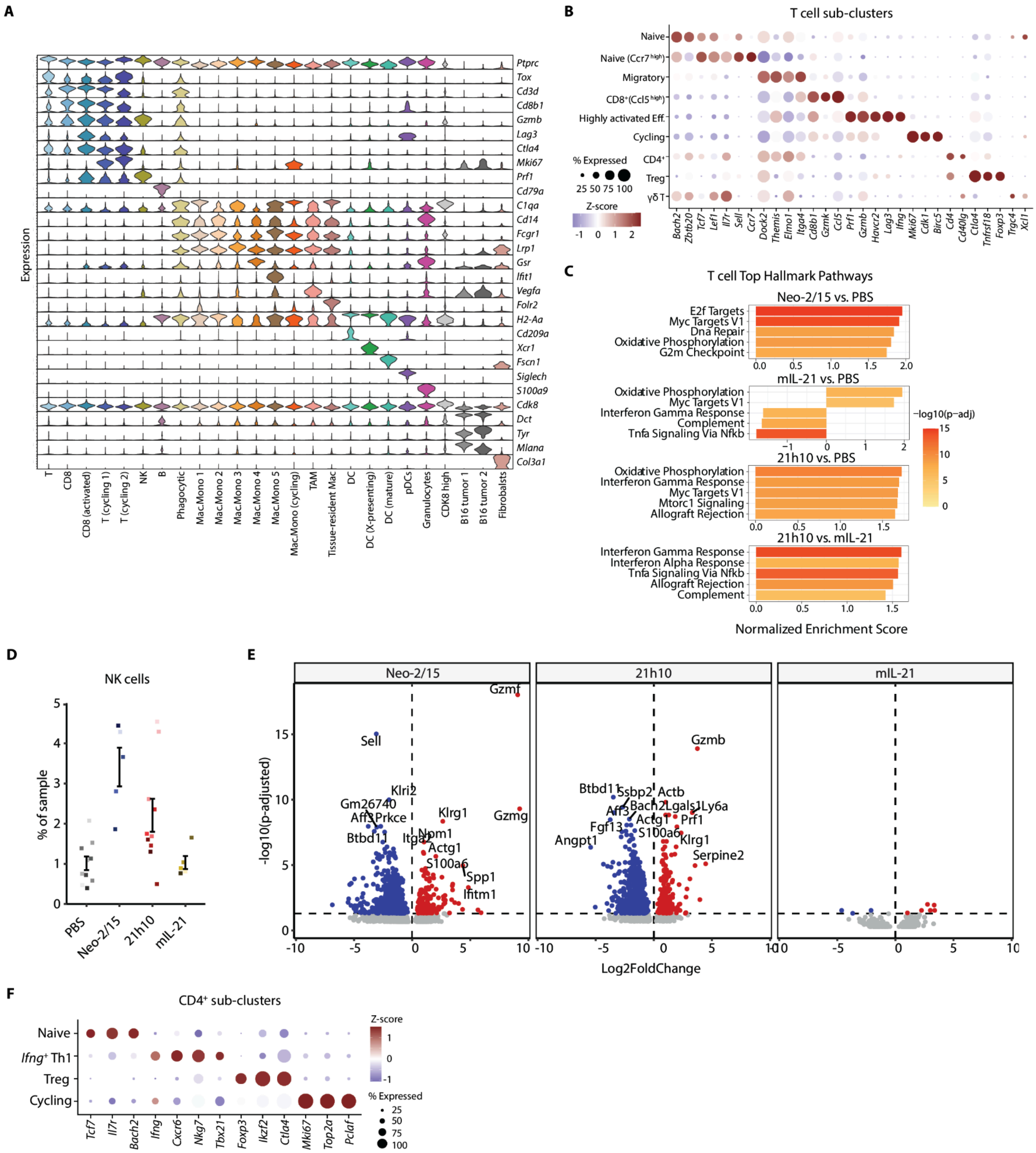

Fig. S14. Cluster-defining genes for single-cell RNA-sequencing. (**A**) Violin plot of canonical gene expression for each cluster identified from Fig. 6A. (**B**,) Dot plot of cluster-defining genes for T cell sub-clusters from Fig. 6F. (**C**) Hallmark Gene Set Enrichment Analysis within T cells for given comparisons. Significance calculated using the *fgsea* R package. (**D**) Percentage of NK cell population in samples from PBS, Neo-2/15, 21h10, and mIL-21 groups. (**E**) Volcano plots of gene expression in NK cells from Neo-2/15, 21h10, and mIL-21 groups. *DESeq2* was used to identify differentially expressed genes from scRNA-seq using the Wald test. (**F**) Dot plot of cluster-defining genes for CD4^{+} T cell sub-clusters from Fig. 6K.

Fig. S15

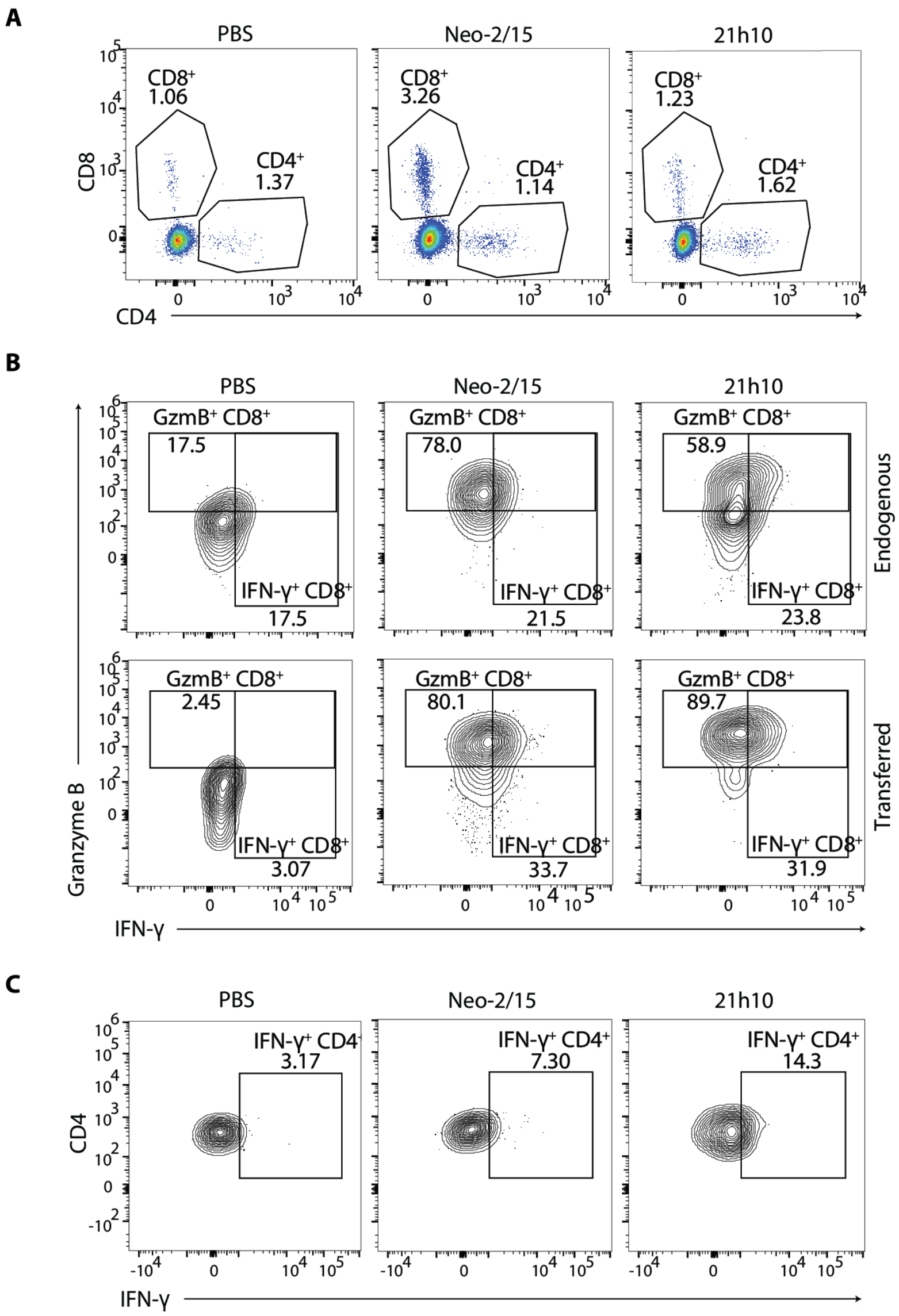

Fig. S15. Representative flow cytometry plots. Representative flow cytometry plots from (**A**) Fig. 6D, (**B**) Fig. 6J, or (**C**) Fig. 6L. Data are representative of two experiments.

Fig. S16

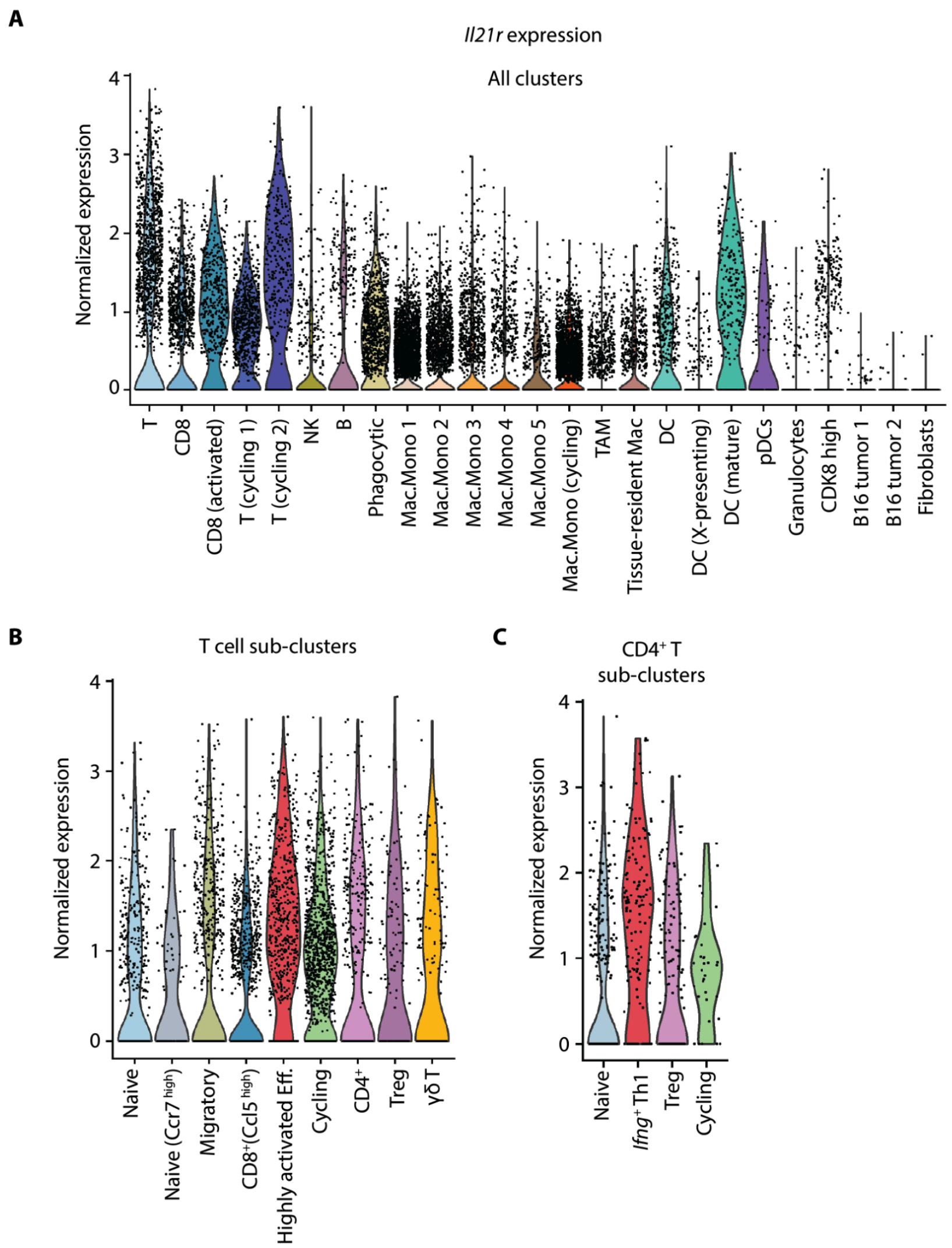

Fig. S16. *Il21r* expression across clusters. (**A**) Violin plots of normalized *Il21r* expression for total clusters, (**B**) T cell sub-clusters, or (**C**) CD4$^+$ T cell sub-clusters.

SUPPLEMENTARY TABLES

Table S1. Amino acid sequences of IL-21 mimics.

Name	Sequence
**21d26**	DEEELAEIMKEVAEWARKEAEKIDNTDEDTRILKVSLKKIVRAANVIVWMREREADSKRFEIRMRQLIDIVDQVKREFASEDLKEVMERA KSAAQKILHRFL
**21d28**	RDEIRARQLIDIVDQMKRVAEKNNMDRRMKEVLERAKSAAQKILHALLRNLELREIMEEFIEAVEEVVEFWKNLSSTSEKELRIMEVSLKK LIRAALVLKLR
**21d29**	RDEIRARQLIDIVDQMKRYAEKNNMDRRMKEVLERAKSAAQKILHALLRNLELREIMEEFIEAVEEVVEFWKNLSSTSEKELRIMEVSLKK LIRAALVLKLR
**21d36**	SRIWEVSAKKLIRAATVAKYIQDSEADSKRAAIRLRQLIDIADQVKKEDADDKWKEILERFKSAAQKALHAWLDGDRRDKREMRKAAEE LIKAAEEIAREK
**21JC15**	DEEELAEIMKEVAECARKEAEKIDNTDEDTRILKVSLKKIVRAANVIVRMREREADSKRFEIRMRQLIDIVDHVKREFASEDLKEVMERAK SAAQKILGRFL
**21h10**	DEEELKEIMKEAAECARKELEKLDNTDEDTRWLKITLKKIVRMANRIVRMREREADSKRFEIRMRGLIDIADHVKREFASEDLKEVMERA KSAAQKALGRWL
**21AT36**	DEEELKEIMKEAAECARKELEKLDNTDEDTRWLKITLKKIVRMANRIVRMREREADSKRFEIRMRGLIDIADHVKREFASEDLKEVMERA AKAAEEALRRWL

Table S2. K_D values of biolayer interferometry (BLI) assays.

EC_{50} (M)	Receptor	Kinetic fit values				
		K_D (Error) [M]	k_{on} (Error) [$M^{-1}s^{-1}$]	k_{off} (Error) [s^{-1}]	Full X^2	Full R^2
**21h10**	**hIL-21R**	7.37E-11 (1.27E-05)	2.28E+14 (2.79E+19)	1.68E+04 (2.06E+09)	2.276	0.9791
	**hIL-21R/hγ_c**	8.55E-08 (4.00E-09)	1.40E+05 (4.73E+03)	1.19E-02 (3.85E-04)	0.2135	0.9913
	**mIL-21R**	5.17E-09 (2.42E-10)	2.39E+06 (7.96E+04)	1.24E-02 (4.08E-04)	0.1555	0.9948
	**mIL-21R/mγ_c**	9.33E-08 (1.08E-08)	3.91E+05 (3.25E+04)	3.64E-02 (2.95E-03)	0.197	0.9622
**21AT36**	**hIL-21R**	5.90E-11 (2.45E-10)	1.18E+08 (3.53E+08)	6.95E-03 (2.01E-02)	0.4388	0.9936
	**mIL-21R**	8.20E-09 (5.76E-10)	1.94E+06 (9.89E+04)	1.59E-02 (7.72E-04)	0.087	0.9951

Table S3. Crystallography data collection and refinement statistics.

**Wavelength**	0.97946
**Resolution range**	39.7 - 2.281 (2.362 - 2.281)
**Ellipsoidal[a] Resolution Limit[b]**	2.279 (0.951 a* + 0.298 b* - 0.085 c*); 3.416 (-0.539 a* + 0.832 b* + 0.130 c*); 2.475 (-0.089 a* - 0.139 b* + 0.986 c*)
**Space group**	P 1
**Unit cell**	75.499 78.082 101.219 94.63 103.395 114.554
**Total reflections**	481809 (48857)
**Unique reflections**	84418 (212)
**Multiplicity**	5.7 (5.7)
**Completeness (%)**	52.87 (2.31)
**Completeness (ellipsoidal)[c] (%)**	85.62 (59.55)
**Mean I/sigma(I)**	5.17 (0.24)
**Mean I/sigma(I) (ellipsoidal)[c]**	8.71 (1.32)
**Wilson B-factor**	50.55
**R-merge**	0.2401 (8.247)
**R-meas**	0.2643 (9.069)
**R-pim**	0.1101 (3.758)
**CC1/2**	0.995 (0.0744)
**Reflections used in refinement**	48278
**Reflections used for R-free**	1929
**R-work**	0.1976
**R-free**	0.2426
**Number of non-hydrogen atoms**	13246
**macromolecules**	13053
**ligands**	162
**solvent**	31
**Protein residues**	1590
**RMS(bonds)**	0.006
**RMS(angles)**	0.74
**Ramachandran favored (%)**	95.55
**Ramachandran outliers (%)**	0.13
**Rotamer outliers (%)**	2.68
**Clashscore**	5.78
**Average B-factor**	59.55
**macromolecules**	59.61
**ligands**	59.6
**solvent**	34.47
**Number of TLS groups**	10

Statistics for the highest-resolution shell are shown in parentheses.
[a]These statistics are for data that were truncated by STARANISO to remove poorly measured reflections affected by anisotropy.
[b]The resolution limits for three directions in reciprocal space are indicated here. To accomplish this, STARANISO computed an ellipsoid postfitted by least squares to the cutoff surface, removing points where the fit was poor. Note that the cutoff surface is unlikely to be perfectly ellipsoidal, so this is only an estimate.
[c]The anisotropic completeness was obtained by least squares fitting an ellipsoid to the reciprocal lattice points at the cutoff surface defined by a local mean I/σI threshold of 1.0, rejecting outliers in the fit due to spurious deviations (including any cusp), and calculating the fraction of observed data lying inside the ellipsoid so defined. Note that the cutoff surface is unlikely to be perfectly ellipsoidal, so this is only an estimate.

Table S4. Key contacts in the 21h10 receptor complex.

Interface overview			
Interaction	Structure 1	Structure 2	Interface [Å^2]
Site I	21h10	hIL-21R	870.7
Site IIa	21h10	hγ_c	423.1
Site IIb	hIL-21R	hγ_c	693.8

Site IIa 21h10-hγ_c interface			
Hydrogen bonds	hγ_c	Distance [Å]	21h10
1	C:GLN149[OE1]	3.08	A:SER92[OG]
2	C:GLN149[OE1]	3.10	A:GLN95[NE2]

Site I 21h10-hIL-21R interface			
Hydrogen bonds	hIL-21R	Distance [Å]	21h10
1	B:ASP72[O]	2.78	A:ARG59[NH2]
2	B:ASP72[OD2]	2.91	A:ARG63[NH1]
3	B:ASP72[OD2]	3.15	A:ARG46[NH1]
4	B:MET130[SD]	3.25	A:LYS39[NZ]
5	B:GLY188[O]	3.28	A:ARG65[NH2]

Site IIb hIL-21R-hγ_c interface			
Hydrogen bonds	hγ_c	Distance [Å]	hIL-21R
1	C:ARG205[NH1]	2.84	B:GLN136[OE1]
2	C:SER212[OG]	3.25	B:SER165[O]
3	C:SER168[O]	2.99	B:LYS155[NZ]
4	C:GLN169[OE1]	2.48	B:LEU156[N]

Site I 21h10-hIL-21R interface			
Salt bridges	hIL-21R	Distance [Å]	21h10
1	B:LYS134[NZ]	3.77	A:ASP69[OD1]
2	B:LYS134[NZ]	3.39	A:ASP69[OD2]
3	B:ASP72[OD2]	2.91	A:ARG63[NH1]
4	B:ASP72[OD2]	3.15	A:ARG46[NH1]
5	B:ASP73[OD1]	3.10	A:ARG46[NH1]
6	B:ASP73[OD2]	3.73	A:ARG46[NH2]
7	B:ASP73[OD2]	3.36	A:ARG42[NH2]

Table S5. Mean EC_{50} values from cross-reactivity cell signaling assays.

EC_{50} (M)	Human $CD8^+$ T cells		Murine $CD8^+$ T cells	
	STAT1	STAT3	STAT1	STAT3
**hIL-21**	4.577E-11	4.062E-11	1.179E-08	5.563E-10
**mIL-21**	3.563E-11	2.964E-11	6.227E-11	1.645E-11
**21h10**	9.990E-12	1.030E-11	1.592E-11	5.684E-12

Table S6. Mean EC_{50} values of cell signaling assays.

EC_{50} (M)	Human $CD8^+$ T cells		Murine $CD8^+$ T cells	
	STAT1	STAT3	STAT1	STAT3
**hIL-21**	5.593E-11	3.946E-11	N/M	N/M
**mIL-21**	N/M	N/M	5.073E-11	4.153E-11
**21h10**	2.347E-11	1.475E-11	4.560E-11	2.516E-11

EC_{50} (M)	Human $CD4^+$ T cells		Murine $CD4^+$ T cells	
	STAT1	STAT3	STAT1	STAT3
**hIL-21**	1.447E-11	8.991E-12	N/M	N/M
**mIL-21**	N/M	N/M	4.523E-11	3.675E-11
**21h10**	6.624E-12	3.470E-12	7.015E-11	3.917E-11

EC_{50} (M)	Human B cells		Murine B cells	
	STAT1	STAT3	STAT1	STAT3
**hIL-21**	2.348E-11	9.064E-12	N/M	N/M
**mIL-21**	N/M	N/M	4.198E-11	6.517E-12
**21h10**	1.233E-11	2.078E-12	9.095E-12	2.865E-12

*N/M: not measured

Table S7. Clinical, demographic, and molecular characteristics of patient samples analyzed in the *ex vivo* PDOTS experiment.

Sample #	Tumor Type	Age	Gender	Mutational Status	Tumor mutation burden (absolute count)	Stage	R/NR	*Ex vivo* response
10368	cutaneous melanoma	91	F	N/A	N/A	N/A	NR	R
10387	cutaneous melanoma	35	F	CDKN2A, TERT, BRAF	low	III	NR	NR
10388	cutaneous melanoma	99	M	none	none	III	Tx naïve	NR
10406	cutaneous melanoma	49	M	N/A	N/A	N/A	N/A	NR
10407	cutaneous melanoma	86	M	none	N/A	III	NR	R
10443	cutaneous melanoma	57	M	PTEN, CDKN2A, TERT and KEAP1, BRAF-WT	low	IV	NR	PR
10488	cutaneous melanoma	75	M	TERT and BRAF	low	IV	Tx naïve	PR
10450	cutaneous melanoma	69	M	NRAS, FBXW7	low	III	NR	R
10451	cutaneous melanoma	41	M	PTEN, CDKN2A, TERT and BRAF	low	III	NR	NR
10452	cutaneous melanoma	69	M	DAXX, BRAF and KRAS	low	III	NR	PR
10456	Acral melanoma	80	F	CCND2, CDKN2A and NF1	low	III	NR	NR

SUPPLEMENTARY CODES

Code S1. Parameters for scaffold generation.

```
#SS-size
min_allowed_ss_size['H']=5

#Cluster control
cluster_population_cut_off=100
check_spans_population_cut=20

#Parametric SS size change
target_reduc_size_Nterminus=4
target_extra_size_Nterminus=4
target_reduc_size_Cterminus=4
target_extra_size_Cterminus=4

#Parameters specific for Hs
max_perturbation_angle_H_phi=10.0
max_perturbation_angle_H_psi=10.0
max_perturbation_angle_H_omega=0.0
perturbation_step_H_phi=2.0
perturbation_step_H_psi=2.0
perturbation_step_H_omega=1.0
max_H_phi_curvature_pert=10.0
max_H_psi_curvature_pert=10.0
perturbation_step_H_phi_curvature=2.0
perturbation_step_H_psi_curvature=2.0
max_curv_pitch_aa_H_phi=3
max_curv_pitch_aa_H_psi=3
convergence_parametric_RMSD_H=0.5
max_parametric_RMSD_H=0.6
```

Code S2. PDBInfoLabel for scaffold generation and interface design.

```
REMARK PDBinfo-LABEL:   2 INTERFACE HOTSPOT
REMARK PDBinfo-LABEL:   5 INTERFACE HOTSPOT
REMARK PDBinfo-LABEL:   6 INTERFACE HOTSPOT
REMARK PDBinfo-LABEL:   8 INTERFACE HOTSPOT
REMARK PDBinfo-LABEL:   9 INTERFACE HOTSPOT
REMARK PDBinfo-LABEL:   10 INTERFACE HOTSPOT
REMARK PDBinfo-LABEL:   11 INTERFACE HOTSPOT
REMARK PDBinfo-LABEL:   12 INTERFACE HOTSPOT
REMARK PDBinfo-LABEL:   13 INTERFACE HOTSPOT
REMARK PDBinfo-LABEL:   15 INTERFACE HOTSPOT
REMARK PDBinfo-LABEL:   16 INTERFACE HOTSPOT
REMARK PDBinfo-LABEL:   18 INTERFACE HOTSPOT
REMARK PDBinfo-LABEL:   20 INTERFACE HOTSPOT
REMARK PDBinfo-LABEL:   62 INTERFACE HOTSPOT
REMARK PDBinfo-LABEL:   63 INTERFACE HOTSPOT
REMARK PDBinfo-LABEL:   66 INTERFACE HOTSPOT
REMARK PDBinfo-LABEL:   67 INTERFACE HOTSPOT
REMARK PDBinfo-LABEL:   69 INTERFACE HOTSPOT
REMARK PDBinfo-LABEL:   70 INTERFACE HOTSPOT
REMARK PDBinfo-LABEL:   73 INTERFACE HOTSPOT LOOP
REMARK PDBinfo-LABEL:   74 INTERFACE HOTSPOT LOOP
REMARK PDBinfo-LABEL:   75 INTERFACE HOTSPOT LOOP
REMARK PDBinfo-LABEL:   76 INTERFACE HOTSPOT LOOP
REMARK PDBinfo-LABEL:   77 INTERFACE HOTSPOT LOOP
REMARK PDBinfo-LABEL:   102 INTERFACE HOTSPOT
REMARK PDBinfo-LABEL:   103 INTERFACE HOTSPOT
REMARK PDBinfo-LABEL:   105 INTERFACE HOTSPOT
REMARK PDBinfo-LABEL:   106 INTERFACE HOTSPOT
REMARK PDBinfo-LABEL:   109 INTERFACE HOTSPOT
REMARK PDBinfo-LABEL:   110 INTERFACE HOTSPOT
REMARK PDBinfo-LABEL:   113 INTERFACE HOTSPOT
REMARK PDBinfo-LABEL:   116 INTERFACE HOTSPOT
```
